# Intrinsic backward emission of coherent Raman photons from homogeneous media

**DOI:** 10.1038/s41566-026-01995-z

**Published:** 2026-10-19

**Authors:** Jitendra Nath Acharyya, Gina Benni, Anne Sentenac, Hervé Rigneault, Sandro Heuke

**Affiliations:** Aix Marseille University, CNRS, Centrale Med, Institut Fresnel, Marseille, France

**Keywords:** Nonlinear optics, Nonlinear optics, Biophotonics

## Abstract

In homogeneous materials, scattered light propagates predominantly in the forward direction; thus, collecting signal on the illumination side often relies on interfaces, disorder or multiple scattering. Nonlinear wave mixing can relax this constraint by combining the directions of the driving fields. If the generated wavelength is longer than the driving wavelengths, intrinsic backward emission can become efficient. Here we exploit this regime using coherent Stokes Raman scattering to generate signal directly into the backward hemisphere. Simulations pinpoint high-numerical aperture geometries and polarization states that maximize backward emission, and experiments confirm backward signals from liquids and tissues with detected levels approaching the strength of forward-detected coherent Raman signals. Because the generated signal originates at the focus and remains refocusable, it enables coherent Raman microscopy with same-side confocal collection compatible with fibre coupling and spectrometers when transmission access is impractical. The approach is particularly relevant and beneficial for samples on opaque substrates, thick specimens, tissue biopsies and fibre- or spectrometer-based implementations.

## Abstract (web summary)

Backscattered coherent Raman microscopy brings new opportunities.

## Main

Light propagation in homogeneous media is strongly constrained by momentum conservation^1^. As a consequence, scattered photons cannot easily change direction unless the material provides the required spatial frequencies. In the linear regime, this means that light cannot simply ‘turn a corner’: without abrupt edges, gratings or disorder, backward-directed scattering remains strongly suppressed. This fundamental limitation shapes not only our intuition about how light interacts with matter, but also the design of optical instruments that seek to collect scattered photons efficiently, particularly in settings where only same-side access is available.

Nonlinear optical interactions obey a more flexible form of momentum conservation^2^. By combining the wavevectors of multiple optical fields, they open scattering pathways that are strictly forbidden in the linear regime. Remarkably, this enables coherent photons to be redirected around a corner even in a perfectly homogeneous medium^3^. The ability of nonlinear interactions to provide the missing momentum internally changes the directional constraints of scattering in a profound way: processes such as four-wave mixing can intrinsically generate backward-propagating signal without requiring any structural features of the sample.

**Fig. 1:**
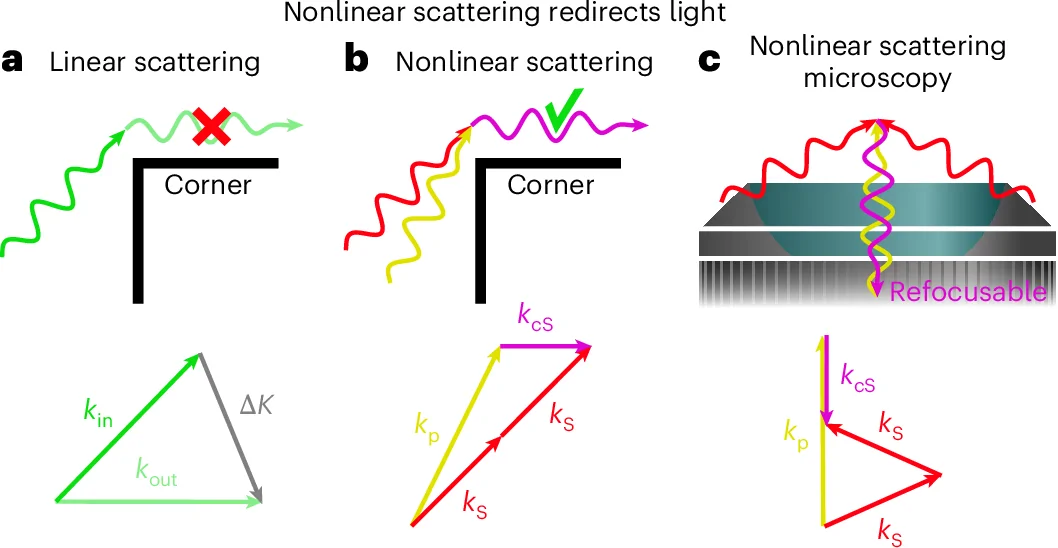
Momentum conservation in linear and nonlinear scattering. **a**, In linear scattering, redirecting light around a corner requires a structured object that supplies the necessary spatial frequency Δ*K* (for example, a grating). **b**, In nonlinear wave mixing, the wavevectors of the driving fields combine to generate the output wavevector *k*_cS_, enabling coherent light to be redirected in a homogeneous medium without high-spatial-frequency sample structure. **c**, In microscopy, this opens a new emission pathway that generates intrinsically backward-emitted, refocusable coherent Raman signal, turning a nominal dark-field configuration into one that yields bright, efficiently collectable signal.

This property has important consequences for coherent Raman microscopy. Conventional coherent Raman signals are predominantly forward-directed^4^, which typically necessitates transmission-side detection. The intrinsic nonlinear redirection mechanism described above offers a fundamentally different route: it allows coherent Raman photons to be generated directly into the backward direction while remaining coherent and refocusable, providing same-side collection without requiring interfaces, disorder or multiple scattering. As illustrated in Fig. 1, nonlinear wave mixing opens a new emission pathway that can generate intrinsically backward-propagating, refocusable coherent Raman photons even when the sample itself is structurally featureless^5,6^.

Here, we demonstrate that coherent Stokes Raman scattering (CSRS) intrinsically supports such backward-directed emission and that this mechanism can be exploited to enable efficient epi-detection from homogeneous samples. CSRS is uniquely suited to this regime because its generated wavelength exceeds the excitation wavelengths, which renders backward phase matching efficient under high-numerical aperture (NA) focusing. By combining experimental measurements with momentum-space simulations, we reveal how nonlinear momentum addition redirects Raman-generated photons and show that this mechanism provides a robust pathway for backward-collected, chemically specific coherent Raman imaging.

## Efficient generation of backscattered CSRS

To identify excitation conditions under which CSRS produces intrinsic backward emission directly from the focal volume, we analyse the momentum-space structure of tightly focused pump and Stokes fields^4,7^. Under high-NA focusing, each beam is described by a broad angular spectrum of plane-wave components^8^. CSRS, as a four-wave mixing process, can combine many such components, enabling backward-directed emission even in homogeneous media. The attainable backscattered signal is jointly set by (1) momentum/phase-matching constraints, (2) polarization selection rules of the isotropic *χ*^(3)^ tensor, and (3) coherent addition of contributing wavevector combinations into the same emission direction^4^. Here, ‘backward CSRS’ refers to CSRS photons emitted into the backward collection cone of a high-NA objective.

**Fig. 2:**
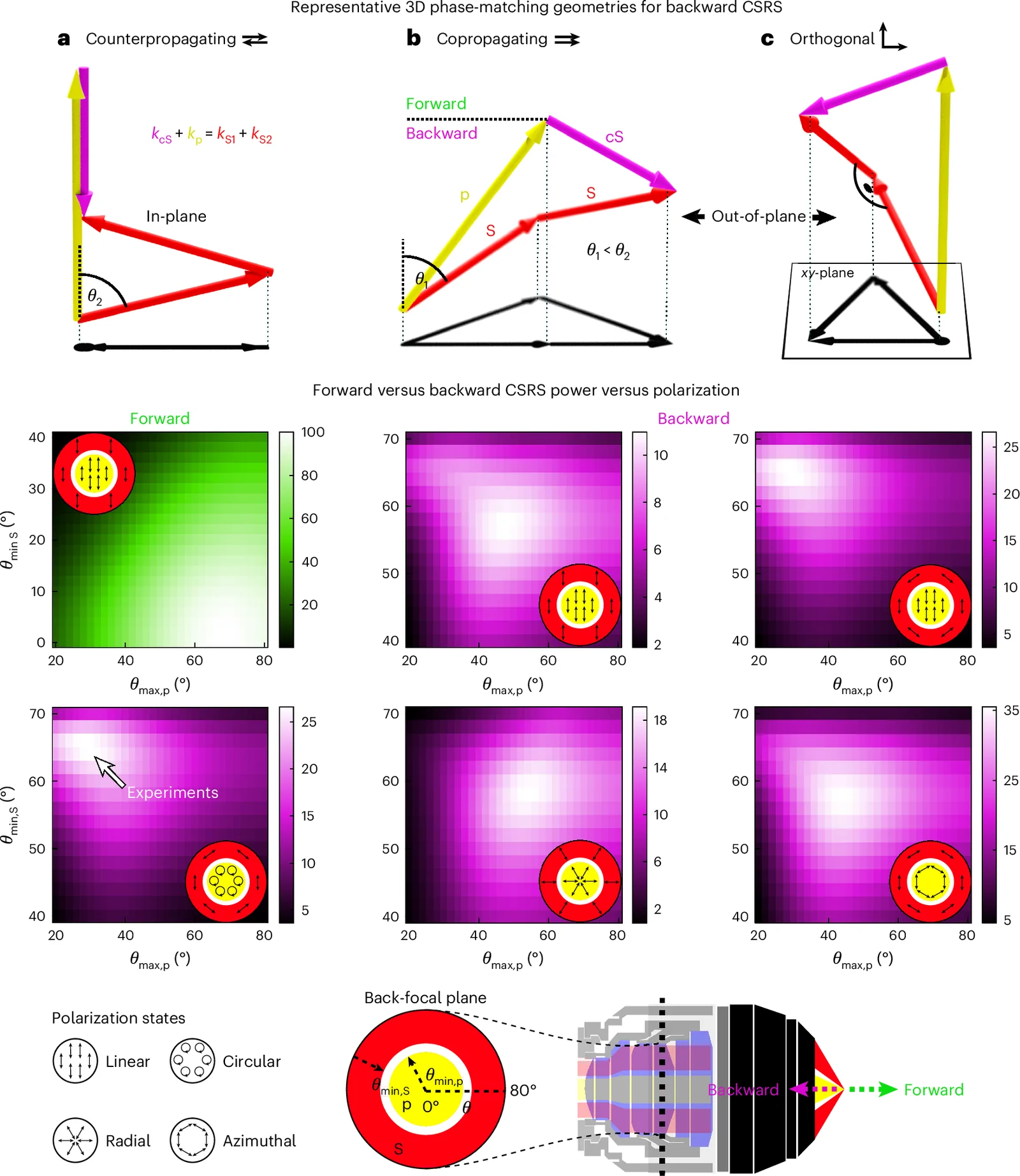
Momentum-space conditions for intrinsic backward CSRS. **a**, Counterpropagating Stokes components favour emission close to the backward optical axis and require steep Stokes angles with a comparatively central pump. **b**, Copropagating Stokes components direct CSRS into the backward half-space at finite polar angles and relax the minimum Stokes-angle requirement. **c**, Orthogonal out-of-plane combinations provide an additional backward-emission pathway enabled by the vectorial focal fields. The bottom panels show forward and backward CSRS power as a function of the maximum pump angle ${\theta }_{\max ,p}$, minimum Stokes angle ${\theta }_{\min ,S}$ and excitation polarization. Pump and Stokes are independently normalized to equal volume-integrated two-photon excitation load, as defined in Supplementary Information section 2.1.1, and all values are referenced to the maximum forward lin–lin CSRS power obtained under the same constraint.

We summarize the dominant momentum-space pathways by three representative configurations defined by their central wavevector geometry (Fig. 2). Throughout, the labels ‘counterpropagating’, ‘copropagating’ and ‘orthogonal’ refer to the relative orientation of the transverse components of the two interacting Stokes wavevectors in the focal angular spectrum. In the counterpropagating Stokes configuration, the two Stokes wavevectors enter under steep polar angles and are nearly opposite in direction, while phase matching favours a pump incidence close to the optical axis. This in-plane geometry directs CSRS close to the backward optical axis and, in isotropic homogeneous samples, often provides a favourable projection onto the nonvanishing isotropic *χ*^(3)^ contractions. Its main limitation is geometric: strong contributions preferentially draw on very steep Stokes angles.

In the copropagating configuration, the Stokes wavevectors are nearly parallel and approach the focus at high incidence angles; backward CSRS then emerges at finite polar angles within the backward half-space (Fig. 2). Compared with the counterpropagating case, this out-of-plane pathway can substantially reduce the Stokes polar angles required for backward emission. Emphasizing predominantly copropagating triplets (pump and 2 Stokes) typically requires annular Stokes illumination and, if one aims to suppress counterpropagating contributions, shifting the pump spectrum towards higher angles as well (dual-ring pupil illumination).

In the orthogonal configuration, the Stokes wavevectors differ in azimuth by approximately 90°, while the pump wavevector is nearly perpendicular to both (Fig. 2). Near-orthogonal out-of-plane triplets occur naturally in the angular spectrum and also relaxes the Stokes polar-angle requirement under tight focusing while still fulfilling the momentum condition for backward emission. In homogeneous isotropic samples, these pathways are often less efficient than their momentum geometry alone would suggest because the resulting focal-field vectors can yield a comparatively small projection onto the nonvanishing isotropic *χ*^(3)^ contractions; they are expected to benefit most when local symmetry is reduced, for example at interfaces or in anisotropic structures.

To substantiate these trade-offs, we performed full-vectorial simulations of the focused pump and Stokes fields for isotropic samples and computed the resulting CSRS emission (Supplementary Information section 2). To represent operation with high-energy pulses, the pupil geometries in Fig. 2 are compared at equal volume-integrated two-photon excitation load. The definition of this conservative constraint and its comparison with equal input power and equal volume-integrated three-photon excitation load are given in Supplementary Information section 2.1.1 and Supplementary Fig. 6. All backward signals are referenced to the maximum forward CSRS signal obtained under the same constraint.

Under the two-photon-load constraint, purely linear excitation yields its strongest backward CSRS for maximum pump angles around 45^∘^–50^∘^ and minimum Stokes angles around 55^∘^–60^∘^ (Fig. 2). Combining azimuthally polarized Stokes excitation with a linear or circular pump more than doubles the backward signal and reaches approximately 25% of the maximum forward-emitted CSRS power. Fully azimuthal excitation produces still larger total backward power, approaching 35%, but refocuses into an annular image-plane mode; circular-pump/azimuthal-Stokes excitation was therefore selected experimentally as the better compromise between backward-emitted power and compact, single-lobed refocusing (Supplementary fig 5).

Finally, we tested whether steep-angle pathways can be strengthened by restricting both beams to annular illumination (dual-ring excitation; Supplementary Information section 2). Dual-ring illumination can approach, but does not exceed, the performance achieved with a centre-filled pump combined with steep-angle Stokes illumination. For completeness, we note that analogous high-angle and orthogonal wavevector combinations can, in principle, also bend coherent anti-Stokes Raman scattering (CARS) towards the backward half-space; however, the corresponding emission remains confined to very high angles and therefore falls outside the collection cone of realistic microscope objectives, in contrast to backward CSRS.

## Experimental results

Guided by the theoretical analysis presented above, we implemented an excitation geometry optimized for the direct generation of backward-directed CSRS. The Stokes beam at 1,030 nm was shaped into an azimuthally polarized ring filling the outer annulus of the objective pupil, while the circularly polarized pump beam at approximately 790 nm was confined to the central pupil region.

**Fig. 3:**
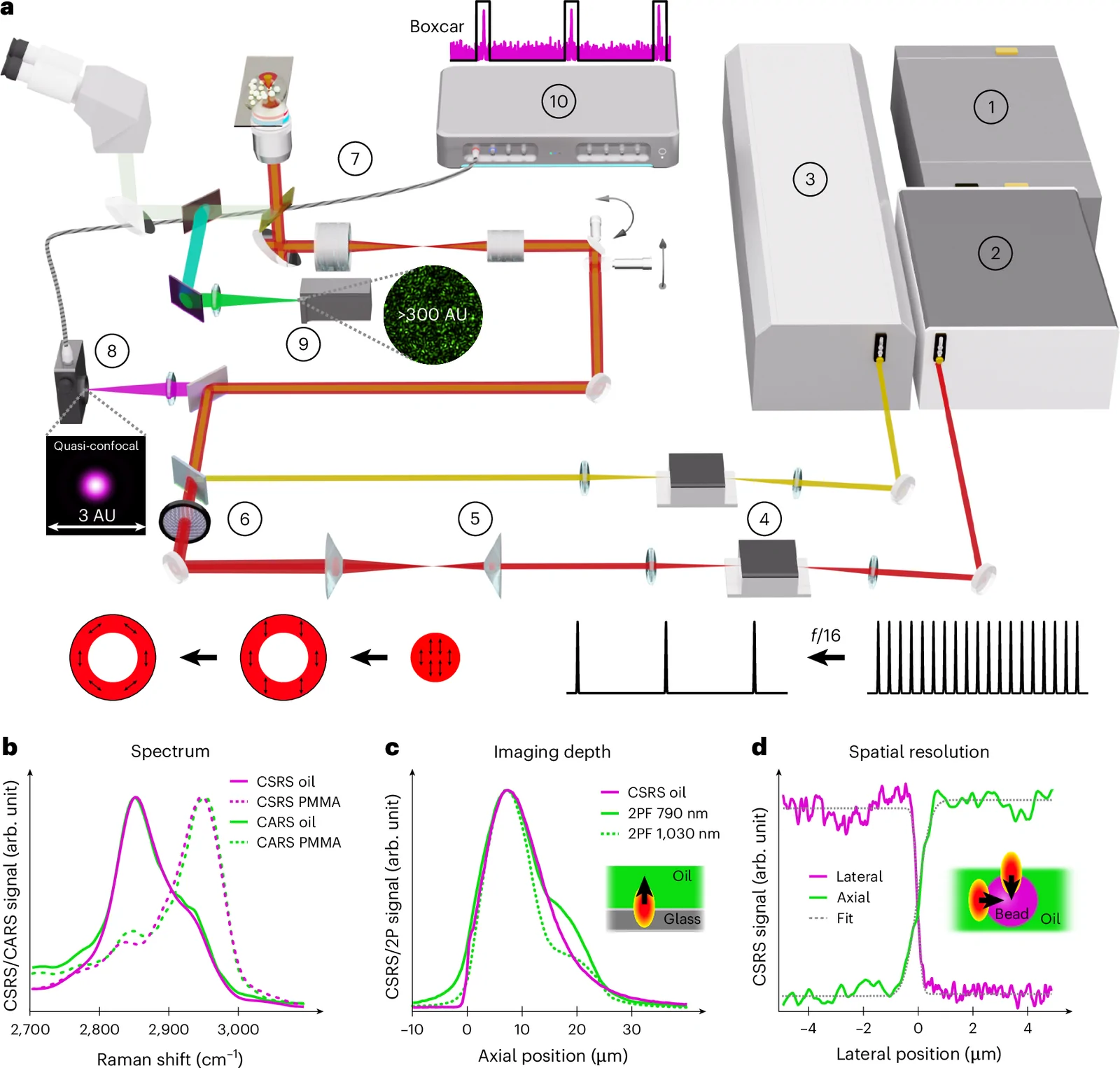
Experimental set-up and characterization. **a**, Schematic of the experiment: (1) Yb pump laser; (2) amplifier; (3) optical parametric oscillator (OPO); (4) acousto-optic modulator (AOM); (5) double axicon for Stokes ring shape illumination; (6) vortex plate for Stokes azimuthal polarization; (7) laser-scanning microscope (LSM); (8) InGaAs avalanche photodiode (APD); (9) photomultiplier tube (PMT); (10) lock-in amplifier. The PMT features a much larger detection area than an Airy disk (1 Airy unit, AU), while the APD provides a quasi-confocal detection of the CSRS signal. **b**, CSRS spectra of oil and PMMA obtained by tuning the pump wavelength. **c**, Depth profiles of CSRS in oil and two-photon fluorescence (2PF) of yellow marker dissolved in oil. The depth limitation of backscattering CSRS arises from the aberration corrections of the TIRF objective and similarly affects two-photon fluorescence. **d**, CSRS lateral profile extracted from a PMMA–sunflower-oil interface. The error-function fit to the edge-spread function yields an effective lateral line-spread-function FWHM of approximately 330 nm. Axial edge measurements across the same type of interface yielded an effective axial line-spread-function FWHM of approximately 0.92 μm.

To enhance the nonlinear signal without increasing the average power delivered to the sample, we reduced the effective repetition rate of both beams to 0.71 MHz using a pulse-picking scheme (Fig. 3a). In addition, boxcar gating of the InGaAs avalanche photodetector reduced the effective dark noise by approximately one order of magnitude. Further details about the experimental implementation are presented within Supplementary Information section 1.

As a first quantitative benchmark, we compared the signal strength of forward CARS with backward CSRS. Detailed photon-flux, detector-noise and signal-to-noise ratio calculations are provided in Supplementary Information section 3. Under excitation conditions identical to those used in the simulations and calibrated using detector specifications, we estimate a directly backscattered CSRS photon flux of approximately 7 × 10^9^ photons s^−1^ emitted from the focal volume in sunflower oil. Switching from purely linear excitation to azimuthally polarized Stokes illumination combined with a circular polarized pump increased the CSRS signal by about 50%, consistent with the predicted polarization dependence.

To rule out any contribution from forward-generated CSRS reflected into the detection path, we placed a mirror directly above the sample. This produced no measurable increase in the detected CSRS photon flux, supporting an intrinsically backward-emitted origin. Any residual forward CSRS under annular Stokes illumination is further suppressed by the small effective collection area of the avalanche photodetector (~3 Airy units), which acts as a confocal pinhole.

For comparison, forward-generated CARS photons can be detected in the backward direction when a mirror is placed above the sample, yielding photon fluxes of approximately 4 × 10^10^ photons s^−1^ (Supplementary Information section 3). In realistic imaging scenarios involving thick or heterogeneous samples, however, epi-detected CARS signals arise either from high spatial frequencies at refractive-index interfaces or from forward-generated CARS that is multiply scattered and back-reflected^4,9^. Such back-reflected CARS cannot be refocused onto the detector plane, precluding efficient fibre coupling, amplification, spectral analysis or accurate point-spread-function characterization. By contrast, backward CSRS is generated directly into the backward hemisphere and remains refocusable, enabling true confocal detection. For the experimentally used excitation geometry, our simulations show that the backward CSRS field refocuses after the objective–tube-lens relay into a compact, near-Gaussian intensity distribution, supporting fibre- or spectrometer-based collection (Supplementary Fig. 5).

**Fig. 4:**
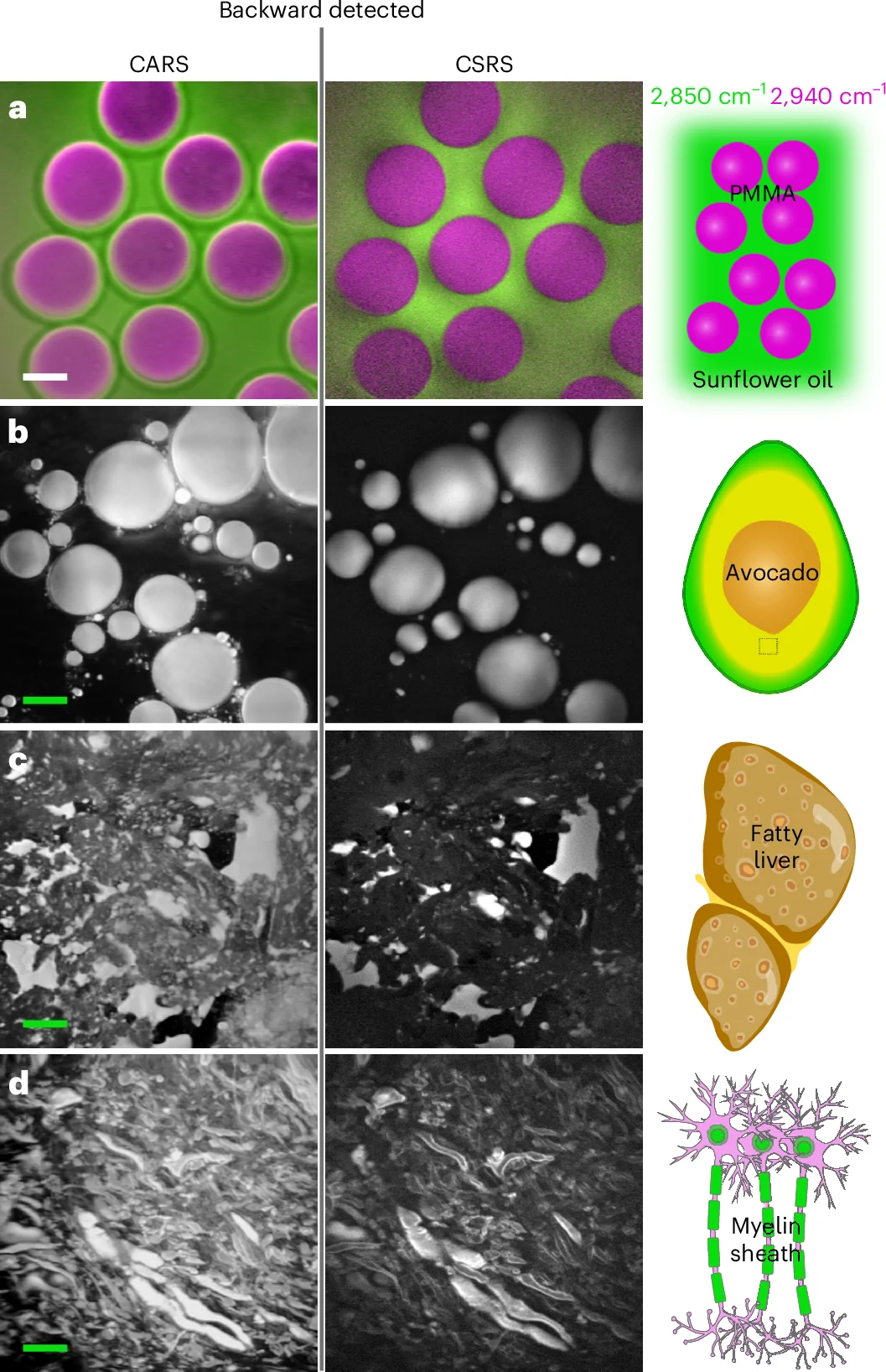
Experimental comparison of epi-CARS and backscattered CSRS imaging. **a**, Twenty-micrometre PMMA beads dispersed in sunflower oil; green and purple correspond to Raman resonances at 2,850 cm^−1^ and 2,940 cm^−1^, respectively. **b**–**d**, Avocado flesh (**b**), human fatty liver tissue (**c**) and myelin sheaths in sheep brain tissue (**d**), all imaged at 2,850 cm^−1^. All scale bars, 10 μm. Effective pixel dwell time: 20 μs. Image acquisition time: 21 s for a 1,024 × 1,024-pixel image. Pump and Stokes beam powers at the sample were 2.5 mW and 6 mW, respectively.

Figure 4a shows backscattered CSRS images of polymethyl methacrylate (PMMA) beads dispersed in sunflower oil. The corresponding spectra (Fig. 3b) confirm that, in the absence of electronic resonances, CARS and CSRS provide nearly interchangeable vibrational information^10,11^. Notably, strong CSRS signals are obtained from homogeneous regions of sunflower oil, well away from any bead–oil or oil–substrate interfaces. In contrast to epi-CARS, the PMMA beads do not exhibit interface-related shadowing or highlighting effects, consistent with the absence of high-pass filtering in backward CSRS.

To assess a possible contribution of high spatial frequencies at the coverslip–oil interface to the detected CSRS signal, we investigated the axial signal dependence using a total internal reflection fluorescence (TIRF) objective (Fig. 3c). Such objectives are designed to form a diffraction-limited focus at the coverslip interface, but the focus quality degrades rapidly with increasing depth^12^. When imaging deeper into the sample, it is therefore essential to distinguish between limitations imposed by CSRS phase matching and those arising from point-spread-function degradation.

We further quantified the spatial response from edge measurements at PMMA–oil interfaces. The lateral edge response yields an effective lateral line-spread-function full width at half maximum (FWHM) of approximately 330 nm (Fig. 3d). In addition, axial edge profiles acquired across the PMMA–oil interface yielded an effective axial line-spread-function FWHM of approximately 0.92 μm. These values should be interpreted as edge-derived effective imaging resolutions rather than as a direct measurement of the local nonlinear source distribution alone. In particular, the axial edge response includes coherent CSRS generation, backward propagation, collection through the high-NA objective, and confocal detection.

Having established backward CSRS on well-defined test samples, we next assessed its performance on a biologically relevant yet structurally simple specimen. Avocado flesh provides an intermediate system in which lipid droplets introduce pronounced refractive-index variations while maintaining a comparatively simple microstructure. Figure 4b compares backward CSRS with epi-CARS at the lipid resonance at 2,850 cm^−1^. Backward CSRS remains robust despite refractive-index mismatches between lipid droplets and the surrounding tissue, which effectively reduce the illumination NA^13^. Moreover, a fluorescence background is observed in the epi-CARS channel^14^—even when using a narrowband filter—whereas no such contribution is detected in the CSRS channel at 1,470 nm (Supplementary Fig. 1 and Supplementary Information section 1).

We finally applied backward CSRS to more demanding biological specimens characterized by strong scattering and fine-scale organization. Figure 4c shows backward CSRS imaging of human fatty liver tissue at the lipid resonance at 2,850 cm^−1^. Lipid droplets and surrounding tissue can be clearly distinguished. Fatty liver disease is clinically highly relevant, as hepatic lipid accumulation constitutes an early and potentially reversible stage of metabolic liver disorders^15^. Notably, the brightness difference between lipid-rich regions and surrounding tissue is reduced in epi-CARS compared with backward CSRS. This difference is consistent with the distinct spatial transfer functions of the two epi-detected signals. In thick tissue, epi-CARS generally contains a mixture of true backward-emitted CARS generated by high-spatial-frequency structures near the focus and originally forward-emitted CARS that is subsequently scattered or reflected back toward the objective. Consequently, epi-CARS images can be influenced by interfaces, local scattering, sample reflectivity and return-path efficiency. This behaviour has been observed directly in spinal cord tissue, where forward- and epi-detected CARS provide related but visibly different myelin contrast and line profiles^16^. By contrast, backward CSRS is generated directly into the backward hemisphere by nonlinear phase matching and therefore provides bulk chemical contrast without relying on high-pass spatial filtering or scattering-mediated return.

Figure 4d highlights backward CSRS imaging of brain tissue at the lipid resonance at 2,850 cm^−1^. The bright ellipsoidal structures correspond to myelin sheaths, which are lipid-rich and provide electrical insulation along neuronal pathways. Damage to these structures underlies several neurological disorders, including multiple sclerosis^17^ and Guillain–Barré syndrome^18^. Efficient, label-free imaging of myelin therefore represents a key capability for advancing research into demyelinating diseases.

## Discussion

We begin by placing backward-directed CSRS in the broader context of coherent Raman signal generation and detection. In particular, we compare the experimentally observed signal levels of backward CSRS with those of forward CSRS and conventional forward-detected CARS, and relate these values to expectations derived from numerical simulations.

Our simulations predict that, under otherwise identical excitation conditions and high-NA focusing, forward CARS is approximately one order of magnitude stronger than forward CSRS in terms of emitted power (Supplementary Information section 2). This difference arises primarily from the large wavelength separation between the anti-Stokes and coherent Stokes signals. In a homogeneous medium, the coherent Raman source is spatially extended, with an effective size on the order of 1 μm. In this regime, the emission is better described by Mie-type scattering rather than by point-scatterer models^19^. Consistent with this picture, our simulations yield an effective wavelength-scaling exponent of *n* ≈ 2.8, corresponding to an experimentally observed forward CARS-to-CSRS photon-flux ratio of approximately five in the CH-stretch region.

Importantly, this photon-flux difference should not be interpreted as a smaller molecular cross-section for CSRS. Under electronically non-resonant conditions, CARS and CSRS are governed by the same Raman-active vibrational response and provide equivalent spectral information^3,11^. The lower photon flux obtained for backward CSRS instead reflects optical factors, including the longer generated wavelength, the backward phase-matching geometry, and finite collection and detection efficiencies, which are included in the emitted and detected photon budgets quantified in Supplementary Information sections 2 and 3.

Under the volume-integrated two-photon-load constraint used in Fig. 2, the experimentally selected circular-pump/azimuthal-Stokes geometry produces backward CSRS at approximately one-quarter of the maximum forward-CSRS power. This ratio is not universal: equal input power gives a lower relative backward efficiency, whereas the volume-integrated three-photon-load constraint favours the high-intensity annular geometries more strongly than the two-photon model (Supplementary Information section 2.1.1). Combined with the approximately fivefold forward CARS-to-CSRS photon-flux ratio, the selected conservative nonlinear-exposure constraint corresponds to an overall reduction of roughly twenty in backward-CSRS photon flux relative to forward CARS.

Importantly, this remaining photon-flux disadvantage is partially compensated in practice by detector performance. Forward CARS is typically detected using photomultiplier tubes, which exhibit quantum efficiencies on the order of 20% in the visible range. By contrast, InGaAs avalanche photodiodes used for CSRS detection at wavelengths around 1,500 nm routinely reach quantum efficiencies exceeding 80%. As a result, the experimentally detected photon flux differs by only a factor of approximately 5 between forward CARS and backward CSRS, despite the substantially lower number of photons emitted into the backward hemisphere. This distinction is essential, as the detected photon flux ultimately governs the achievable signal-to-noise ratio. Experimentally, we obtain a signal-to-noise ratio of 49 for backward CSRS and 171 for forward CARS at an equivalent 1 μs integration time in homogeneous sunflower oil at 2,850 cm^−1^ (Supplementary Information section 3 and Supplementary Fig. 7), suggesting that the present backward-CSRS implementation is still detector-noise dominated. While photomultiplier tubes readily enable shot-noise-limited detection for forward CARS, backward CSRS is currently limited by the noise performance of available near-infrared detectors^20^. Nevertheless, several realistic routes exist to substantially improve detection-limited performance. Faster detectors would permit narrower temporal gates and further suppress electronic noise, heterodyne detection schemes^21^ could coherently amplify the CSRS signal above the noise floor, and nonlinear frequency conversion^22^ of CSRS photons into the visible range would enable the use of low-noise visible detectors. Together, these approaches indicate that the current limitations of backward CSRS are technical rather than fundamental.

Beyond detector performance, a second technical limitation concerns the availability of suitable objective lenses. Backward CSRS benefits from large excitation angles and high transmission at wavelengths around 1,500 nm, yet objective lenses combining these properties with long working distances remain scarce^23^. Notably, diffraction-limited point-spread functions are not a strict requirement for backward CSRS. This relaxation of conventional resolution constraints suggests that objective designs optimized specifically for backward CSRS could substantially improve practical performance.

Operating at wavelengths around 1,500 nm also offers intrinsic advantages for coherent Raman imaging. In particular, fluorescence—which frequently contaminates CARS measurements in the visible—is entirely absent in the CSRS detection channel^24^ for all samples investigated here. This property enhances chemical specificity and robustness, especially in complex biological environments.

In conclusion, we have demonstrated a coherent Raman microscopy modality capable of generating and detecting intrinsically backward-emitted CSRS from homogeneous samples. By exploiting nonlinear momentum addition in four-wave mixing and the large wavelength separation between pump, Stokes and CSRS fields, we overcome a fundamental restriction that suppresses backward scattering in both linear optics and conventional CARS. Our simulations identify steep, azimuthally polarized Stokes excitation combined with a centrally focused pump as an efficient route to backward emission, and our experiments confirm the resulting photon budgets and imaging performance.

Although backward CSRS emits fewer photons than forward CARS, the combination of favourable detector quantum efficiencies and refocusable backward emission enables efficient confocal detection, fibre coupling, spectroscopic analysis and point-spread-function characterization. Its practical niche is therefore not to replace forward CARS or transmission-detected SRS when transmission access is available, but to provide a directly generated and refocusable coherent Raman signal for same-side detection. This is particularly relevant for samples on opaque substrates, thick specimens, tissue biopsies and fibre- or spectrometer-based implementations. In laser-scanning spectroscopy, backward CSRS also avoids the need for a separate transmission-side descanned detection path, which is otherwise required to efficiently couple scanned forward CARS or SRS signals into a fixed fibre or spectrometer entrance. This work establishes backward CSRS as a new contrast mechanism for coherent Raman microscopy and opens a pathway towards robust, epi-detected and fibre-compatible Raman imaging platforms for applications where transmission-based detection is impractical or technically cumbersome.

## Online content

Any methods, additional references, Nature Portfolio reporting summaries, source data, extended data, supplementary information, acknowledgements, peer review information; details of author contributions and competing interests; and statements of data and code availability are available at https://doi.org/10.1038/s41566-026-01995-z.

## Supplementary information

Supplementary Information Supplementary Methods, Figs. 1–7, Table 1 and References.

Peer Review File

## Data Availability

The data that support the findings of this study are available from the corresponding author upon request.

## References

[1] Born, M. & Wolf, E. *Principles of Optics: Electromagnetic Theory of Propagation, Interference and Diffraction of Light* 7th edn (Cambridge Univ. Press, 1999).

[2] Boyd, R. W. *Nonlinear Optics* 4th edn (Academic Press, 2020).

[3] Heuke, S. & Rigneault, H. Coherent Stokes Raman scattering microscopy (CSRS). *Nat. Commun.* **14**, 3337 (2023). 10.1038/s41467-023-38941-4 PMC10247746 37286641

[4] Cheng, J.-X., Volkmer, A. & Xie, X. S. Theoretical and experimental characterization of coherent anti-Stokes Raman scattering microscopy. *J. Opt. Soc. Am. B* **19**, 1363–1375 (2002).

[5] Heuke, S. & Rigneault, H. Laser scanning dark-field coherent anti-Stokes Raman scattering (DF-CARS): a numerical study. *Opt. Express* **29**, 3985–3995 (2021). 10.1364/OE.414972 33770987

[6] Volkmer, A., Cheng, J.-X. & Xie, X. S. Vibrational imaging with high sensitivity via epidetected coherent anti-Stokes Raman scattering microscopy. *Phys. Rev. Lett.* **87**, 023901 (2001).

[7] Hashimoto, M. & Araki, T. Three-dimensional transfer functions of coherent anti-Stokes raman scattering microscopy. *J. Opt. Soc. Am. A* **18**, 771–776 (2001). 10.1364/josaa.18.000771 11318327

[8] Richards, B. & Wolf, E. Electromagnetic diffraction in optical systems. II. Structure of the image field in an aplanatic system. *Proc. R. Soc. Lond. Ser. A* **253**, 358–379 (1959).

[9] Schie, I. W., Weeks, T., McNerney, G. P. & Huser, T. Simultaneous forward and epi-CARS microscopy with a single detector by time-correlated single photon counting. *Opt. Express* **16**, 2168–2175 (2008). 10.1364/oe.16.002168 18542297

[10] Zheng, J. B., Leipertz, A., Snow, J. B. & Chang, R. K. Simultaneous observation of rotational coherent Stokes Raman scattering and coherent anti-Stokes Raman scattering in air and nitrogen. *Opt. Lett.* **8**, 350–352 (1983). 10.1364/ol.8.000350 19718111

[11] Bito, K. et al. Three-pulse multiplex coherent anti-Stokes/Stokes Raman scattering (CARS/CSRS) microspectroscopy using a white-light laser source. *Chem. Phys.* **419**, 156–162 (2013).

[12] Gibson, S. F. & Lanni, F. Experimental test of an analytical model of aberration in an oil-immersion objective lens used in three-dimensional light microscopy. *J. Opt. Soc. Am. A* **9**, 154–166 (1992). 10.1364/josaa.9.000154 1738047

[13] Török, P., Varga, P., Laczik, Z. & Booker, G. R. Electromagnetic diffraction of light focused through a planar interface between materials of mismatched refractive indices: an integral representation. *J. Opt. Soc. Am. A* **12**, 325–332 (1995).

[14] Ly, S., McNerney, G., Fore, S., Chan, J. & Huser, T. Time-gated single photon counting enables separation of CARS microscopy data from multiphoton-excited tissue autofluorescence. *Opt. Express* **15**, 16839–16851 (2007). 10.1364/oe.15.016839 19550974

[15] European Association for the Study of the Liver (EASL), European Association for the Study of Diabetes (EASD) & European Association for the Study of Obesity (EASO), EASL–EASD–EASO clinical practice guidelines for the management of non-alcoholic fatty liver disease. *J. Hepatol.* **64**, 1388–1402 (2016). 10.1016/j.jhep.2015.11.004 27062661

[16] Wang, H., Fu, Y., Zickmund, P., Shi, R. & Cheng, J.-X. Coherent anti-Stokes Raman scattering imaging of axonal myelin in live spinal tissues. *Biophys. J.* **89**, 581–591 (2005). 10.1529/biophysj.105.061911 PMC1366558 15834003

[17] Dobson, R. & Giovannoni, G. Multiple sclerosis. *Nat. Rev. Dis. Primers* **4**, 43 (2018).

[18] Willison, H. J., Jacobs, B. C. & van Doorn, P. A. Guillain–Barré syndrome. *Lancet* **388**, 717–727 (2016). 10.1016/S0140-6736(16)00339-1 26948435

[19] Bohren, C. F. & Huffman, D. R. *Absorption and Scattering of Light by Small Particles* (John Wiley & Sons, 1983).

[20] Zhu, B. & Jonathan, H. A review of image sensors used in near-infrared and shortwave infrared fluorescence imaging. *Sensors* **24**, 3539 (2024). 10.3390/s24113539 PMC11175340 38894330

[21] Potma, E. O., Evans, C. L. & Xie, X. S. Heterodyne coherent anti-Stokes Raman scattering (CARS) imaging. *Opt. Lett.* **31**, 241–243 (2006). 10.1364/ol.31.000241 16441043

[22] Tidemand-Lichtenberg, P. & Pedersen, C. Long-wavelength, high-resolution microscopy using upconversion in ultra-thin crystals. *APL Photonics* **9**, 096102 (2024).

[23] Keatings, S. R., Zhang, W. & McConnell, G. Characterization of microscope objective lenses from 1,400 to 1,650 nm to evaluate performance for long-wavelength nonlinear microscopy applications. *Microsc. Res. Technique* **71**, 517–520 (2008). 10.1002/jemt.20580 18314867

[24] Zhu, S. et al. Near-infrared-II (NIR-II) bioimaging via off-peak NIR-I fluorescence emission. *Theranostics* **8**, 4141–4151 (2018). 10.7150/thno.27995 PMC6096392 30128042

