# Supplementary material for: Intrinsic backward emission of coherent Raman photons from homogeneous media
Source: Nat Photonics. 2026 Oct 19;20(11):1318–24. doi: 10.1038/s41566-026-01995-z (PMC13747816; doi:10.1038/s41566-026-01995-z)
Supplement: Supplementary file 1 [file 41566_2026_1995_MOESM1_ESM.pdf]

nature photonics

Supplementary information

https://doi.org/10.1038/s41566-026-01995-z

# Intrinsic backward emission of coherent Raman photons from homogeneous media

In the format provided by the authors and unedited

# Supplementary information to: Intrinsic backward emission of coherent Raman photons from homogeneous media

JITENDRA NATH ACHARYYA[1], GINA BENNI[1], ANNE SENTENAC[1], HERVÉ RIGNEAULT[1], SANDRO HEUKE[1,*]

[1] *Aix Marseille University, CNRS, Centrale Med, Institut Fresnel, 13013 Marseille, France.*
[*] *Corresponding authors: Sandro.Heuke@fresnel.fr*

**Abstract:** In this Supplementary Information, we detail the experimental methods, theoretical framework, and numerical approaches used to analyze backward-directed coherent Stokes Raman scattering (CSRS) and quantify its expected photon budget. Beyond modeling the vectorial focused excitation fields and the resulting third-order nonlinear polarization, we use a Green-function formulation to obtain the emitted CSRS field and evaluate how illumination geometry, polarization, and numerical aperture jointly determine backward-emission efficiency. Separately, to connect the expected signal levels to detector performance, we compute the photon flux, noise-equivalent photon flux, and resulting signal-to-noise ratio for representative detection schemes. These analyses provide the foundation for interpreting the measurements in the main text and guide the optimization of backward-CSRS microscopy for high-sensitivity imaging.

## 1. Methods

### *1.1. Experimental setup*

A custom-built laser scanning microscope was developed to enable epi-detection of Coherent Stokes Raman Scattering (CSRS). The excitation source consisted of a Yb-based femtosecond oscillator (FLINT, Light Conversion; 11.32 MHz) coupled to an optical parametric oscillator (Alpha, Stuttgart Instruments). The fundamental output of the oscillator, after spectral cropping and amplification, provided the Stokes beam centered at 1030 nm (1.5 ps), while the picosecond OPO (1.5 ps) delivered a tunable visible output that served as the pump beam. For the present experiments, the Stokes beam at 1030 nm and the pump between 786 and 800 nm were used to resonantly excite the CH stretching vibrations around 2850-3000 $cm^{-1}$. To enhance the CSRS signal further, we employ acousto-optical modulators (AOM, AA, MT200-A0,5-800) in the pump and Stokes beam path in combination with laser-synchronized arbitrary waveform generator (Liquid instruments, Moku:Pro) as a pulse picker to artificially reduce effective laser repetition rate by 16 to 0.707 MHz. As a result, the overall number of CARS and CSRS scattered photons is increased by 256 for the same average power allowing for significantly faster imaging in a constant noise regime [1].

Unlike conventional forward-detected coherent Raman modalities, CSRS in a backscattering geometry requires extremely high-angle Stokes excitation. To achieve this, the Stokes beam (1030 nm) was transformed into a thick ring (1/3 of the final ring radius is filled) using a pair of identical axicons [2, 3] (Thorlabs, AX2520M-B). Please note that the Stokes ring would blur and lose its intended diameter and ring-width ratio during free-space propagation toward the galvo mirrors; therefore, it was relayed and demagnified by a 4f-imaging system ($f_1/f_2 = 3.3$) to match the backaperture size of the objective lens. The pump and Stokes beams are combined and spatially overlapped before the microscope by a dichroic long-pass filter (Edmund Optics, 87-045). The combined beams were subsequently directed onto a pair of galvanometric scanners via an additional dichroic mirror (Thorlabs, DMLP1180R), which reflected the excitation beams while transmitting the generated CSRS signal along the descanning path. The temporal synchronization between pump and Stokes beams was achieved with a mechanical delay line.

The scanning optics comprised galvo scanners (Edmund, 16-040), a scan lens (Thorlabs, SL50-2P2), and a tube lens (Thorlabs, TL200-2P2), providing a fourfold beam expansion and imaging the galvo plane onto the objective back pupil plane. This ensured that the outer annulus of the high-NA objective (Nikon Apo TIRF 60×, NA = 1.49, oil immersion) was filled by the Stokes beam, while the pump occupied the central aperture region. For optimum signal generation, we shaped the initially linear polarization of the Stokes beam into azimuthal polarization by a vortex retarder (Thorlabs, WPV10L-980).

The backscattered CSRS signal was collected through the same objective in descanning mode. Residual pump and Stokes beams were suppressed using a long-pass filter (Thorlabs FELH1400), and the CSRS photons were detected with an InGaAs avalanche photodiode (Thorlabs APD410C). The APD output was processed with a digital boxcar (time-gated) integrator implemented on a Moku:Pro platform (Liquid Instruments). The detection was synchronized to the pulse-picked repetition rate of 0.707 MHz and a 45 ns gate width was used, reducing the contribution of APD dark noise by a factor of $\sim 30$ [4]. We measured the transmission of our 60× objective lens at 1500 nm to be approximately 45% with a theoretical total transmission from the sample to the detector of 30% accounting for the known transmission coefficients of all intermediate lenses and mirrors. CARS radiation was collected in backward direction at a non-descanned position using a dichroic mirror (Semrock, FF705-Di01-25x36) and a photomultiplier tube (Thorlabs, PMT1001). Forward CARS (and CSRS) signal was collected by placing a mirror just on top of the sample. The image acquisition was performed using *ScanImage* (Vidrio, free version), controlled by two synchronized NI DAQ cards (PCIe-6374 & PCIe-6321).

### 1.2. Sample preparation

*PMMA bead model sample:* As a reference specimen, poly(methyl methacrylate) (PMMA) microspheres with a nominal diameter of 20 µm (Sigma-Aldrich, analytical standard, product no. 90549-5ML-F) were dispersed in sunflower oil, providing a homogeneous, lipid-rich matrix with approximate refractive-index matching suitable for backscattering CSRS. The suspension was loaded into a circular silicone spacer with adhesive backing (Thermo Fisher Scientific / Invitrogen, Press-to-Seal silicone spacer; 20 mm diameter, 0.5 mm depth), and sealed between two borosilicate glass coverslips (VWR, No. 1 thickness, 25 mm diameter, 130 µm nominal thickness). This bead-in-oil preparation was used for CSRS imaging of the beads (Fig. 4a) and also to generate the lateral transition profile across a PMMA–oil interface (Fig. 3d), whereas homogeneous sunflower oil regions were used as the "bulk" reference for the CSRS curves shown in Fig. 3b–c.

*Axial calibration with two-photon fluorescence:* To experimentally assess axial (z-axis) probing performance, we performed two-photon fluorescence (2PF) calibration measurements using a fluorescent yellow marker dye. A dilute dye suspension was prepared in sunflower oil such that isolated fluorescent droplets were distributed throughout the 0.5 mm-thick spacer volume. In addition, dye was deposited onto the inner surface of the top coverslip to define a sharp axial reference plane. The resulting 2PF depth response was compared to the CSRS depth dependence measured in sunflower oil (Fig. 3c).

*Avocado tissue, human fatty liver, and sheep brain tissue:* Fresh avocado tissue served as a lipid-rich bio-reference sample in the CH-stretch region: a few-millimeter-thick slice from the inner avocado flesh was placed directly on a glass coverslip and measured without further processing (Fig. 4b). To preserve native biochemical composition, all biological specimens were analyzed without chemical fixation or staining. Human fatty liver tissue sections (10 µm thickness) were provided by clinical partners and measured in their untreated state (Fig. 4c). Fresh sheep brain tissue was obtained from a local butcher, cut into slices of a few millimeters thickness, and measured immediately after preparation (Fig. 4d).

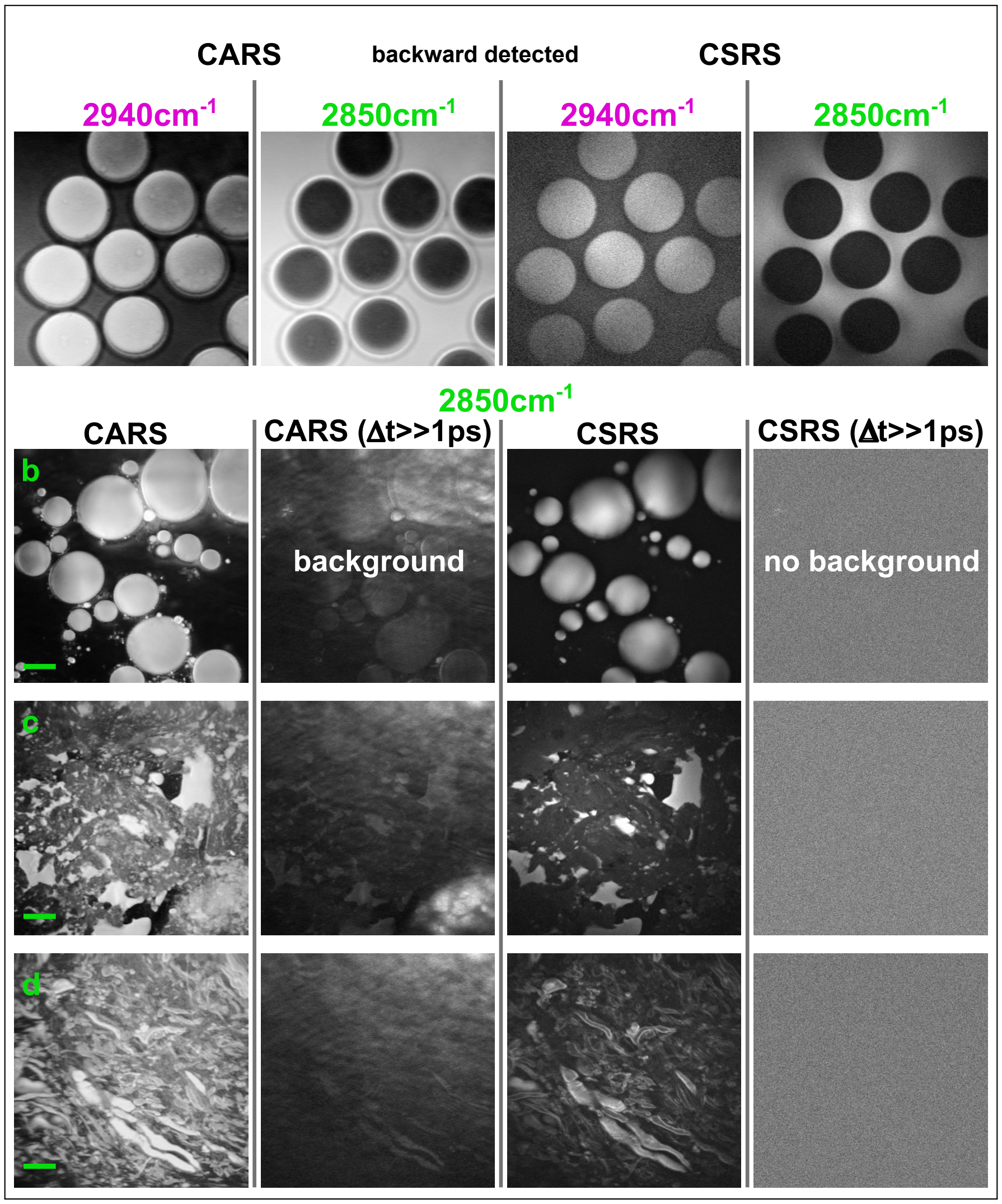


Fig. S1. Raw data from figure 4 and fluorescence contribution within the CARS channel when the temporal pulse overlap (Δt»1ps) is removed. (a) 20 µm PMMA beads dispersed in sunflower oil. (b–d) Avocado flesh, human fatty liver tissue, and myelin sheaths in sheep brain tissue, all imaged at 2850 $cm^{-1}$. All scale bars represent 10 µm. Effective pixel dwell time: 20 µs. Image acquisition time: 21 s for a 1024 × 1024-pixel image.

### 1.3. Image acquisition and data analysis

All imaging experiments were performed using ScanImage® (free version) [5] operated within the MATLAB environment and integrated with custom-developed, home-built control and acquisition scripts written in MATLAB. This configuration enabled flexible synchronization of laser modulation, signal detection, and data acquisition tailored to the coherent Raman imaging modalities employed in this work.

Both biological samples and PMMA bead samples were imaged using identical acquisition parameters to ensure consistency across measurements. Images were acquired with a pixel dwell time of 1 $\mu$s, a frame size of $1024 \times 1024$ pixels, and a field of view of $\sim 72\ \mu$m. The images shown in Fig. 4a–d are averages of 20 frames, corresponding to a cumulative integration time of 20 $\mu$s per pixel. The laser repetition rate was 0.7 MHz ($\sim 1.43\ \mu$s between pulses), i.e. $\sim 0.7$ pulses per pixel per frame ($\approx 14$ pulses per pixel in the 20-frame average). Boxcar integration was performed over a single pulse (no additional pulse averaging). Adjacent pixels are therefore not fully independent along the fast-scan direction, which slightly reduces the effective spatial resolution.

### 1.4. Raw data and fluorescence contribution

Within Fig. S1, we present the auto-contrasted raw images corresponding to the processed data shown in Fig. 4. The images in Fig. 4 were flat-field corrected and Fourier-filtered, with up to two-thirds of the Fourier support removed. In contrast, Fig. S1 displays the unprocessed data and therefore serves as a reference to assess fluorescence contributions in the CARS and CSRS detection channels. Notably, while a fluorescence background is evident in the CARS channel when the temporal pulse overlap is removed, no detectable fluorescence contribution is observed in the CSRS channel at 1470 nm.

## 2. CSRS in silico

### 2.1. Numerical implementation theory

The theoretical framework used in our numerical implementation is well established and has been presented in detail in the literature, including standard formulations of high-NA focusing (Debye–Wolf theory) and Green-function-based descriptions of coherent nonlinear signal generation [6–8]. For completeness and to keep this manuscript self-contained, we summarize the key equations and conventions here, so that the reader does not need to reconstruct the full formalism from multiple sources.

**Coordinate system and variables.** We place the origin at the nominal focus of the objective and use Cartesian coordinates $\boldsymbol{r} = (x, y, z)$ with optical axis $+z$. Angles on the reference sphere of the objective are denoted by $(\theta, \phi)$: $\theta \in [0, \theta_{\max}]$ is the polar angle from $+z$ and $\phi \in [0, 2\pi)$ the azimuth. The maximum polar angle is set by the numerical aperture in the sample medium of refractive index $n_2$,

$$\theta_{\max} = \arcsin\left(\frac{\mathrm{NA}}{n_2}\right). \tag{1}$$

We write $f$ for the objective focal length and $k = 2\pi n_2/\lambda_0$ for the wavenumber in the sample ($\lambda_0$ is the vacuum wavelength). The immersion/entrance medium has refractive index $n_1$.

**Focused field via the angular spectrum (Debye–Wolf).** The vectorial focal field is computed from the angular spectrum representation

$$\boldsymbol{E}_f(x,y,z) = \frac{ikf\,e^{ikf}}{2\pi} \int_0^{2\pi}\int_0^{\theta_{\max}} \boldsymbol{E}_{\rm far}(\theta,\phi)\; e^{ik\,[\,x\sin\theta\cos\phi + y\sin\theta\sin\phi + z\cos\theta\,]}\;\sin\theta\,d\theta\,d\phi. \tag{2}$$

Here $\boldsymbol{E}_{\rm far}(\theta,\phi)$ is the field on the reference sphere that already accounts for the polarization rotation by the lens and the aplanatic energy-conserving factor.

**Polarization mapping through the objective.** We model the lens action as a rotation from the entrance pupil polarization to the local tangent plane on the reference sphere, together with the aplanatic apodization factor $\sqrt{\cos\theta}$ and the refractive-index ratio $n_1/n_2$:

$$\boldsymbol{E}_{\rm far}(\theta,\phi) = \frac{n_1}{n_2}\,\sqrt{\cos\theta}\;\mathbf{M}_{\rm lens}(\theta,\phi)\;\boldsymbol{E}_{\rm inc}, \tag{3}$$

with the explicit Richards–Wolf lens block

$$\mathbf{M}_{\rm lens}(\theta,\phi) = \begin{pmatrix} \cos^2\phi\,\cos\theta + \sin^2\phi & \sin\phi\cos\phi\,(\cos\theta-1) & \cos\phi\,\sin\theta \\ \sin\phi\cos\phi\,(\cos\theta-1) & \cos^2\phi + \sin^2\phi\,\cos\theta & \sin\phi\,\sin\theta \\ -\cos\phi\,\sin\theta & -\sin\phi\,\sin\theta & \cos\theta \end{pmatrix}. \tag{4}$$

Equation (3) assumes an aplanatic, aberration-free high-NA objective; deviations (e.g. spherical aberration or index mismatch) can be incorporated via phase and amplitude factors multiplying $\boldsymbol{E}_{\rm far}$.

**Incident polarization model (uniform and vector beams).** To cover standard uniform Jones states (linear/elliptical/circular) and spatially varying vector beams (radial/azimuthal), we parameterize the entrance pupil field as [9]

$$\boldsymbol{E}_{\rm inc}(\alpha,\delta,\eta,\gamma;\phi) = \cos\eta \begin{pmatrix} \cos\alpha \\ e^{i\delta}\sin\alpha \\ 0 \end{pmatrix} + \sin\eta\left[\cos\gamma\begin{pmatrix}\cos\phi\\ \sin\phi\\ 0\end{pmatrix} + \sin\gamma\begin{pmatrix}-\sin\phi\\ \cos\phi\\ 0\end{pmatrix}\right]. \tag{5}$$

Here, $\alpha$ sets the $x/y$ amplitude ratio of the uniform Jones vector, $\delta$ is the $x$–$y$ relative phase (linear: $\delta = 0,\pi$; circular: $\alpha = \pi/4$, $\delta = \pm\pi/2$; elliptical: general $\alpha,\delta$). The mixing angle $\eta$ blends uniform polarization ($\eta = 0$) with spatially varying vector beams ($\eta = \pi/2$). The angle $\gamma$ selects the vector-beam type: radial ($\gamma = 0$), azimuthal ($\gamma = \pi/2$), or any hybrid in between. The azimuth $\phi$ is the pupil-plane angle; it appears only in the vector-beam part.

**Nonlinear source term.** The third-order nonlinear polarization at the coherent Stokes frequency (cS) is obtained by multiplying the susceptibility tensor $\chi^{(3)}_{cS}$ with the local electric field vectors. In compact tensor notation,

$$\mathbf{P}^{(3)}_{cS}(\mathbf{r}) = \chi^{(3)}_{cS} \otimes \mathbf{E}_S(\mathbf{r}) \otimes \mathbf{E}^*_p(\mathbf{r}) \otimes \mathbf{E}_S(\mathbf{r}), \tag{6}$$

while in component form (using Einstein summation convention),

$$P^{(3)}_{cS,a}(\mathbf{r}) = \chi^{(3)}_{cS,abcd}(\mathbf{r})\,E_{S,b}(\mathbf{r})\,E^*_{p,c}(\mathbf{r})\,E_{S,d}(\mathbf{r}). \tag{7}$$

Here the index $a$ denotes the polarization component of the generated field, whereas $b$, $c$, and $d$ run over the components of the driving fields. Each repeated index implies summation. In general, $\chi^{(3)}_{cS,abcd}$ is a fourth-rank tensor with 81 elements.

For simplicity, we consider here only isotropic samples, reducing the 81 tensor elements to 21 that are nonzero [8,10]. In this case the CSRS signal vanishes unless only two distinct polarization states are present among the interacting fields. That is, only elements of the type $\chi^{(3)}_{aaaa}$, $\chi^{(3)}_{aabb}$, and $\chi^{(3)}_{abab}$ (with $a$ and $b$ denoting $x$, $y$, or $z$) remain. Furthermore, these components obey the relation

$$\chi^{(3)}_{aaaa} = \chi^{(3)}_{aabb} + \chi^{(3)}_{abab} + \chi^{(3)}_{abba}, \tag{8}$$

as discussed in Ref. [8]. Within our simulations we chose $\chi^{(3)}_{aabb} = \chi^{(3)}_{abab} = \chi^{(3)}_{abba} = 1$, which according to Eq. (8) yields $\chi^{(3)}_{aaaa} = 3$. In reality, the symmetry of the Raman-active vibration defines the relative ratio of these tensor elements, often expressed through the depolarization ratio. Our choice of equal values therefore represents a practical simplification for the numerical implementation rather than the description of a specific vibrational mode.

**Wave equation.** In SI units, the nonlinear polarization $\mathbf{P}^{(3)}_{cS}$ acts as a source term in the inhomogeneous Helmholtz equation,

$$\nabla \times \nabla \times \mathbf{E}_{cS}(\mathbf{r}) - k^2_{cS}\, \mathbf{E}_{cS}(\mathbf{r}) = \mu_0 \omega^2_{cS}\, \mathbf{P}^{(3)}_{cS}(\mathbf{r}) = \frac{\omega^2_{cS}}{\varepsilon_0 c^2}\, \mathbf{P}^{(3)}_{cS}(\mathbf{r}). \tag{9}$$

Here, $k_{cS} = n_{cS}\omega_{cS}/c$ is the wavenumber of the coherent Stokes field in a medium of refractive index $n_{cS}$.

**Green function solution.** Using the outgoing scalar Green function with SI normalization, Eq. (9) gives the radiation-zone dyadic Green-function expression [7],

$$\mathbf{E}_{cS}(\mathbf{R}) = -\frac{\omega^2_{cS}}{\varepsilon_0 c^2} \iiint_V dV\, \frac{e^{ik_{cS}\,|\mathbf{R}-\mathbf{r}|}}{4\pi|\mathbf{R}-\mathbf{r}|}\, \hat{M}_{\mathrm{rad}}\big(\mathbf{R}-\mathbf{r}\big)\, \mathbf{P}^{(3)}_{cS}(\mathbf{r}), \tag{10}$$

where $\hat{M}_{\mathrm{rad}}(\mathbf{R}-\mathbf{r}) = \mathbf{I} - \hat{\mathbf{u}}\hat{\mathbf{u}}^{\mathsf{T}}$ is the transverse projector with $\hat{\mathbf{u}} = (\mathbf{R}-\mathbf{r})/|\mathbf{R}-\mathbf{r}|$ (i.e. it removes the component along the local radiation direction). This expression states that the detected field is a coherent superposition of contributions from the nonlinear polarization in the focal volume, each propagated with the scalar Green factor (with complex amplitude) and projected onto the plane transverse to the local propagation direction.

**Spherical components under far-field approximation.** To obtain a compact expression for the detected field we expand the phase factor in the far-field limit $R \gg |\mathbf{r}|$,

$$|\mathbf{R}-\mathbf{r}| \approx R - \frac{\mathbf{R}\cdot\mathbf{r}}{R}, \qquad \frac{1}{|\mathbf{R}-\mathbf{r}|} \approx \frac{1}{R}, \qquad e^{ik_{cS}|\mathbf{R}-\mathbf{r}|} \approx e^{ik_{cS}R}\, e^{-ik_{cS}\,(\mathbf{R}\cdot\mathbf{r})/R}. \tag{11}$$

In the far-field approximation the local propagation vector $\hat{\mathbf{u}}$ reduces to the global radial unit vector

$$\hat{\mathbf{e}}_R = (\sin\Theta\cos\Phi,\ \sin\Theta\sin\Phi,\ \cos\Theta),$$

with associated transverse unit vectors

$$\hat{\mathbf{e}}_\Theta = (\cos\Theta\cos\Phi,\ \cos\Theta\sin\Phi,\ -\sin\Theta), \qquad \hat{\mathbf{e}}_\Phi = (-\sin\Phi,\ \cos\Phi,\ 0).$$

With $\hat{\mathbf{e}}_R$ defined, the transverse projector can be written explicitly as

$$\hat{M}_{\rm rad} = \mathbf{I} - \hat{\mathbf{u}}\hat{\mathbf{u}}^{\mathsf{T}} \approx \mathbf{I} - \hat{\mathbf{e}}_R \hat{\mathbf{e}}_R^{\mathsf{T}} = \begin{pmatrix} 1 - \sin^2\Theta \, \cos^2\Phi & -\sin^2\Theta \, \cos\Phi \, \sin\Phi & -\sin\Theta \, \cos\Theta \, \cos\Phi \\ -\sin^2\Theta \, \cos\Phi \, \sin\Phi & 1 - \sin^2\Theta \, \sin^2\Phi & -\sin\Theta \, \cos\Theta \, \sin\Phi \\ -\sin\Theta \, \cos\Theta \, \cos\Phi & -\sin\Theta \, \cos\Theta \, \sin\Phi & 1 - \cos^2\Theta \end{pmatrix}. \tag{12}$$

The transformation matrix from Cartesian to spherical basis is

$$S(\Theta, \Phi) = \begin{pmatrix} \hat{\mathbf{e}}_R^{\mathsf{T}} \\ \hat{\mathbf{e}}_\Theta^{\mathsf{T}} \\ \hat{\mathbf{e}}_\Phi^{\mathsf{T}} \end{pmatrix}. \tag{13}$$

Applying the transformation matrix $S(\Theta, \Phi)$ to the projector form in Eq. (10) gives

$$\begin{pmatrix} E_R \\ E_\Theta \\ E_\Phi \end{pmatrix} = S(\Theta, \Phi) \, \hat{M}_{\rm rad}(\Theta, \Phi) \, \mathbf{P}^{(3)}_{cS}.$$

Because $S$ already contains only transverse rows for $E_\Theta$ and $E_\Phi$, the projector $\hat{M}_{\rm rad}$ acts trivially and can be omitted. Thus the relevant $2 \times 3$ block that maps the source polarization into measurable far-field components is

$$\mathbf{M}_{\rm ang}(\Theta, \Phi) = \begin{pmatrix} \cos\Theta \cos\Phi & \cos\Theta \sin\Phi & -\sin\Theta \\ -\sin\Phi & \cos\Phi & 0 \end{pmatrix}. \tag{14}$$

Finally, the detected transverse field amplitudes become

$$\begin{bmatrix} E_{cS,\Theta}(\mathbf{R}) \\ E_{cS,\Phi}(\mathbf{R}) \end{bmatrix} = -\frac{\omega_{cS}^2}{\varepsilon_0 c^2} \frac{e^{ik_{cS}R}}{4\pi R} \iiint_V dV \; e^{-ik_{cS} \, (\mathbf{R}\cdot\mathbf{r})/R} \, \mathbf{M}_{\rm ang}(\Theta, \Phi) \, \mathbf{P}^{(3)}_{cS}(\mathbf{r}). \tag{15}$$

This form makes explicit that only the two transverse spherical polarizations ($\Theta$ and $\Phi$) survive in the radiation zone, while the radial component vanishes due to transversality.

**Detected power (objective with cone angle $\alpha_{\rm max}$).** The time-averaged intensity of the coherent Stokes field is

$$I(R, \Theta, \Phi) \; = \; \frac{1}{2} \, \varepsilon_0 \, c \, n \, \left| \mathbf{E}_{cS}(R, \Theta, \Phi) \right|^2. \tag{16}$$

The power collected by an objective with maximum cone angle $\alpha_{\rm max}$ is then

$$P \; = \; \frac{1}{2} \, \varepsilon_0 \, c \, n \, R^2 \int_0^{\alpha_{\rm max}} \int_0^{2\pi} \left| \mathbf{E}_{cS}(R, \Theta, \Phi) \right|^2 \; \sin\Theta \, d\Phi \, d\Theta. \tag{17}$$

Here $n$ is the refractive index of the detection medium, $R$ the observation distance, and $(\Theta, \Phi)$ the polar and azimuthal angles in the detection solid angle.

**Vectorial refocusing of the backward-emitted field.** While Eq. (17) gives the total power emitted into a given collection cone, fiber or spectrometer coupling is governed by the complex vectorial CSRS field formed after refocusing, not only by the angularly integrated intensity. We therefore treat the computed backward CSRS far field as a coherent vectorial angular spectrum and propagate it through an ideal aplanatic imaging system using the Debye–Wolf formalism [6,11,12]. This procedure is the standard vectorial-imaging step used to map a collected far-field or reflected angular spectrum through an objective–tube-lens relay back into an image plane; here it is applied to the backward-emitted CSRS angular spectrum. The calculation determines the image-plane field structure and does not assume a particular guided fiber mode.

For backward collection, we introduce the object-side polar angle

$$\beta = \pi - \Theta, \tag{18}$$

such that $\beta = 0$ corresponds to emission along the backward optical axis $-z$. This is only a change of angular coordinates. The backward-emitted far field obtained from Eq. (15) is then written as a vectorial radiation amplitude on the object-side reference sphere by removing the common spherical propagation factor,

$$\mathcal{E}_{cS,\beta}(\beta,\Phi) = -\,R\,e^{-ik_{cS}R}\,E_{cS,\Theta}(R,\pi{-}\beta,\Phi), \qquad \mathcal{E}_{cS,\Phi}(\beta,\Phi) = R\,e^{-ik_{cS}R}\,E_{cS,\Phi}(R,\pi{-}\beta,\Phi). \tag{19}$$

The minus sign in the first expression accounts for the reversal of the optical axis: the polar unit vector associated with $\beta$ is opposite to $\hat{\mathbf{e}}_\Theta$ at $\Theta = \pi - \beta$, whereas the azimuthal unit vector is unchanged. The quantities $\mathcal{E}_{cS,\beta}$ and $\mathcal{E}_{cS,\Phi}$ are therefore not real-space fields yet, but the two complex transverse components of the backward-CSRS angular spectrum. Global phases introduced by the relay can be absorbed into the overall normalization; any polarization-dependent phase would be included in the complex pupil transmission defined below.

The experimentally relevant refocused CSRS field is not obtained by focusing this angular spectrum back into the original diffraction-limited source volume. Instead, the objective and tube lens form a magnified image of the emitting focal volume. For an aplanatic imaging system with lateral magnification $M$, the object-side collection angle $\beta$ and the image-side convergence angle $\gamma$ obey the Abbe sine condition,

$$n_\mathrm{o}\sin\beta = |M|\,n_\mathrm{i}\sin\gamma, \tag{20}$$

where $n_\mathrm{o}$ and $n_\mathrm{i}$ are the refractive indices on the object and image sides, respectively. Thus the numerical aperture at the refocused image plane is

$$\mathrm{NA_i} = n_\mathrm{i}\sin\alpha_\mathrm{i} = \frac{n_\mathrm{o}\sin\alpha_\mathrm{o}}{|M|} = \frac{\mathrm{NA_o}}{|M|}. \tag{21}$$

The magnification therefore scales the transverse wave-vector component, or equivalently the numerical aperture, rather than the polar angle itself except in the paraxial limit.

Each collected object-side ray is mapped to a corresponding image-side ray. We write the image-side angular spectrum as

$$\widetilde{\mathcal{E}}_{cS,\gamma}(\gamma,\Phi) = A_M(\beta)\,\mathcal{E}_{cS,\beta}(\beta,\Phi), \qquad \widetilde{\mathcal{E}}_{cS,\Phi}(\gamma,\Phi) = A_M(\beta)\,\mathcal{E}_{cS,\Phi}(\beta,\Phi), \tag{22}$$

with $\beta = \beta(\gamma)$ given by Eq. (20). Equation (22) preserves the two local transverse polarization components under an ideal aplanatic relay. The corresponding polarization rotation is implicit in the change of basis from the object-side vectors $\hat{\mathbf{e}}_\beta^{(o)}, \hat{\mathbf{e}}_\Phi^{(o)}$ to the image-side vectors $\hat{\mathbf{e}}_\gamma^{(i)}, \hat{\mathbf{e}}_\Phi^{(i)}$ used in Eq. (24). Equivalently, one could introduce an explicit rotation matrix acting on Cartesian field components, $\mathbf{Q}(\hat{\mathbf{k}}^{(i)}, \hat{\mathbf{k}}^{(o)})$ [14]; in the local meridional–azimuthal basis used here, this transformation does not mix the $\beta$- and $\Phi$-polarized amplitudes.

Here $A_M$ is the angular-amplitude mapping factor. It contains the complex pupil transmission $T_M(\beta)$, possible phase aberrations, and the Jacobian required when amplitudes defined on an object-side angular annulus are represented on the corresponding image-side angular annulus. For an ideal lossless system, conservation of power between corresponding angular annuli gives

$$A_M(\beta) = T_M(\beta) \left[ \frac{n_\mathrm{o} \sin\beta \, d\beta}{n_\mathrm{i} \sin\gamma \, d\gamma} \right]^{1/2} = T_M(\beta) \, |M| \left[ \frac{n_\mathrm{i} \cos\gamma}{n_\mathrm{o} \cos\beta} \right]^{1/2}. \tag{23}$$

This definition of $A_M$ includes the angular Jacobian and should therefore not be interpreted as the residual Richards–Wolf cosine apodization alone. That square-root cosine factor appears explicitly after the Debye integral is rewritten on the object-side angular grid below. In the absence of aberrations, $T_M(\beta)$ is set to one inside the transmitted pupil and zero outside. Constant prefactors and global phases can be absorbed into the overall normalization of the refocused field.

The refocused CSRS field in image-space coordinates $\mathbf{r}_i = (x_i, y_i, z_i)$ is then obtained from the image-side vectorial Debye integral,

$$\mathbf{E}_{cS}^{\mathrm{ref}}(\mathbf{r}_i) = C_i \int_0^{2\pi} \int_0^{\alpha_i} \left[ \widetilde{\mathcal{E}}_{cS,\gamma}(\gamma,\Phi) \, \hat{\mathbf{e}}_\gamma^{(i)}(\gamma,\Phi) + \widetilde{\mathcal{E}}_{cS,\Phi}(\gamma,\Phi) \, \hat{\mathbf{e}}_\Phi^{(i)}(\Phi) \right] e^{\,i k_i \hat{\mathbf{k}}^{(i)}(\gamma,\Phi)\cdot \mathbf{r}_i} \sin\gamma \, d\gamma \, d\Phi. \tag{24}$$

where $k_i = 2\pi n_\mathrm{i}/\lambda_{cS}$ and $C_i$ is an overall propagation constant. The image-side propagation direction and local transverse polarization basis are

$$\hat{\mathbf{k}}^{(i)}(\gamma,\Phi) = \begin{pmatrix} \sin\gamma\cos\Phi \\ \sin\gamma\sin\Phi \\ \cos\gamma \end{pmatrix}, \tag{25}$$

$$\hat{\mathbf{e}}_\gamma^{(i)}(\gamma,\Phi) = \begin{pmatrix} \cos\gamma\cos\Phi \\ \cos\gamma\sin\Phi \\ -\sin\gamma \end{pmatrix}, \qquad \hat{\mathbf{e}}_\Phi^{(i)}(\Phi) = \begin{pmatrix} -\sin\Phi \\ \cos\Phi \\ 0 \end{pmatrix}. \tag{26}$$

These vectors are the local transverse basis associated with each image-side plane-wave component. Thus the image-side Debye integral in Eq. (24) retains the two independent far-field polarization components and their relative phases while enforcing transversality ray by ray. The azimuthal component remains associated with the same $\hat{\mathbf{e}}_\Phi$ direction, whereas the meridional component is carried from the object-side $\hat{\mathbf{e}}_\beta^{(o)}$ direction into the image-side $\hat{\mathbf{e}}_\gamma^{(i)}$ direction. The longitudinal component of the refocused field is therefore determined by the image-side angle $\gamma$, not by the object-side angle $\beta$; for large magnification the refocused CSRS field is weakly focused and predominantly transverse.

For numerical implementation it is convenient to evaluate the same integral on the object-side angular grid. Differentiating the Abbe sine condition gives

$$\frac{d\gamma}{d\beta} = \frac{n_\mathrm{o}\cos\beta}{|M| \, n_\mathrm{i}\cos\gamma}. \tag{27}$$

Combining Eq. (23) with Eq. (27) yields the measure factor that enters the Debye integral,

$$A_M(\beta) \, \sin\gamma \, d\gamma = T_M(\beta) \, \sin\gamma(\beta) \left[ \frac{n_\mathrm{o}\cos\beta}{n_\mathrm{i}\cos\gamma(\beta)} \right]^{1/2} d\beta.$$

Thus Eq. (24) becomes, up to the same global normalization constant,

$$\begin{aligned}\mathbf{E}_{cS}^{\mathrm{ref}}(\mathbf{r}_i) = C_i \int_0^{2\pi} \int_0^{\alpha_o} & T_M(\beta)\, \sin\gamma(\beta) \left[\frac{n_{\mathrm{o}}\cos\beta}{n_{\mathrm{i}}\cos\gamma(\beta)}\right]^{1/2} \\ & \times \left[\mathcal{E}_{cS,\beta}(\beta,\Phi)\, \hat{\mathbf{e}}_{\gamma}^{(i)}(\gamma(\beta),\Phi) + \mathcal{E}_{cS,\Phi}(\beta,\Phi)\, \hat{\mathbf{e}}_{\Phi}^{(i)}(\Phi)\right] \\ & \times \exp\left[ik_i \hat{\mathbf{k}}^{(i)}(\gamma(\beta),\Phi)\cdot \mathbf{r}_i\right]\, d\beta\, d\Phi.\end{aligned} \tag{28}$$

This form makes explicit the familiar square-root cosine apodization that remains after the image-side Debye measure has been expressed in terms of the object-side angle $\beta$.

The component-resolved refocused CSRS intensities can then be evaluated as

$$I_{cS,x}^{\mathrm{ref}}(x_i, y_i; z_i) = \left|\hat{\mathbf{e}}_x \cdot \mathbf{E}_{cS}^{\mathrm{ref}}(x_i, y_i, z_i)\right|^2, \qquad I_{cS,y}^{\mathrm{ref}}(x_i, y_i; z_i) = \left|\hat{\mathbf{e}}_y \cdot \mathbf{E}_{cS}^{\mathrm{ref}}(x_i, y_i, z_i)\right|^2, \tag{29}$$

$$I_{cS,z}^{\mathrm{ref}}(x_i, y_i; z_i) = \left|\hat{\mathbf{e}}_z \cdot \mathbf{E}_{cS}^{\mathrm{ref}}(x_i, y_i, z_i)\right|^2, \qquad I_{cS,\mathrm{tot}}^{\mathrm{ref}} = I_{cS,x}^{\mathrm{ref}} + I_{cS,y}^{\mathrm{ref}} + I_{cS,z}^{\mathrm{ref}}. \tag{30}$$

These projections are only used to display the already transverse refocused field in fixed laboratory components; the polarization rotation and transversality are already accounted for in Eqs. (24) and (28). The resulting images describe the vectorial CSRS field available at the refocused image plane, for example at a fiber facet or spectrometer entrance plane.

#### 2.1.1. Excitation constraints

The relative efficiency of different pupil geometries depends on which excitation quantity is held fixed. We therefore compare three constraints, each applied independently to the pump and Stokes fields ($c \in \{p, S\}$), as summarized in Supplementary Fig. S6.

For equal input power, the optical power carried by the angular spectrum on the reference sphere is, up to a configuration-independent constant,

$$P_{\mathrm{in},c} \propto \int_0^{2\pi} \int_{\theta_{\min,c}}^{\theta_{\max,c}} \left|\mathbf{E}_{\mathrm{far},c}(\theta,\phi)\right|^2 \sin\theta\, \mathrm{d}\theta\, \mathrm{d}\phi. \tag{31}$$

Equation (3) gives $\mathbf{E}_{\mathrm{far},c} = (n_1/n_2)\sqrt{\cos\theta}\,\mathbf{M}_{\mathrm{lens}}\mathbf{E}_{\mathrm{inc},c}$. For the uniformly illuminated pupils used here, $|\mathbf{E}_{\mathrm{inc},c}| = 1$, and $\mathbf{M}_{\mathrm{lens}}$ is a rotation that preserves the field norm. Consequently, $|\mathbf{E}_{\mathrm{far},c}|^2 = (n_1/n_2)^2\cos\theta$, and Eq. (31) has the closed form

$$P_{\mathrm{in},c} \propto \pi\left(\sin^2\theta_{\max,c} - \sin^2\theta_{\min,c}\right). \tag{32}$$

For the uniformly illuminated aplanatic pupils considered here, Eq. (32) is therefore used to impose equal input power.

To describe spatially accumulated nonlinear exposure, we define the generalized field metric

$$D_{n,c} = \int_V |\mathbf{E}_c(\mathbf{r})|^{2n}\, \mathrm{d}V. \tag{33}$$

Configuration-independent intensity prefactors, as well as temporal factors such as pulse duration and repetition rate, cancel from the relative normalization. Multiphoton photodamage does not obey a universal power law: an effective exponent near 2.5 was reported for bovine adrenal chromaffin cells, while CARS measurements identified mixtures of linear and nonlinear damage mechanisms and a dependence on wavelength, repetition rate, and sample density [18, 19]. We therefore display the two bounding integer cases

$$D_{2,c} = \int_V |\mathbf{E}_c(\mathbf{r})|^4\, \mathrm{d}V, \tag{34}$$

$$D_{3,c} = \int_V |\mathbf{E}_c(\mathbf{r})|^6\, \mathrm{d}V. \tag{35}$$

An effective exponent near $n = 2.5$ is expected to produce relative efficiencies between these displayed cases. The main text uses $n = 2$ as the conservative lower-order nonlinear-exposure model for the high-pulse-energy regime, without implying that the same damage law applies to every specimen. Because wavelength-dependent absorption coefficients and mixed pump–Stokes terms are sample specific, pump and Stokes are normalized separately and cross terms are omitted.

Since the emitted CSRS power scales as $|E_p|^2|E_S|^4$, all three normalized maps follow from the same unnormalized calculation:

$$P_{\mathrm{CSRS}}^{(P_{\mathrm{in}})} \propto \frac{P_{\mathrm{CSRS}}^{(\mathrm{raw})}}{P_{\mathrm{in},p}P_{\mathrm{in},S}^2}, \quad P_{\mathrm{CSRS}}^{(D_2)} \propto \frac{P_{\mathrm{CSRS}}^{(\mathrm{raw})}}{D_{2,p}^{1/2}D_{2,S}}, \quad P_{\mathrm{CSRS}}^{(D_3)} \propto \frac{P_{\mathrm{CSRS}}^{(\mathrm{raw})}}{D_{3,p}^{1/3}D_{3,S}^{2/3}}. \tag{36}$$

Constant reference factors common to all pupil geometries cancel when each map is referenced to its maximum forward lin–lin CSRS signal. Supplementary Fig. S2 uses equal input power for the general CARS–CSRS wavelength comparison. Figure 2 and Supplementary Figs. S3 and S4 use the conservative $n = 2$ exposure constraint, while Supplementary Fig. S6 compares equal input power with the $n = 2$ and $n = 3$ exposure constraints.

### 2.2. Numerical results

We next present the numerical results for the CARS–CSRS wavelength scaling, the dependence of forward and backward CSRS on pupil geometry and polarization, and the vectorial structure of the backward field after refocusing.

**Numerical discretization.** The pupil-optimization calculations in Fig. 2 and Supplementary Figs. S3, S4, and S6 used a focal volume spanning $x, y \in [-2.8, 2.8]$ $\mu$m and $z \in [-7, 7]$ $\mu$m. The fields were sampled on a $31 \times 31 \times 38$ Cartesian grid, corresponding to spatial steps of approximately 0.19 $\mu$m laterally and 0.38 $\mu$m axially. The excitation angular spectra were sampled at nominal increments $\Delta\theta = 0.75°$ and $\Delta\phi = 3°$, and the radiation sphere at $\Delta\Theta = 3°$ and $\Delta\Phi = 6°$. Emission into $\Theta \leq 80°$ was classified as forward-directed and emission into $\Theta \geq 100°$ as backward-emitted. Tests with larger focal volumes, finer spatial grids, and finer angular sampling changed the relative emitted powers by less than 5%.

**Forward CARS and forward CSRS versus wavelength scaling.** Figure S2 shows the forward-emitted CARS and CSRS power as a function of Raman shift and numerical aperture when both excitation beams fill the back aperture and each color is normalized to equal input power according to Eqs. (31) and (32). The Stokes wavelength was fixed at 1030 nm, and the pump wavelength was adjusted to match the vibrational resonance. Across the CH-stretch region, the ratio $P_{\mathrm{CARS}}/P_{\mathrm{CSRS}}$ remains close to 10 and shows little dependence on NA. This difference is not introduced as a different molecular Raman cross section [13]. In electronically non-resonant samples, CARS and CSRS are governed by the same resonant Raman susceptibility and therefore contain equivalent vibrational information. The simulated power ratio instead reflects the different radiation wavelength and the finite spatial extent of the coherent Raman source. Interpreted through a wavelength-scaling relationship of the form $(\lambda_{cS}/\lambda_{aS})^n$, the simulations correspond to an effective exponent of $n \approx 2.8$, consistent with Mie-type scattering from a coherent Raman

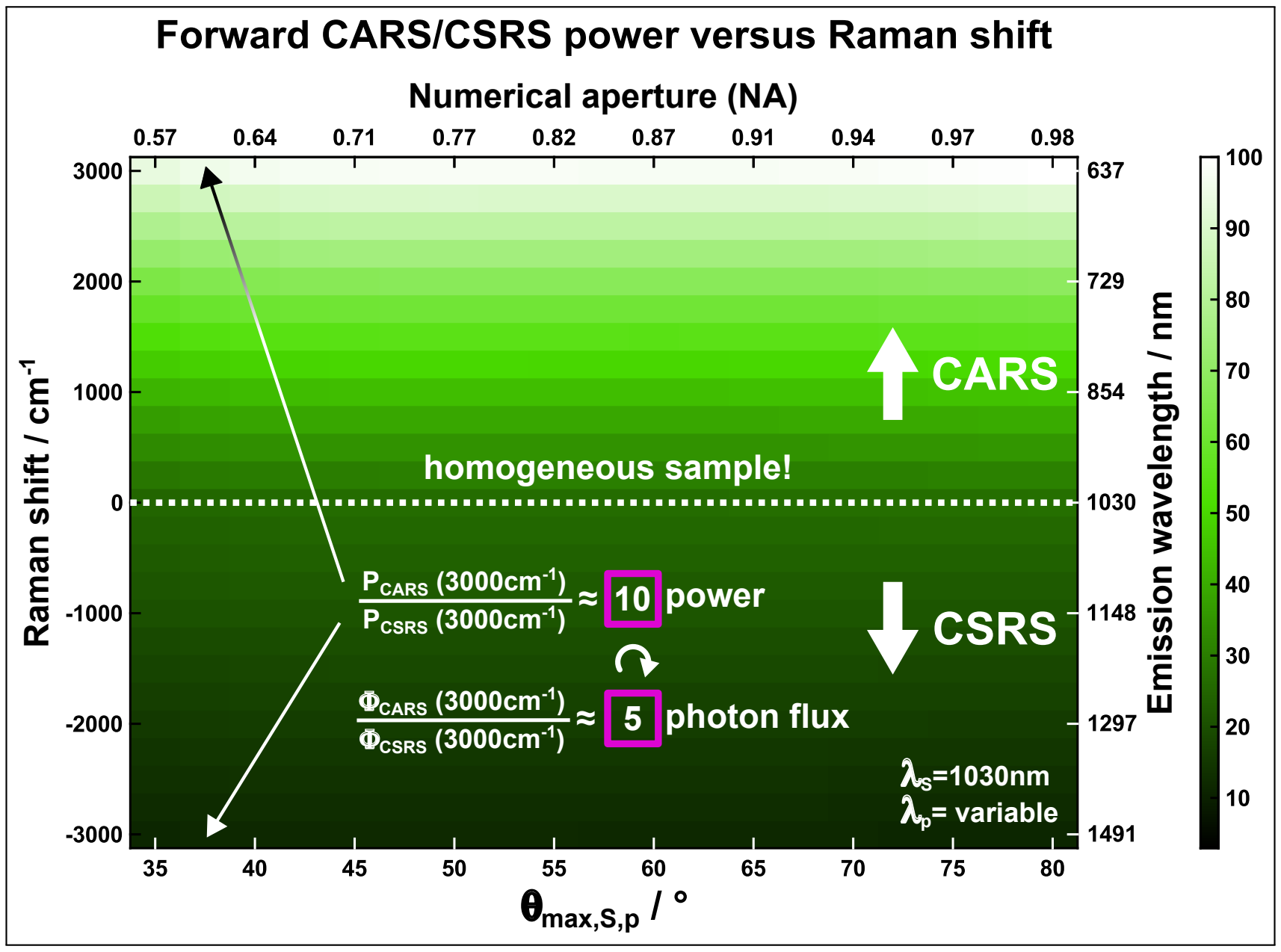


Fig. S2. Forward CARS versus forward CSRS radiation power as a function of Raman shift. For all investigated numerical apertures, the forward-emitted power at 3000 $cm^{-1}$ is roughly ten times higher for CARS ($\lambda_{aS}$ = 640 nm) than for CSRS ($\lambda_{cS}$ = 1490 nm). Owing to the reduced photon energy at the CSRS wavelength, this corresponds to an approximately fivefold lower CSRS photon flux, establishing the intrinsic wavelength-dependent efficiency difference between the two processes. Pump and Stokes both fill the back aperture and are independently normalized to equal input power according to Eq. (31).

source distribution with spatial extent similar to the optical wavelength. Accounting for the photon-energy difference (CARS photons carry $\sim 2.2\times$ more energy than CSRS photons), the forward photon-flux ratio is approximately 5.

A first general trend—visible in Fig. S2—is that the total forward-emitted CARS and CSRS powers depend only weakly on NA over the investigated range when the input powers are held fixed. Tighter focusing increases the local nonlinear polarization but simultaneously reduces the coherently contributing source volume and broadens the angular emission distribution; for the collected forward power these effects largely compensate.

**Forward and backward CSRS under controlled pupil illumination.** Having established the intrinsic CARS–CSRS wavelength dependence and its weak NA dependence under equal input power, we now examine how polarization and pupil filling govern *backward* CSRS generation. The ring-Stokes/central-pump survey in Fig. S3 and the dual-ring survey in Fig. S4 are evaluated using the same separate-color two-photon-load constraint as Fig. 2. The quantitative change produced by equal input power, equal two-photon load, and equal three-photon load is isolated for selected configurations in Supplementary Fig. S6. The $n = 3$ comparison progressively favors the high-intensity annular backward-CSRS geometries. Under the $n = 2$ constraint, $circ^{+}$–azi excitation reaches approximately one quarter of the maximum forward-CSRS power, while fully azimuthal excitation yields a larger total backward power but an annular refocused mode.

Backward CSRS shows a strong dependence on the polarization state of the excitation fields. As demonstrated in both figures, certain polarization combinations—such as opposite circular polarizations for pump and Stokes—suppress backward CSRS by up to two orders of magnitude,

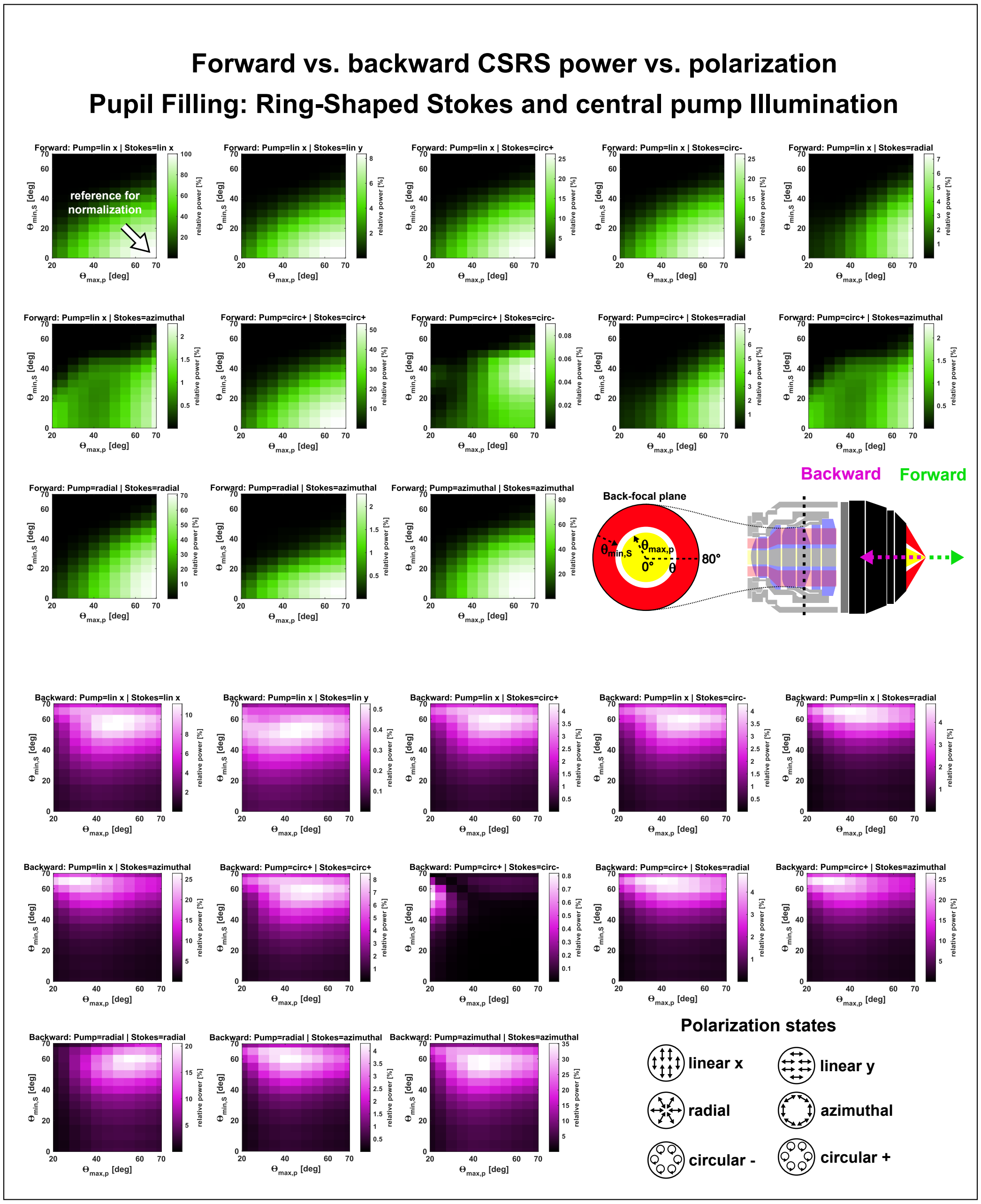


Fig. S3. Forward and backward CSRS emission for ring-shaped Stokes excitation with a central pump. Shown are two-dimensional maps of the simulated CSRS power as a function of the minimum and maximum excitation angles for pump and Stokes beams across thirteen representative polarization combinations. The plots illustrate how pupil filling and polarization jointly influence the efficiency of forward and backward CSRS generation, providing a visual guide to identify the angular–polarization conditions that yield the strongest signals. Pump and Stokes are independently normalized to equal volume-integrated two-photon excitation load, as defined by Eq. (34), and all values are referenced to the maximum forward lin–lin CSRS power obtained under the same constraint.

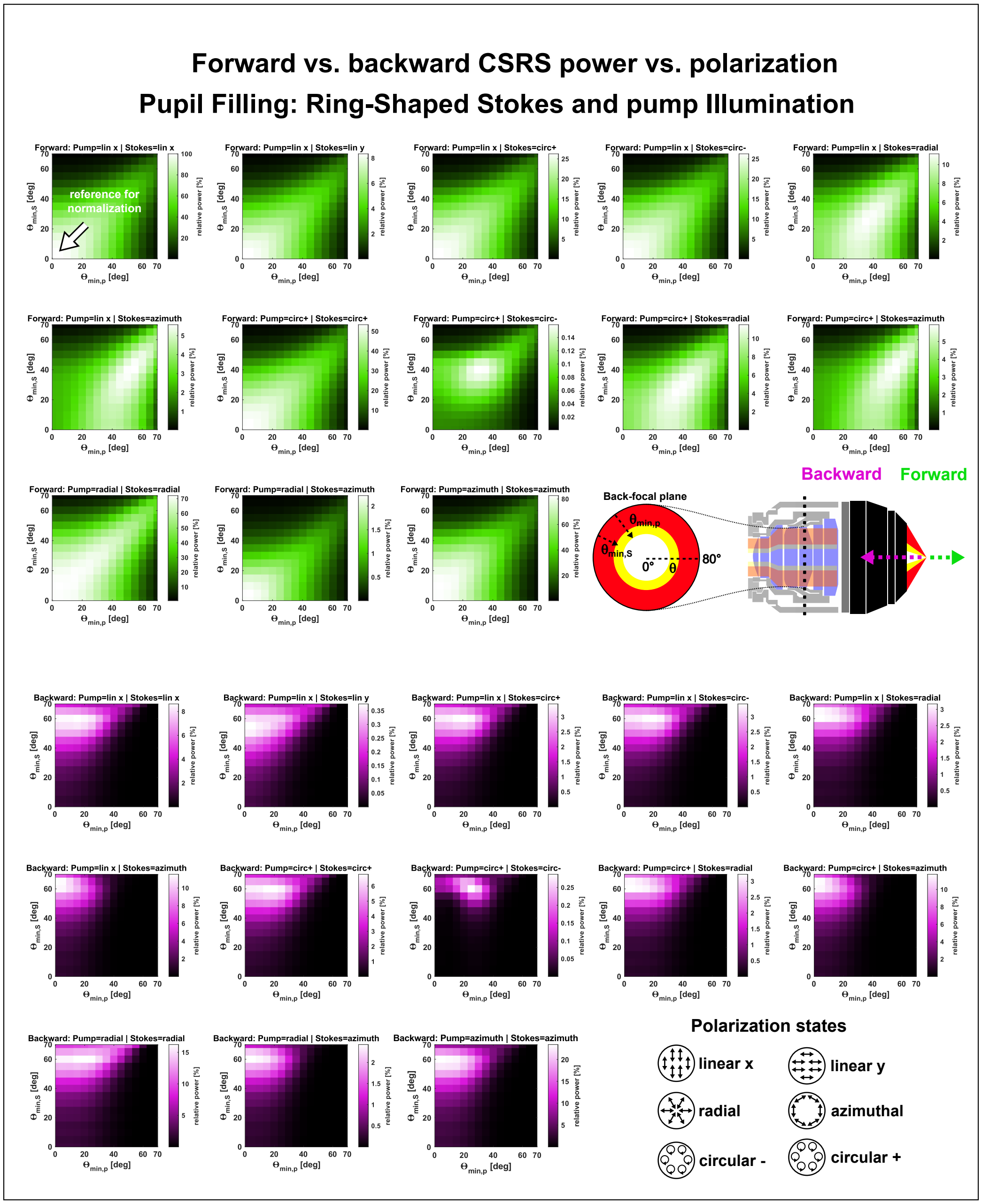


Fig. S4. Forward and backward CSRS emission for ring-shaped pump and Stokes illumination. Two-dimensional maps show the simulated CSRS power as a function of the minimum and maximum excitation angles when both pump and Stokes beams illuminate the pupil as rings, evaluated across thirteen representative polarization combinations. The figure assesses whether dual-ring excitation enhances backward CSRS efficiency—for example by favoring copropagating phase-matching conditions—but the results indicate no overall improvement compared to the ring-Stokes/central-pump geometry. Pump and Stokes are independently normalized to equal volume-integrated two-photon excitation load, as defined by Eq. (34), and all values are referenced to the maximum forward lin–lin CSRS power obtained under the same constraint.

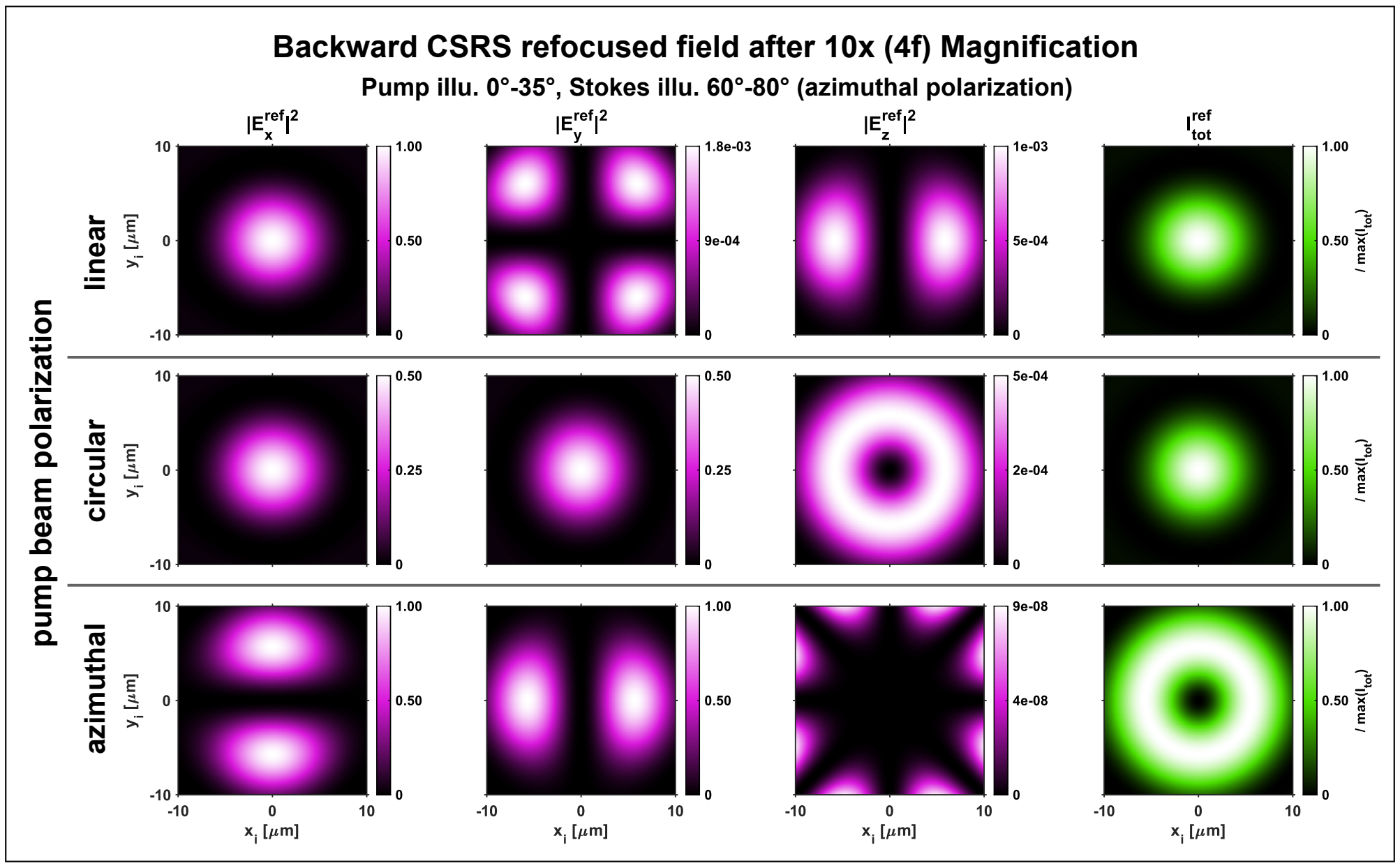


Fig. S5. Vectorial refocusing of the backward-emitted CSRS field. The computed backward CSRS angular spectrum was propagated to the image plane using Eq. (28). The Stokes illumination was azimuthally polarized and restricted to $\theta_S = 60°–80°$, while the pump illumination occupied $\theta_p = 0°–35°$ with linear, circular, or azimuthal polarization. The refocused field was calculated for a 10× objective–tube-lens magnification, corresponding to a reduced image-side numerical aperture according to Eq. (21). The columns show the component-resolved intensities $|E^{\mathrm{ref}}_{cS,x}|^2$, $|E^{\mathrm{ref}}_{cS,y}|^2$, $|E^{\mathrm{ref}}_{cS,z}|^2$, and the total intensity $I^{\mathrm{ref}}_{cS,\mathrm{tot}}$ in the image plane. Each image is normalized to its own maximum for visibility, while the color-bar labels indicate the corresponding amplitude relative to the maximum total intensity in the same row. For linear and circular pump excitation, the total refocused field is compact and single-lobed, whereas azimuthal pump excitation yields an annular transverse mode structure. In all cases, the longitudinal component remains strongly suppressed after magnified refocusing.

whereas azimuthally polarized Stokes light combined with a linearly polarized or circularly polarized pump produces the most efficient backward emission. This reflects the interplay between focal-field symmetry, excitation-field decomposition, and the anisotropic structure of the relevant $\chi^{(3)}$ tensor components.

To interpret these trends without overanalyzing individual pixel-level features, it is helpful to refer to the conceptual framework introduced in Fig. 2, where three representative phase-matching geometries—*counterpropagating*, *copropagating*, and *orthogonal* Stokes wavevector configurations—were illustrated. These geometries should be understood as intuitive categories rather than exact separations, but they provide a useful lens for interpreting how different excitation polarizations reshape the effective momentum balance.

For example, when the Stokes beam is azimuthally polarized and the pump is either circularly polarized (positive or negative) or linearly polarized along $x$, the simulations favor conditions reminiscent of the *counterpropagating* Stokes geometry. This manifests as a requirement for very high Stokes incidence angles, while the pump can remain at comparatively small polar angles.

In contrast, when both pump and Stokes beams are azimuthally or radially polarized, the angular conditions become considerably relaxed. In this situation, efficient backward CSRS emerges at substantially lower Stokes angles and larger pump angles, consistent with a stronger

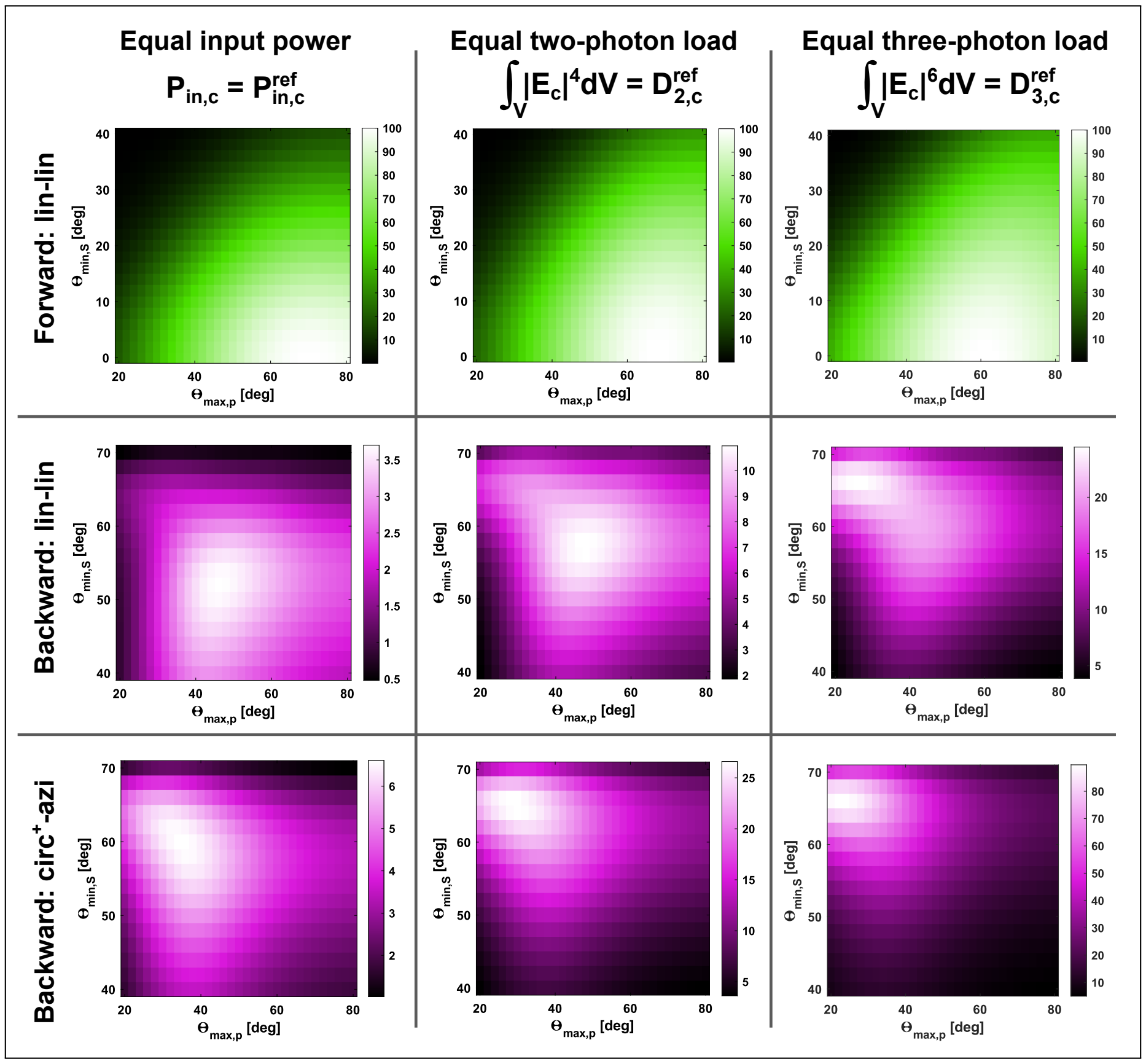


Fig. S6. Dependence of the simulated CSRS efficiency on the excitation constraint. Columns compare equal input power, $P_{\text{in},c}$, equal volume-integrated two-photon excitation load, $D_{2,c} = \int_V |\mathbf{E}_c|^4 \, dV$, and equal volume-integrated three-photon excitation load, $D_{3,c} = \int_V |\mathbf{E}_c|^6 \, dV$. Here, $c \in \{p, S\}$ denotes pump or Stokes, and each constraint is applied independently to the two colors. Rows show forward lin–lin, backward lin–lin, and backward circ$^+$–azi excitation. Numerical values denote emitted power relative to the maximum forward CSRS signal obtained under the corresponding constraint; color scales are adjusted independently for each panel. The $n = 2$ and $n = 3$ cases show how the relative efficiency evolves with the order of the nonlinear-exposure metric and bracket an effective exponent near $n = 2.5$. These metrics neglect mixed pump–Stokes absorption terms and wavelength-dependent damage coefficients and are therefore used as exposure proxies rather than as universal photodamage laws.

contribution from the *copropagating* or *orthogonal* representative geometries introduced in Fig. 2. These examples highlight the general pattern revealed by Fig. S3 and Fig. S4: the polarization state redistributes the excitation energy among the conceptual phase-matching families and thereby selects the angular regions that produce the strongest backward-directed emission.

**Vectorial structure after magnified refocusing.** To assess the field structure available for same-side detection, we applied the vectorial refocusing formalism of Eq. (28) to the computed backward CSRS angular spectrum. Figure S5 shows the image-plane field for the experimentally relevant Stokes geometry, namely azimuthally polarized Stokes illumination restricted to $\theta_S = 60°–80°$, combined with pump illumination over $\theta_p = 0°–35°$. We compare linear, circular, and azimuthal pump polarization and use a 10× magnified objective–tube-lens relay.

For linear and circular pump excitation, the refocused total intensity is compact and single-lobed, showing that the backward CSRS angular spectrum can be coherently refocused into a well-defined image-plane field. The component-resolved maps further show that the refocused field is predominantly transverse: the longitudinal component is suppressed by approximately three to four orders of magnitude relative to the row-wise maximum total intensity. This follows from the reduced image-side numerical aperture after magnification. For azimuthal pump excitation, the field remains well refocused but becomes annular, reflecting the different symmetry of the generated angular spectrum.

These calculations clarify the distinction between total backward-emitted power and experimentally useful refocused field structure. They show that backward CSRS is not only emitted into the collection cone, but can form a compact image-plane field suitable for practical same-side detection, including fiber- or spectrometer-based collection.

**Relative efficiencies and total photon budget.** The backward-to-forward CSRS ratio depends on the excitation constraint. Under the separate-color two-photon-load constraint used in Fig. 2, the experimentally selected circ$^+$–azi geometry reaches approximately 25% of the maximum forward-CSRS power, corresponding to a factor of about four. Under equal input power, the same geometry reaches only about 6%, whereas the three-photon-load constraint favors the high-intensity annular geometries more strongly and yields a substantially larger relative backward fraction. The displayed $n = 2$ and $n = 3$ results therefore bracket the expected trend for an effective damage exponent near $n = 2.5$, while the main text retains the more conservative $n = 2$ estimate. Combining the approximately fourfold backward-CSRS penalty obtained for this conservative nonlinear-exposure constraint with the approximately fivefold forward CARS–to–CSRS photon-flux ratio gives an overall reduction of roughly twenty relative to forward CARS. This number is therefore specific to the stated excitation constraint rather than an intrinsic universal efficiency ratio.

**In a nutshell.** Forward CARS is approximately an order of magnitude stronger in emitted power than forward CSRS, corresponding to an effective wavelength-scaling exponent of $n \approx 2.8$ and a photon-flux ratio of roughly five. The additional backward-CSRS penalty is constraint dependent: for the nonlinear-exposure constraint and circ$^+$–azi excitation used in Fig. 2, the total photon-flux reduction relative to forward CARS is approximately twenty. The optimum pupil geometry and achievable backward fraction therefore depend jointly on phase matching, polarization, and the laser–sample limitation that defines the admissible excitation.

## 3. Photon flux, noise and SNR

We measured the forward CARS and backward CSRS signal levels of sunflower oil to estimate the detected photon flux and benchmark the corresponding signal-to-noise ratio [15]. CARS was detected using a photomultiplier tube (PMT, Thorlabs PMT1001/M), while CSRS was detected using an InGaAs avalanche photodiode (APD, Thorlabs APD410C/M). Important parameters of

the SNR and photon flux estimation are summarized in Table S1. All photon fluxes reported below are inferred from the measured detector signals using the specified detector responsivity or noise-equivalent power and therefore inherently include the detector quantum efficiency.

### *Detected backward CSRS photon flux*

To determine the detected backward CSRS photon flux, we converted the measured APD output voltage $U$ into the corresponding optical power at the detector. With the APD410C transimpedance gain $G = 5 \times 10^5$ V/A (high-impedance readout), the measured $U = 2.5$ mV corresponds to

$$I_{\mathrm{ph}} = \frac{U}{G} = \frac{2.5 \times 10^{-3}}{5 \times 10^5} = 5.0 \times 10^{-9}\,\mathrm{A}. \tag{37}$$

Using the responsivity $R(1470\,\mathrm{nm}) \approx 18$ A/W at a multiplication factor $M = 20$, the detected optical signal power is

$$P_{\mathrm{sig}} = \frac{I_{\mathrm{ph}}}{R(1470\,\mathrm{nm})} \approx \frac{5.0 \times 10^{-9}}{18} \approx 2.8 \times 10^{-10}\,\mathrm{W}. \tag{38}$$

The photon energy at the CSRS wavelength $\lambda_{\mathrm{cS}} = 1470$ nm is

$$E_\gamma = \frac{hc}{\lambda_{\mathrm{cS}}} \approx 1.35 \times 10^{-19}\,\mathrm{J}. \tag{39}$$

The detected photon flux inferred from the APD signal is then

$$\Phi_{\mathrm{sig}} = \frac{P_{\mathrm{sig}}}{E_\gamma} \approx \frac{2.8 \times 10^{-10}}{1.35 \times 10^{-19}} \approx 2.1 \times 10^9\ \mathrm{photons/s}. \tag{40}$$

This value represents the photon flux at the detector inferred from the measured APD output. Due to transmission losses in the optical path, the photon flux emitted from the sample is approximately three times higher.

### *Noise and noise-equivalent photon flux*

The APD410C [16] exhibits an input-referred integrated noise-equivalent power $\mathrm{NEP_{int}} = 0.38\,\mathrm{nW_{RMS}}$ over a bandwidth of 10 MHz . The corresponding noise-equivalent detected photon flux is

$$\Phi_{\mathrm{NEP}} = \frac{\mathrm{NEP_{int}}}{E_\gamma} \approx \frac{0.38 \times 10^{-9}}{1.35 \times 10^{-19}} \approx 2.8 \times 10^9\ \mathrm{photons/s}, \tag{41}$$

which defines the baseline detector noise level prior to time gating.

### *Time-gated noise and detected SNR for a* $1\,\mu$s *integration*

The CSRS signal arrives in pulses at a repetition rate of $f_{\mathrm{rep}} = 0.707$ MHz ($T_{\mathrm{rep}} \approx 1.41\,\mu$s). The boxcar gate selects the temporal region containing the CSRS pulse and has an effective open width of $\tau_{\mathrm{gate}} \approx 45$ ns. For an integration interval of $\tau = 1\,\mu$s, this corresponds on average to

$$\tau_{\mathrm{open}} \approx 0.707 \times 45\,\mathrm{ns} \approx 32\,\mathrm{ns},$$

so that only this fraction of the detector noise contributes to the measurement. The reduction of the effective noise-integration time from $T_{\mathrm{rep}}$ to $\tau_{\mathrm{gate}}$ lowers the noise standard deviation by approximately a factor of six [17].

Using the noise spectral density $\mathrm{NEP}_\nu$ (derived from $\mathrm{NEP}_{\mathrm{int}}$ above), the RMS fluctuation of the time-integrated noise power over the open-gate interval is

$$\sigma_P(\tau_{\mathrm{open}}) = \frac{\mathrm{NEP}_\nu}{\sqrt{2\,\tau_{\mathrm{open}}}} \approx 4.8 \times 10^{-10}\,\mathrm{W}. \tag{42}$$

The corresponding number of noise-equivalent detected photons within 1 $\mu$s is

$$N_{\mathrm{noise}}(1\,\mu\mathrm{s}) = \frac{\sigma_P(\tau_{\mathrm{open}})\,\tau_{\mathrm{open}}}{E_\gamma} \approx 1.1 \times 10^2. \tag{43}$$

The detected signal photon flux yields

$$N_{\mathrm{sig}}(1\,\mu\mathrm{s}) = \Phi_{\mathrm{sig}}\,\tau \approx 2.1 \times 10^3, \tag{44}$$

corresponding to approximately 0.71 CSRS pulses within the integration window. The resulting detected signal-to-noise ratio is

$$\mathrm{SNR}(1\,\mu\mathrm{s}) = \frac{N_{\mathrm{sig}}(1\,\mu\mathrm{s})}{N_{\mathrm{noise}}(1\,\mu\mathrm{s})} \approx 19. \tag{45}$$

This value quantifies the detected SNR of a single 1 $\mu$s time-gated measurement prior to any further averaging.

### *Detected forward CARS photon flux*

To determine the detected forward CARS photon flux, we converted the measured PMT output voltage $U$ into the corresponding optical power at the detector. The PMT1001/M [20] incorporates a transimpedance amplifier with $G_{\mathrm{TI}} \approx 1.1 \times 10^4\,\mathrm{V/A}$. For the measured CARS signal of $U = 0.12\,\mathrm{V}$, the corresponding anode current is

$$I_{\mathrm{an}}^{(\mathrm{CARS})} = \frac{U}{G_{\mathrm{TI}}} \approx 1.1 \times 10^{-5}\,\mathrm{A}. \tag{46}$$

The photocathode radiant sensitivity at the CARS wavelength $\lambda_{\mathrm{CARS}} = 650\,\mathrm{nm}$ is $S(650\,\mathrm{nm}) \approx 7.8 \times 10^{-2}\,\mathrm{A/W}$, and the PMT gain at the experimental control voltage $V_{\mathrm{control}} = 0.601\,\mathrm{V}$ is

$$G_{\mathrm{HV}}(0.601\,\mathrm{V}) \approx 1.3 \times 10^4.$$

The detected optical signal power is therefore

$$P_{\mathrm{sig}}^{(\mathrm{CARS})} = \frac{I_{\mathrm{an}}^{(\mathrm{CARS})}}{S(650\,\mathrm{nm})\,G_{\mathrm{HV}}(0.601\,\mathrm{V})} \approx \frac{1.1 \times 10^{-5}}{7.8 \times 10^{-2} \times 1.3 \times 10^4} \approx 1.1 \times 10^{-8}\,\mathrm{W}. \tag{47}$$

The photon energy at this wavelength is

$$E_\gamma^{(\mathrm{CARS})} = \frac{hc}{\lambda_{\mathrm{CARS}}} \approx 3.1 \times 10^{-19}\,\mathrm{J}. \tag{48}$$

The detected forward CARS photon flux is then

$$\Phi_{\mathrm{sig}}^{(\mathrm{CARS})} = \frac{P_{\mathrm{sig}}^{(\mathrm{CARS})}}{E_\gamma^{(\mathrm{CARS})}} \approx \frac{1.1 \times 10^{-8}}{3.1 \times 10^{-19}} \approx 3.5 \times 10^{10}\,\mathrm{photons/s}. \tag{49}$$

This value represents the photon flux inferred from the measured PMT signal and provides the basis for the subsequent noise and SNR analysis.

*Noise and noise-equivalent photon flux (PMT)*

For the PMT1001/M we estimate the noise-equivalent detected photon flux consistently with the forward CARS calibration above. The input-referred current-noise density of the integrated transimpedance amplifier is $i_n = 6.5\ \mathrm{pA}/\sqrt{\mathrm{Hz}}$ at 1 MHz (PMT1000 Series Manual, Tab. 10.3). For the selected amplifier bandwidth $B = 2.5$ MHz this corresponds to an RMS anode-current noise

$$i_{\mathrm{n,rms}} = i_n \sqrt{B} \approx 1.0 \times 10^{-8}\ \mathrm{A}.$$

To convert this current noise into an equivalent optical power at the detector, we use the photocathode radiant sensitivity at the CARS wavelength, $S(650\ \mathrm{nm}) \approx 7.8 \times 10^{-2}\ \mathrm{A/W}$, and the PMT electron gain at the operating high-voltage setting, $G_{\mathrm{HV}}(0.601\ \mathrm{V}) \approx 1.3 \times 10^4$. The noise-equivalent optical power is then

$$P_{\mathrm{NEP}} = \frac{i_{\mathrm{n,rms}}}{S(650\ \mathrm{nm})\, G_{\mathrm{HV}}} \approx 1.0 \times 10^{-11}\ \mathrm{W}.$$

so that the corresponding noise-equivalent detected photon flux is

$$\Phi_{\mathrm{NEP}} \approx 3.3 \times 10^7\ \mathrm{photons/s}.$$

**Noise and detected SNR for a 1 $\mu$s integration (PMT, no time gating)** For forward CARS detected with the PMT1001/M, the detected photon flux is $\Phi_{\mathrm{sig}}^{(\mathrm{CARS})} \approx 3.5 \times 10^{10}$ photons/s. The pixel dwell time in the laser-scanning microscope is $\tau = 1\ \mu$s, so that the number of detected photons per pixel is

$$N_{\mathrm{sig}}(1\ \mu\mathrm{s}) = \Phi_{\mathrm{sig}}^{(\mathrm{CARS})}\, \tau \approx 3.5 \times 10^4.$$

From the amplifier specifications above, the noise-equivalent detected photon flux is $\Phi_{\mathrm{NEP}} \approx 3.3 \times 10^7$ photons/s. Without temporal gating, the effective noise-integration time equals the pixel dwell time, yielding

$$N_{\mathrm{noise}}(1\ \mu\mathrm{s}) = \Phi_{\mathrm{NEP}}\, \tau \approx 3.3 \times 10^1.$$

The resulting electronics-limited detected signal-to-noise ratio (not experimentally observed) is therefore

$$\mathrm{SNR}_{\mathrm{el}}(1\ \mu\mathrm{s}) \approx 1.1 \times 10^3.$$

In contrast, the photon shot noise associated with the detected CARS signal is set by the number of detected photoelectrons. With a photocathode quantum efficiency of approximately 0.2, the number of detected photoelectrons in a 1 $\mu$s integration window is $N_e \approx 7.0 \times 10^3$, yielding a shot-noise-limited detected SNR of

$$\mathrm{SNR}_{\mathrm{shot}}(1\ \mu\mathrm{s}) \approx 8.4 \times 10^1.$$

Since $\mathrm{SNR}_{\mathrm{el}} \gg \mathrm{SNR}_{\mathrm{shot}}$, the combined detected SNR is set by photon shot noise, and the contribution of PMT electronics noise is negligible under our experimental conditions.

The following table summarizes important parameters and results. Comparing the photon fluxes emitted at the focal volume, the forward CARS signal exceeds the backward CSRS signal by approximately one order of magnitude (factor 5.6), in good agreement with the ratio predicted by our simulations (factor 20).

The experimentally inferred forward CARS photon flux is expected to be slightly underestimated, as the axicons and azimuthal polarization optics were removed for these measurements, leading to an underfilling of the objective pupil and a non-optimal overlap of the pump and Stokes point-spread functions.

Taking this systematic effect into account, the experimentally inferred photon fluxes emitted at the focus are consistent with the simulated scaling between forward CARS and backward CSRS, supporting the validity of the photon-flux estimation and the underlying model.

Table S1. Summary of optical, detector, and photon-flux parameters used for estimating photon budgets, noise-equivalent photon flux, and detected SNR for forward CARS and backward CSRS detection.

| **Parameter** | **Forward CARS (PMT1001/M)** [20] | **Backward CSRS (APD410C/M)** [16] |
|---|---|---|
| *Excitation* | | |
| Pump / Stokes wavelength (nm) | 796 / 1030 | 796 / 1030 |
| Pump / Stokes power (mW) | 2.5 / 1.5 | 1.2 / 3.1 |
| Repetition rate $f_{\mathrm{rep}}$ (MHz) | 0.707 | 0.707 |
| Pixel dwell time $\tau$ ($\mu$s) | 1 | 1 (time-gated) |
| *Optical transmission* | | |
| Detection wavelength $\lambda_{\mathrm{det}}$ (nm) | 650 | 1470 |
| Transmission focus $\rightarrow$ detector $T$ | 0.90 | 0.30 |
| *Detector* | | |
| Detector type | PMT | InGaAs APD |
| Measured detector output voltage $U$ | 0.12 V | 2.5 mV |
| Responsivity $R$ or sensitivity $S$ | $S(650) = 7.8 \times 10^{-2}$ A/W | $R(1470) \approx 18$ A/W @ $M = 20$ |
| Quantum efficiency (QE) | $\approx 0.20$ | $\approx 0.80$ |
| Internal gain | $G_{\mathrm{HV}} \approx 1.3 \times 10^4$ | $M \approx 20$ |
| Transimpedance $G_{\mathrm{TI}}$ (V/A) | $1.1 \times 10^4$ | $5 \times 10^5$ |
| Bandwidth $B$ (MHz) | 2.5 | 10 |
| Noise spec. (input-referred) | $i_n = 6.5$ pA/$\sqrt{\mathrm{Hz}}$ | $\mathrm{NEP_{int}} = 0.38$ $\mathrm{nW_{RMS}}$ |
| Time-gate width (ns) | – | $\approx 45$ |
| Effective open time in 1 $\mu$s (ns) | 1000 (no gating) | $\approx 32$ |
| *Photon flux* | | |
| Photon flux emitted at focus $\Phi_{\mathrm{focus}}$ (phot/s) | $3.9 \times 10^{10}$ | $7.0 \times 10^9$ |
| Photon flux before detector $\Phi_{\mathrm{pre-det}}$ (phot/s) | $3.5 \times 10^{10}$ | $2.1 \times 10^9$ |
| Detected photoelectron rate $\Phi_e$ ($e^-$/s) | $7.0 \times 10^9$ | $1.7 \times 10^9$ |
| *CARS / CSRS ratio (above rows)* | $\approx 5.6 / \approx 17 / \approx 4.1$ | *(focus / pre-det / detected)* |
| *Extracted quantities (1 $\mu$s integration)* | | |
| Detected signal electrons in 1 $\mu$s | $7.0 \times 10^3$ | $1.7 \times 10^3$ |
| Noise-equivalent photon flux $\Phi_{\mathrm{NEP}}$ (phot/s) | $3.3 \times 10^7$ | $1.1 \times 10^8$ (effective, gated) |
| Noise-equivalent electrons in 1 $\mu$s | $3.3 \times 10^1$ | $1.1 \times 10^2$ |
| Detected SNR for 1 $\mu$s | $\approx 8.4 \times 10^1$ (shot-noise limited) | $\approx 19$ |

**Experimental SNR benchmark against theory.** To compare the theoretically predicted SNR values (table S1) with measurements, we evaluate the SNR directly from experimental images acquired on a homogeneous reference sample (sunflower oil). Instead of repeating the same measurement in time, we exploit spatial redundancy by computing the SNR from the central $20 \times 20$ pixels of one CSRS image and one CARS image, assuming that this small central region ($\ll 10$ $\mu$m) contains no structural contrast and that the pixel-to-pixel fluctuations are therefore dominated by noise. We use the same definition as in the theoretical section, SNR = signal/std, i.e. the mean signal level divided by the standard deviation within the ROI.

For technical reasons, the pixel dwell time was set to 4 $\mu$s (instead of 1 $\mu$s used in the example calculation), because our laser repetition rate is on the order of 1 $\mu$s and shorter dwell times lead to additional sampling artifacts. To report an equivalent value for 1 $\mu$s, the measured SNR is divided by 2, assuming the standard SNR $\propto \sqrt{t}$ scaling with integration time. We find that the experimentally obtained SNR values are approximately a factor of two higher than the theoretical estimates within table S1, which may be explained by conservative (worst-case) noise

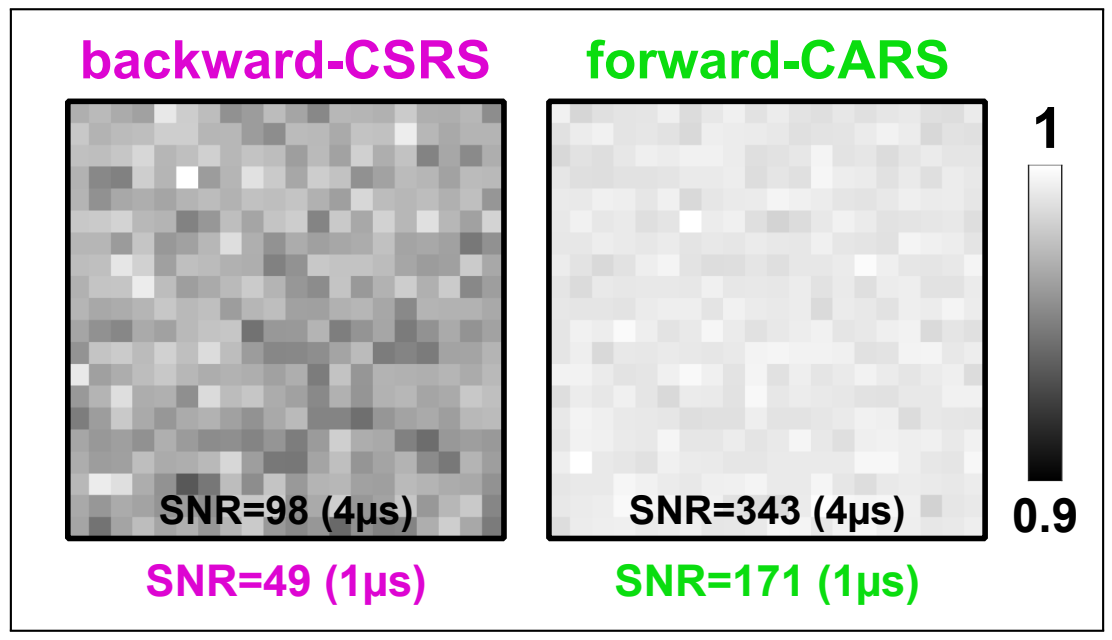


Fig. S7. SNR extracted from experimental backward-CSRS and forward-CARS images of sunflower oil at 2850 cm$^{-1}$ acquired with 4 $\mu$s pixel dwell time.

specifications of the detectors used in the model. For practical imaging and application scenarios, it should also be noted that the SNR decreases towards the field edges due to vignetting, such that the central ROI represents an upper bound of the achievable SNR across the full image.

## References for Supplementary Information


1. S. Heuke, X. Audier, and H. Rigneault, "Double-modulation stimulated raman scattering: how to image up to 16-fold faster," Opt. Lett. **48**, 423–426 (2023).
2. S. Heuke, F. B. Legesse, D. Akimov, U. Hübner, J. Dellith, M. Schmitt, and J. Popp, "Bessel beam coherent anti-stokes raman scattering microscopy," J. Opt. Soc. Am. B **32**, 1773–1779 (2015).
3. S. Heuke, J. Zheng, D. Akimov, R. Heintzmann, M. Schmitt, and J. Popp, "Bessel beam cars of axially structured samples," Sci. Reports **5**, 10991 (2015).
4. P. Fimpel, C. Riek, L. Ebner, A. Leitenstorfer, D. Brida, and A. Zumbusch, "Boxcar detection for high-frequency modulation in stimulated raman scattering microscopy," Appl. Phys. Lett. **112**, 161101 (2018).
5. T. A. Pologruto, B. L. Sabatini, and K. Svoboda, "Scanimage: Flexible software for operating laser scanning microscopes," Biomed. Eng. Online **2**, 13 (2003).
6. B. Richards and E. Wolf, "Electromagnetic diffraction in optical systems. ii. structure of the image field in an aplanatic system," Proc. Royal Soc. London. Ser. A **253**, 358–379 (1959).
7. J.-X. Cheng, A. Volkmer, and X. S. Xie, "Theoretical and experimental characterization of coherent anti-stokes raman scattering microscopy," J. Opt. Soc. Am. B **19**, 1363–1375 (2002).
8. J.-X. Cheng and X. S. Xie, eds., *Coherent Raman Scattering Microscopy (Series in Cellular and Clinical Imaging)* (CRC Press, 2016).
9. Q. Zhan, "Cylindrical vector beams: from mathematical concepts to applications," Adv. Opt. Photonics **1**, 1–57 (2009).
10. R. W. Boyd, *Nonlinear Optics* (Academic Press, 2020), 4th ed.
11. L. Novotny, R. D. Grober, and K. Karrai, "Reflected image of a strongly focused spot," Opt. Lett. **26**, 789–791 (2001).
12. P. Török, P. R. T. Munro, and E. E. Kriezis, "High numerical aperture vectorial imaging in coherent optical microscopes," Opt. Express **16**, 507–523 (2008).
13. K. Bito, M. Okuno, H. Kano, P. Leproux, V. Couderc, and H.-o. Hamaguchi, "Three-pulse multiplex coherent anti-stokes/stokes raman scattering (cars/csrs) microspectroscopy using a white-light laser source," Chem. Phys. **419**, 156–162 (2013).
14. S. Heuke, K. Unger, S. Khadir, K. Belkebir, P. C. Chaumet, H. Rigneault, and A. Sentenac, "Coherent anti-stokes raman fourier ptychography," Opt. Express **27**, 23497–23514 (2019).
15. B. E. A. Saleh and M. C. Teich, *Fundamentals of Photonics* (Wiley, 2007), 2nd ed.
16. Thorlabs, Inc., "Apd410c/m – ingaas variable-gain avalanche photodetector, 900–1700 nm, dc–10 mhz," (2015). Accessed: 2025-12-26.
17. X. Audier, S. Heuke, P. Volz, I. Rimke, and H. Rigneault, "Noise in stimulated raman scattering measurement: From basics to practice," APL Photonics **5**, 011101 (2020).
18. A. Hopt and E. Neher, "Highly nonlinear photodamage in two-photon fluorescence microscopy," Biophys. J. **80**, 2029–2036 (2001).
19. Y. Fu, H. Wang, R. Shi, and J.-X. Cheng, "Characterization of photodamage in coherent anti-Stokes Raman scattering microscopy," Opt. Express **14**, 3942–3951 (2006).
20. Thorlabs, Inc., "Pmt1001/m – multialkali amplified pmt, 230–920 nm (pmt1000 series) and amplifier noise specifications," (2016). Accessed: 2025-12-26.
