# Supplementary material for: Intrinsic backward emission of coherent Raman photons from homogeneous media
Source: Nat Photonics. 2026 Oct 19;20(11):1318–24. doi: 10.1038/s41566-026-01995-z (PMC13747816; doi:10.1038/s41566-026-01995-z)
Supplement: Supplementary file 2 [file 41566_2026_1995_MOESM2_ESM.pdf]

nature portfolio

Peer Review File

Intrinsic backward emission of coherent Raman photons from homogeneous media

Corresponding Author: Dr Sandro Heuke

This manuscript has been previously reviewed at another journal. This document only contains information relating to versions considered at Nature Photonics.

This file contains all reviewer reports in order by version, followed by all author rebuttals in order by version.

Version 0:

Reviewer comments:

Reviewer #1

(Remarks to the Author)
This manuscript demonstrates the backward emission of coherent Stokes Raman scattering (CSRS) from homogeneous media for epi-coherent microscopy. In previous coherent nonlinear microscopy techniques, such as second-harmonic generation (SHG), third-harmonic generation (THG), stimulated Raman scattering (SRS), and coherent anti-Stokes Raman scattering (CARS), epi-detection has generally relied either on nanoscopic inhomogeneous signal sources or on multiple scattering in thick samples. In the former case, the signal can be very weak, whereas in the latter case, the spatial coherence of the signal is lost. In the present method, the backward signal is generated by engineering the incident angle and polarization of the Stokes and pump beams in CSRS. Specifically, the Stokes beam has an annular profile and azimuthal polarization at the back aperture of the high-NA objective lens. As a result, owing to the phase-matching condition and dipole radiation from the nonlinear polarization, coherent backward radiation can be produced. An important limitation is that the signal is much weaker than in typical forward-detection CARS experiments. Nevertheless, the authors successfully demonstrate epi-detection CSRS imaging. Considering that this is the first demonstration of epi-detection of a coherent nonlinear signal, which has long been thought to be impossible, I recommend this manuscript for publication, subject to the following minor comments.

C1. I suggest that the authors further clarify the meaning of “counterpropagating” and “copropagating” in Fig. 2. It is not immediately clear that these terms refer to the ‘lateral components’ of the two interacting Stokes beams.

C2. Spatial resolution is an important performance metric in nonlinear microscopy. Nevertheless, the authors report only the lateral spatial resolution. I understand that it may be difficult to rigorously define the point spread functon(PSF) of CSRS. However, even a numerical analysis of the product of the electric field distributions, such as E_S^2(r)E_p(r), even at the scalar level, would provide a rough estimate of the spatial resolution. I expect that, owing to the annular Stokes beam, this method may achieve high lateral and axial resolution.

C3. The authors argue that the refocusable nature of the signal is advantageous because the field can be transmitted through an optical fiber. I agree that this is a potentially important advantage. However, I am not sure whether the epi-signal in the present CSRS scheme can actually be coupled into a fiber, given the complex electric field distribution of the epi-detected CSRS signal. In particular, when an azimuthally polarized beam is focused, the resulting focal field can have a complex electric field distribution that may not be compatible with the supported fiber mode. I suggest that the authors further investigate the electric field distribution of the CSRS signal and discuss this point in more detail.

Reviewer #2

(Remarks to the Author)
The authors implemented an excitation geometry optimized for the direct generation of backward-directed coherent Stokes Raman scattering (CSRS). This seemingly-straighforward implementation is something not explicitly considered in the literature. The combination of the theoretical analysis presented and the experimental demonstration of biological imaging is

convincing. This technique would add to the growing toolbox of nonlinear Raman scattering microscopy.

In the rapid growing field of nonlinear Raman microscopy, while SRS (stimulated Raman scattering) works primarily in the forward direction, CARS (Coherent Anti-Stokes Raman Scattering) has also been occasionally used for background-directed for sub-micron sized objects. However, no one in the Raman imaging field had considered fundamental difference between CSRS and CARS in terms of phase matching condition in the background direction, and simply assumed no particular advantage by CSRS. As pointed out by the authors, CSRS works with a much longer wavelength than that of CARS, hence uniquely suited to render backward phase matching efficient under high-NA focusing. The side-by-side comparison of epi-CARS and backscattered CSRS imaging in Figure 4 highlights the incredible difference and advantage.

Reviewer #3

(Remarks to the Author)
Acharyya et al. present a theoretical and experimental study of backward detected coherent Stokes Raman scattering (CSRS) in homogeneous media and biological tissues. The authors show that nonlinear wave mixing enables intrinsically backward propagating coherent Raman emission without requiring interfaces or scattering, and identify excitation geometries and polarization conditions that allow efficient epi-detection. The physics is solid and clearly presented. The conceptual framework based on nonlinear momentum conservation is convincing, and the simulations are well supported by experiments. The main novelty lies in enabling backward emission of coherent Raman signals through nonlinear phase matching. This is an interesting concept and provides an alternative approach for epi-detected coherent Raman microscopy.

However, the practical impact of this work remains limited. Whereas the backward CSRS signal is clearly demonstrated, the achieved efficiency (on the order of a fraction of the forward signal) does not appear to provide a substantial performance advantage over existing coherent Raman modalities such as CARS or SRS. Rather than offering a transformative advance, the approach provides an alternative detection geometry that may be useful in specific situations, but it does not fundamentally surpass the capabilities of established techniques.

Comments are listed below:
1. A major concern is the limited cross section of epi-CSRS. Although the authors use a specific excitation geometry to satisfy momentum conservation between the pump, Stokes, and CSRS wavevectors, this advantage is not clearly demonstrated in the Results. In particular, in Fig. 4b and Fig. S1, the CSRS signal appears weaker than CARS. If this is indeed the case, it raises a concern that achieving momentum conservation comes at the cost of reduced sensitivity. The authors are encouraged to provide sensitivity measurements to better support the advantages of their CSRS method. For example, the limit of detection (LOD) of a standard sample (e.g., DMSO) could be measured for direct comparison with CARS microscopy. In addition, intracellular chemical imaging using the CSRS approach should be demonstrated and compared with epi CARS. A quantitative theoretical comparison of the cross sections between CSRS and CARS would also be helpful.
2. In Fig. 3d, authors determine the lateral resolution by imaging bead/oil interface, which is not a sharp edge. The authors should use a sharp edge subject to produce line spread function, then determine the spatial resolution.
3. The authors claim that phase matching in the backward direction allows CSRS to measure bulk signals without edge preference, unlike classic epi CARS. From Fig. 4a, CSRS appears to improve imaging quality, but this should be quantified. The authors are encouraged to measure sub diffraction limited particles (e.g., ~500 nm PMMA beads) to characterize the Green function. Combined with Comment 1, it is important to evaluate whether the sensitivity is sufficient to image small chemical structures.
4. From Fig. 4a, 4d, and S1a, the CSRS images appear non-uniform across the field of view. The authors should clarify whether this is due to the momentum conservation condition. During scanning, does the optimized excitation geometry remain valid? If not, a correction or calibration strategy may be needed to ensure quantitative imaging across a single frame.
5. Fig. 4c and 4d show clear differences between CARS and CSRS in the same field of view. The authors claim that backward CSRS is similar to forward CARS, but this needs stronger experimental support. The authors should provide additional imaging evidence to support this conclusion. It would also be helpful to compare the sensitivity of CSRS and epi CARS in tissue samples.
6. The authors should further clarify the practical situations where backward CSRS offers clear advantages over CARS and stimulated Raman scattering, beyond the lack of transmission access. For example, is it more suitable for imaging opaque samples?
Other minor comments:
1. Important information in the Supplementary Information should be clearly cited in the main text to improve readability.
2. In Section 2 of the main text, the theory used for simulations should be explicitly linked to Supplementary Information Section 2.
3. A more quantitative comparison of signal-to-noise ratio (SNR) with established techniques (CARS and stimulated Raman scattering microscopy) would strengthen the practical impact of the work.

Version 1:

Reviewer comments:

Reviewer #1

(Remarks to the Author)
In the revised manuscript, the authors have nicely addressed all the comments raised in the previous round of review. I therefore recommend the manuscript for publication.

Reviewer #3

(Remarks to the Author)
Nice work!

Open Access This Peer Review File is licensed under a Creative Commons Attribution 4.0 International License, which permits use, sharing, adaptation, distribution and reproduction in any medium or format, as long as you give appropriate credit to the original author(s) and the source, provide a link to the Creative Commons license, and indicate if changes were made.
In cases where reviewers are anonymous, credit should be given to 'Anonymous Referee' and the source.
The images or other third party material in this Peer Review File are included in the article's Creative Commons license, unless indicated otherwise in a credit line to the material. If material is not included in the article's Creative Commons license and your intended use is not permitted by statutory regulation or exceeds the permitted use, you will need to obtain permission directly from the copyright holder.
To view a copy of this license, visit https://creativecommons.org/licenses/by/4.0/

Nature Photonics manuscript NPHOT-2026-02-00352-T
Title: "Intrinsic backward emission of coherent Raman photons from homogeneous media"

Response to the reviewer comments

We thank all the reviewers for their constructive comments, which were taken into account when preparing the revised version of the manuscript.

Summary of revisions and format-related changes
Before the point-by-point responses, we summarize the changes made in the revised manuscript:

- Clarified in the main text and in the Fig. 2 caption that "counterpropagating", "copropagating", and "orthogonal" refer to the relative orientation of the transverse/lateral components of the interacting Stokes wavevectors in the focal angular spectrum.
- Added a resolution discussion based on the calculated local CSRS source distribution and on experimental lateral and axial edge-response measurements at PMMA-oil interfaces. We also changed the corresponding wording from "PSF FWHM" to "line-spread-function FWHM" where the values are derived from edge-spread functions.
- Added a vectorial refocusing analysis in the Supplementary Information and Supplementary Fig. S5. The revised text now states more carefully that the backward-CSRS field supports fiber- or spectrometer-based collection, without implying universal coupling into a specific single-mode fiber.
- Clarified the interpretation of signal strength and sensitivity: the lower backward-CSRS signal is discussed as an optical radiation, phase-matching, collection, transmission and detector issue, not as a smaller molecular Raman cross section. The revised main text also points readers to the photon-flux and SNR analysis in the Supplementary Information.
- Clarified the practical scope of backward CSRS as a same-side, refocusable coherent Raman signal for confocal, fiber-coupled, spectroscopic, heterodyne, or amplified detection schemes, rather than as a general replacement for forward CARS or transmission-detected SRS.
- Updated the links from the main text to the relevant Supplementary Information sections and figures, and revised the affected figure captions. Apart from these marked textual and caption changes, no additional format changes were made.

Reviewer #1 (Remarks to the Author):

This manuscript demonstrates the backward emission of coherent Stokes Raman scattering (CSRS) from homogeneous media for epi-coherent microscopy. In previous coherent nonlinear microscopy techniques, such as second-harmonic generation (SHG), third-harmonic generation (THG), stimulated Raman scattering (SRS), and coherent anti-Stokes Raman scattering (CARS), epi-detection has generally relied either on nanoscopic inhomogeneous signal sources or on multiple scattering in thick samples. In the former case, the signal can be very weak, whereas in the latter case, the spatial coherence of the signal is lost. In the present method, the backward signal is generated by engineering the incident angle and polarization of the Stokes and pump beams in CSRS. Specifically, the Stokes beam has an annular profile and azimuthal polarization at the back aperture of the high-NA objective lens. As a result, owing to the phase-matching condition and dipole radiation from the nonlinear polarization, coherent backward radiation can be produced. An important limitation is that the signal is much weaker than in typical forward-detection CARS experiments. Nevertheless, the authors successfully demonstrate epi-detection CSRS imaging. Considering that this is the first demonstration of epi-detection of a coherent nonlinear signal, which has long been thought to be impossible, I recommend this manuscript for publication, subject to the following minor comments.

We thank Reviewer 1 for the overall positive evaluation of our work. While we fully agree and emphasize in the manuscript that backscattering CSRS results in a weaker photon flux than optimally phase-matched forward-detected CARS, we are able to show detected 1700 Mphotons/s in CSRS from an oil sample. This photon flux is high compared with reported detected photon rates in laser-scanning CARS microscopy. For example, Suhalim et al. (DOI: 10.1002/jbio.201200002) report that the detected CARS signal from a lipid-filled focal volume can exceed 100 photons/µs, corresponding to >100 Mphotons/s. Similarly, Alfonso-García et al. (DOI: 10.1117/1.JBO.19.7.071407) quote a representative detected signal level of approximately 10 photons/µs, corresponding to ~10 Mphotons/s, for high-speed coherent Raman imaging. Thus, despite the intrinsically less favorable backscattering geometry, the photon flux demonstrated here is well within — and substantially above — commonly reported detected-photon benchmarks for laser-scanning CARS microscopy.

C1. I suggest that the authors further clarify the meaning of "counterpropagating" and "copropagating" in Fig. 2. It is not immediately clear that these terms refer to the 'lateral components' of the two interacting Stokes beams.

We thank the reviewer for pointing out this ambiguity. We have clarified both in the main text and in the caption of Fig. 2 that the terms "counterpropagating" and "copropagating" refer to the relative orientation of the lateral/transverse components of the two interacting Stokes wavevectors in the focal angular spectrum, not to the global propagation direction of the excitation beams.

C2. Spatial resolution is an important performance metric in nonlinear microscopy. Nevertheless, the authors report only the lateral spatial resolution. I understand that it may be difficult to rigorously define the point spread function(PSF) of CSRS. However, even a numerical analysis of the product of the electric field distributions, such as $E_S^2(r)E_p(r)$, even at the scalar level, would provide a rough estimate of the spatial resolution. I expect that, owing to the annular Stokes beam, this method may achieve high lateral and axial resolution.

We agree that spatial resolution is an important performance metric and have therefore complemented the source-distribution calculation with experimental axial edge measurements. As a first estimate, we calculated the local effective nonlinear CSRS source distribution, $|P_{cS}(r)|^2 = |\chi^{(3)}:E_S(r)E_p^*(r)E_S(r)|^2$, for a homogeneous sample using the experimentally relevant pupil geometry. The result is shown below. The annular, azimuthally polarized Stokes illumination strongly confines the source laterally, while the axial extent of this local source distribution remains on the order of 1.5-2 µm, with an axial FWHM of approximately 1.7 µm.
We emphasize, however, that this source distribution is not identical to the experimentally measured imaging response. The quantity $|P_{cS}|^2$ describes only the local nonlinear source strength. It does not include coherent Green-function propagation, backward collection, vectorial projection onto the collected field, descanning/refocusing, or the finite detection mode. The experimentally relevant edge response can therefore differ from the local source distribution.
To address this point experimentally, we measured axial edge responses at PMMA-oil interfaces and fitted the resulting edge-spread functions with an error function – the result is shown in the updated version of Fig.3d. The derivative of this fit gives the corresponding axial line-spread function. This analysis yielded an effective axial line-spread-function FWHM of approximately 0.92 µm. We interpret this value as an edge-derived effective axial imaging resolution of the full backward-CSRS detection pathway, rather than as a direct measurement of the local $|P_{cS}|^2$ distribution.
The smaller experimental edge-derived axial FWHM is consistent with the fact that our experimental Stokes illumination is not an ideal binary annulus. The Stokes beam is produced by an axicon pair and corresponds to a thick, intensity-weighted annular distribution with finite inner tails. Numerical edge-response simulations show that the axial FWHM is highly sensitive to the inner Stokes angle: using otherwise identical conditions, we obtain axial FWHM values of 1.218 µm for a 60-80° Stokes annulus, 0.984 µm for 55-80°, and 0.766 µm for 50-80°. The measured value of approximately 0.9-1.0 µm is

therefore compatible with a realistic axicon-generated Stokes ring whose effective angular spectrum contains substantial weight below 60°.

We revised the manuscript accordingly. In the Results section, we now state:

"We further quantified the spatial response from edge measurements at PMMA-oil interfaces. The lateral edge response yields an effective lateral line-spread-function FWHM of approximately 330 nm (Fig. 3d). In addition, axial edge profiles acquired across the PMMA-oil interface yielded an effective axial line-spread-function FWHM of approximately 0.92 µm. These values should be interpreted as edge-derived effective imaging resolutions rather than as a direct measurement of the local nonlinear source distribution alone. In particular, the axial edge response includes coherent CSRS generation, backward propagation, collection through the high-NA objective, and confocal detection."

We also changed the wording in the Fig. 3 caption from "PSF FWHM" to "line-spread-function FWHM" to make clear that the resolution estimate is derived from an edge-spread function.

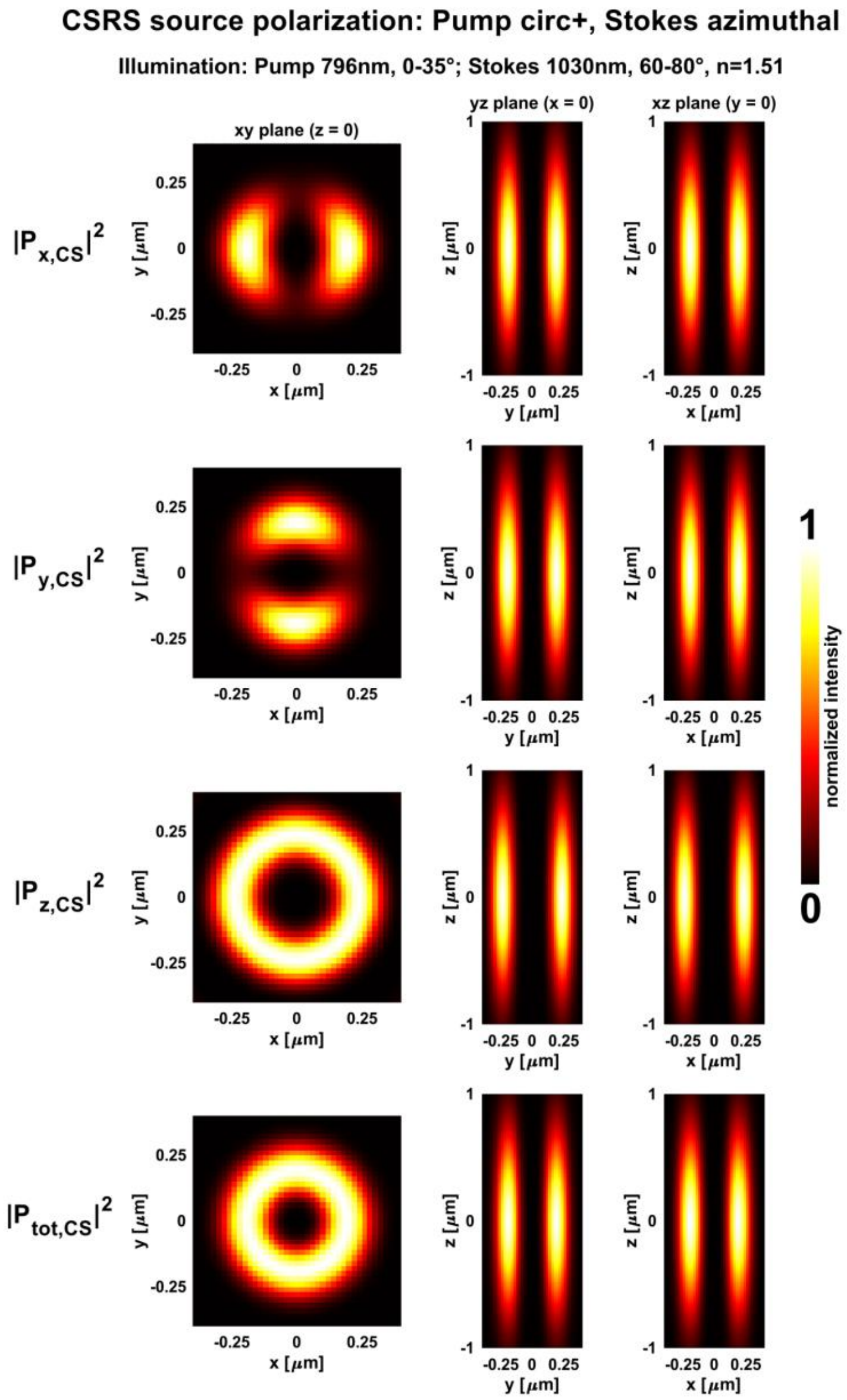

Response Fig. 1. Calculated local effective CSRS source distribution $|P_{CS}|^2$ for the experimentally relevant geometry (pump: circular+, 0°-35°; Stokes: azimuthal, 60°-80°; n = 1.51). The maps show xy, yz, and xz sections of the x-, y-, z-, and total source-polarization intensities. The local source is strongly confined laterally and remains axially localized over approximately 1.5-2 µm. This local source map should be distinguished from the experimentally measured edge-derived imaging response, which also includes coherent propagation, backward collection, refocusing, and detection.

C3. The authors argue that the refocusable nature of the signal is advantageous because the field can be transmitted through an optical fiber. I agree that this is a potentially important advantage. However, I am not sure whether the epi-signal in the present CSRS scheme can actually be coupled into a fiber, given the complex electric field distribution of the epi-detected CSRS signal. In particular, when an azimuthally polarized beam is focused, the resulting focal field can have a complex electric field distribution that may not be compatible with the supported fiber mode. I suggest that the authors further investigate the electric field distribution of the CSRS signal and discuss this point in more detail.

We thank the reviewer for raising this important point. We agree that the total power emitted into the backward collection cone alone is not sufficient to demonstrate practical fiber or spectrometer compatibility. The relevant quantity is the complex vectorial field after collection, descanning, and refocusing by the objective–tube-lens relay.

To address this point, we added a new Supplementary Section, "Vectorial refocusing of the backward-emitted field", together with Supplementary Fig. S5. In this analysis, the computed backward CSRS angular spectrum is propagated through an ideal magnified imaging system using a vectorial Debye–Wolf refocusing formalism. This allows us to evaluate the field distribution that would be formed at a fiber facet or spectrometer entrance plane.

For the experimentally relevant geometry, namely azimuthally polarized annular Stokes illumination combined with a central pump, the simulations show that linear and circular pump polarizations generate a compact, single-lobed refocused intensity distribution. The longitudinal field component is strongly suppressed after magnified refocusing, because the image-side numerical aperture is reduced by the objective–tube-lens magnification. This indicates that the backward CSRS field is not only emitted into the collection cone, but can also be coherently refocused into a well-defined image-plane field suitable for practical same-side detection.

We have nevertheless clarified the limitations of this statement. The mode match to a specific single-mode fiber depends on the detailed polarization and amplitude profile of the refocused field, as well as on the chosen collection optics. In particular, azimuthal pump excitation leads to an annular refocused mode and may require multimode or few-mode collection, additional mode-matching optics, or a spectrometer entrance aperture rather than direct coupling into the fundamental mode of a standard single-mode fiber. We therefore phrase the revised manuscript in terms of support for fiber- or spectrometer-based collection, rather than claiming universal single-mode fiber coupling.

We added the following sentence to the main text:

"In contrast, backward CSRS is generated directly into the backward hemisphere and remains refocusable, enabling true confocal detection. For the experimentally used excitation geometry, our simulations show that the backward CSRS field refocuses after the objective–tube-lens relay into a compact, near-Gaussian intensity distribution, supporting fiber- or spectrometer-based collection (Supplementary Fig. S5)."

We also added a new supplementary section and the following Supplementary Fig. S5:

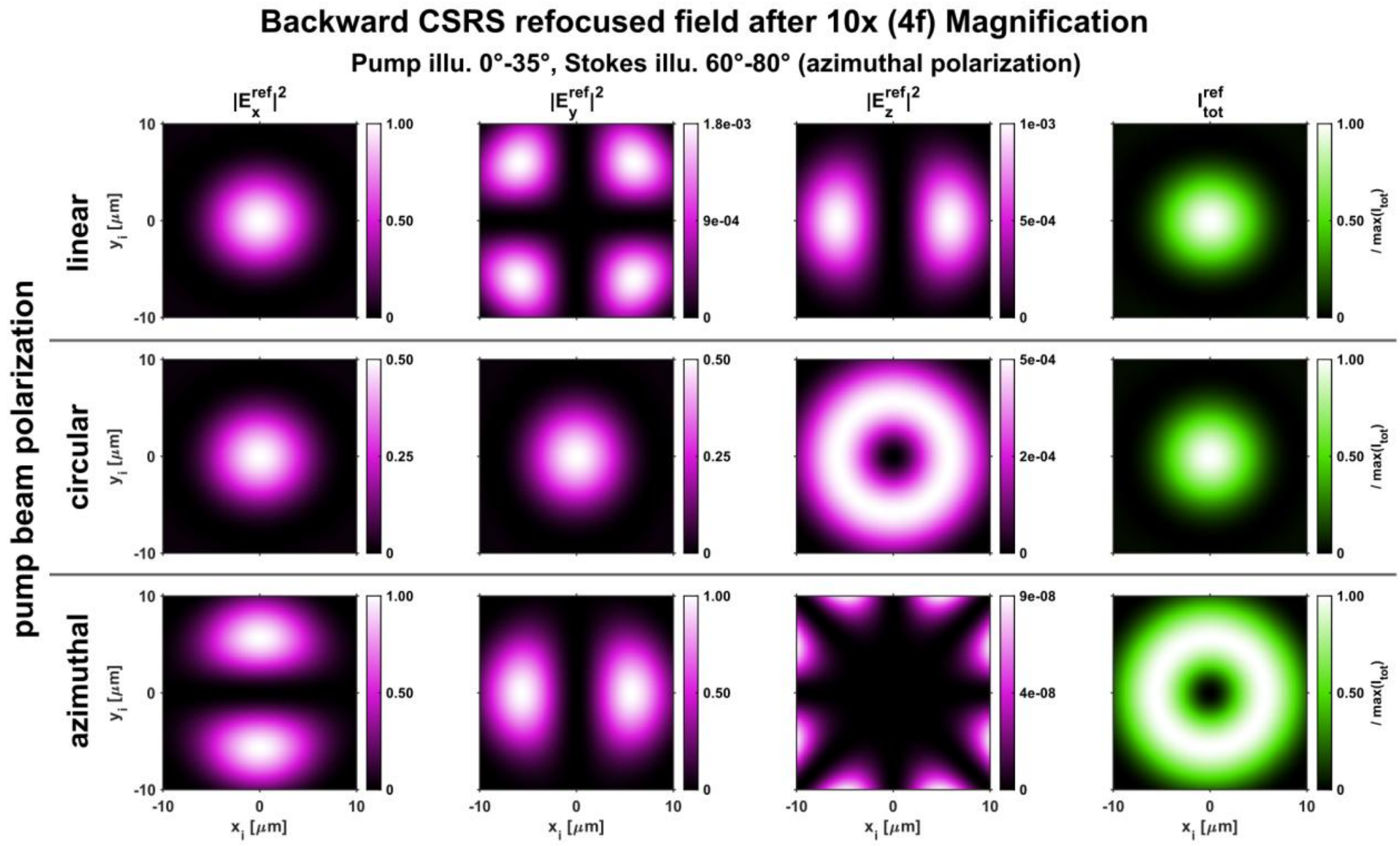

Response Fig. 2 / Supplementary Fig. S5. Vectorial refocusing of the backward-emitted CSRS field. For linear and circular pump excitation, the total refocused field is compact and single-lobed, while the longitudinal component is strongly suppressed. For azimuthal pump excitation, the refocused field becomes annular, indicating a different mode-matching requirement.

Reviewer #2 (Remarks to the Author):

The authors implemented an excitation geometry optimized for the direct generation of backward-directed coherent Stokes Raman scattering (CSRS). This seemingly-straighforward implementation is something not explicitly considered in the literature. The combination of the theoretical analysis presented and the experimental demonstration of biological imaging is convincing. This technique would add to the growing toolbox of nonlinear Raman scattering microscopy.

In the rapid growing field of nonlinear Raman microscopy, while SRS (stimulated Raman scattering) works primarily in the forward direction, CARS (Coherent Anti-Stokes Raman Scattering) has also been occasionally used for background-directed for sub-micron sized objects. However, no one in the Raman imaging field had considered fundamental difference between CSRS and CARS in terms of phase matching condition in the background direction, and simply assumed no particular advantage by CSRS. As pointed out by the authors, CSRS works with a much longer wavelength than that of CARS, hence uniquely suited to render backward phase matching efficient under high-NA focusing. The side-by-side comparison of epi-CARS and backscattered CSRS imaging in Figure 4 highlights the incredible difference and advantage.

We sincerely thank Reviewer 2 for this very positive and encouraging assessment of our work. We are particularly grateful that the reviewer recognizes both the conceptual relevance of the excitation geometry and the fundamental distinction between CSRS and CARS with respect to backward phase matching.

Indeed, a central motivation of our study was to clarify why coherent Stokes Raman scattering, owing to its longer emission wavelength, can access backward-directed phase matching under high-NA focusing in a way that is fundamentally different from coherent anti-Stokes Raman scattering. We are

pleased that the reviewer finds the theoretical analysis and the side-by-side comparison of epi-CARS and backward CSRS imaging convincing.

Reviewer #3 (Remarks to the Author):

Acharyya et al. present a theoretical and experimental study of backward detected coherent Stokes Raman scattering (CSRS) in homogeneous media and biological tissues. The authors show that nonlinear wave mixing enables intrinsically backward propagating coherent Raman emission without requiring interfaces or scattering, and identify excitation geometries and polarization conditions that allow efficient epi-detection. The physics is solid and clearly presented. The conceptual framework based on nonlinear momentum conservation is convincing, and the simulations are well supported by experiments. The main novelty lies in enabling backward emission of coherent Raman signals through nonlinear phase matching. This is an interesting concept and provides an alternative approach for epi-detected coherent Raman microscopy.

We sincerely thank Reviewer 3 for this positive evaluation of our work.

However, the practical impact of this work remains limited. Whereas the backward CSRS signal is clearly demonstrated, the achieved efficiency (on the order of a fraction of the forward signal) does not appear to provide a substantial performance advantage over existing coherent Raman modalities such as CARS or SRS. Rather than offering a transformative advance, the approach provides an alternative detection geometry that may be useful in specific situations, but it does not fundamentally surpass the capabilities of established techniques.

We agree that the present implementation of backward CSRS should not be presented as universally outperforming established forward CARS or SRS microscopy in terms of raw signal efficiency. The main advance of the present work is different: to our knowledge, it is the first experimental demonstration that a homogeneous medium can generate an intrinsically backward-emitted coherent Raman field that remains spatially coherent and refocusable.
This distinction is important because the practical value of backward CSRS is not limited to the detected photon flux in the current microscope implementation. Conventional epi-CARS signals from extended or thick samples are typically produced by a mixture of true backward-emitted CARS from high-spatial-frequency structures and forward-generated CARS that is subsequently scattered or reflected back to the detection side. Such fields can be strong, but they are generally not refocusable into a well-defined image-plane field and therefore cannot be efficiently coupled into a fiber, spectrometer, interferometric detection scheme, or well-defined confocal detection path. In contrast, backward CSRS is generated directly into the backward hemisphere and retains a well-defined optical wavefront.
This refocusable nature opens several practical routes that are difficult or inaccessible with speckled epi-CARS. First, it enables epi-detected broadband coherent Raman spectroscopy, because the backward CSRS field can in principle be coupled into a spectrometer or fiber-based detection path. Second, after descanning, the refocused CSRS field can be combined with a local oscillator for heterodyne detection. This would both amplify the signal above the detector-noise floor and provide access to the background-free imaginary part of the resonant $\chi^{(3)}$ response, corresponding to the information obtained in spontaneous Raman spectroscopy. Third, the refocused field could be amplified or frequency converted after collection, for example by fiber-based Raman or four-wave-mixing gain, thulium-doped fiber amplification, optical parametric amplification, or upconversion to the visible. Finally, because the signal remains image-forming, backward CSRS could support camera-based detection concepts such as image-scanning microscopy, as well as multifocus or wide-field implementations.

We deliberately did not expand the main manuscript into a detailed discussion of these future instrumental directions, because several of them remain beyond the scope of the present experimental demonstration. We have nevertheless strengthened the revised manuscript and Supplementary Information where directly supported by our calculations: in particular, we added a vectorial refocusing analysis (Supplementary Fig. S5) showing that, for the experimentally relevant excitation geometry, the backward CSRS field can refocus into a compact image-plane distribution suitable for fiber- or spectrometer-based collection. We therefore agree with the reviewer that the current implementation is not yet a replacement for established CARS or SRS instruments in all applications. Rather, the work establishes a new physical route to coherent epi-detection from homogeneous media, with practical advantages expected specifically in situations where same-side access, refocusability, spectroscopic coupling, heterodyne detection, or post-collection amplification are required.

Comments are listed below:
C1. A major concern is the limited cross section of epi-CSRS. Although the authors use a specific excitation geometry to satisfy momentum conservation between the pump, Stokes, and CSRS wavevectors, this advantage is not clearly demonstrated in the Results. In particular, in Fig. 4b and Fig. S1, the CSRS signal appears weaker than CARS. If this is indeed the case, it raises a concern that achieving momentum conservation comes at the cost of reduced sensitivity. The authors are encouraged to provide sensitivity measurements to better support the advantages of their CSRS method. For example, the limit of detection (LOD) of a standard sample (e.g., DMSO) could be measured for direct comparison with CARS microscopy. In addition, intracellular chemical imaging using the CSRS approach should be demonstrated and compared with epi CARS. A quantitative theoretical comparison of the cross sections between CSRS and CARS would also be helpful.

We agree that backward CSRS is weaker than optimally phase-matched forward-detected CARS in raw photon yield. However, we believe that a concentration LOD measurement alone would not address the central physical question as directly as the analysis presented in the manuscript. The relevant quantities for this work are (i) the emitted signal predicted by the vectorial CSRS/CARS model, (ii) the experimentally inferred photon flux at the focus and at the detector, and (iii) the detector noise and the resulting theoretical and experimental SNR. This separates the molecular Raman response from optical radiation, phase matching, collection, transmission and detector performance.
On this basis, the lower signal level in our backward-CSRS implementation should not be interpreted as a smaller molecular Raman cross section. Under electronically non-resonant conditions, CARS and CSRS probe the same Raman-active vibrational response and provide equivalent spectral information. Supplementary Fig. S2 quantifies the forward CARS/forward CSRS wavelength dependence under identical excitation conditions: forward CARS is about one order of magnitude stronger in emitted power (at 3000cm^{-1}), corresponding to an approximately fivefold photon-flux difference after accounting for photon energy. The additional reduction for backward CSRS arises from the angular distribution required for backward phase matching and from finite collection and detection efficiencies. These contributions are included in the emitted and detected photon budgets discussed in Supplementary Sections 2 and 3.
We also make explicit that the present implementation is not optimized for very low-concentration LOD measurements. This limitation is currently detector-noise dominated rather than photon-flux limited. For homogeneous sunflower oil, we infer a backward-CSRS photon flux of approximately 7×10^9 photons/s emitted at the focus and approximately 2.1×10^9 photons/s before the detector, values that are high by laser-scanning coherent Raman microscopy standards. Nevertheless, the InGaAs APD detection chain around 1.5 µm remains noisier than visible PMT detection used for CARS. We agree that a concentration-based limit-of-detection measurement, such as a DMSO dilution series, would be a useful future benchmark. In the present revision, however, we chose to emphasize the complete photon-budget and SNR analysis, including an experimentally measured SNR of 49 at an equivalent 1 µs integration time, because this directly separates the emitted CSRS photon flux from the current

detector-noise limitation. The present sensitivity is therefore not fundamentally limited by the backward-CSRS photon flux itself, but by the near-infrared detection chain; importantly, the refocusable nature of the signal provides clear routes to address this limitation through heterodyne detection, frequency conversion, or fiber-based amplification, which would not be available for a speckled, non-refocusable epi-CARS field.

We have revised the manuscript accordingly:

Within the Results section:
"Figure 4a shows backscattered CSRS images of PMMA beads dispersed in sunflower oil. The corresponding spectra (Fig. 3b) confirm that, in the absence of electronic resonances, CARS and CSRS provide nearly interchangeable vibrational information. Notably, strong CSRS signals are obtained from homogeneous regions of sunflower oil, well away from any bead-oil or oil-substrate interfaces."

Within the Discussion section:
"Importantly, this photon-flux difference should not be interpreted as a smaller molecular cross section for CSRS. Under electronically non-resonant conditions, CARS and CSRS are governed by the same Raman-active vibrational response and provide equivalent spectral information. The lower photon flux obtained for backward CSRS instead reflects optical factors, including the longer generated wavelength, the backward phase-matching geometry, and finite collection and detection efficiencies, which are included in the emitted and detected photon budgets quantified in Supplementary Sections 2 and 3."

Within the Supplementary Information, we clarified the interpretation of Supplementary Fig. S2:
"Across the CH-stretch region, the ratio P_{CARS}/P_{CSRS} remains close to 10 and shows little dependence on NA. This difference is not introduced as a different molecular Raman cross section. In electronically non-resonant samples, CARS and CSRS are governed by the same resonant Raman susceptibility and therefore contain equivalent vibrational information. The simulated power ratio instead reflects the different radiation wavelength and the finite spatial extent of the coherent Raman source."

C2. In Fig. 3d, authors determine the lateral resolution by imaging bead/oil interface, which is not a sharp edge. The authors should use a sharp edge subject to produce line spread function, then determine the spatial resolution.

We agree that a sharp knife-edge or line-spread-function measurement is the ideal experimental resolution benchmark. We attempted such measurements with classical sharp-edge samples, including oil/water interfaces between coverslips. In practice, these samples did not provide a reliable edge for our geometry: water forms a meniscus at the glass interface, and refractive-index mismatch across the interface introduces additional aberrations that distort the edge response.
After testing several alternatives, we found that the most reproducible and least aberrating edge was provided by large PMMA beads embedded in sunflower oil. This geometry offers two advantages. First, the refractive indices are sufficiently close to minimize optical distortion. Second, the bead diameter is 20 µm, whereas the measured lateral transition is only about 0.33 µm; over this sub-micrometre scale, the bead surface is locally flat to an excellent approximation. The scale sketch below illustrates that the effective CSRS source/PSF is small compared with the bead radius.
Therefore, we use the bead/oil interface in Fig. 3d as a practical knife-edge approximation for estimating the lateral resolution. In the revision, we also added axial edge measurements at PMMA-oil interfaces. Fitting these axial edge-spread functions and taking the derivative yielded an effective axial line-spread-function FWHM of approximately 0.9-1.0 µm. We agree that these measurements do not constitute a full experimental CSRS Green-function measurement, but they provide robust and experimentally accessible lateral and axial resolution estimates under conditions with minimal

refractive-index artifacts. We have therefore changed the manuscript wording from “PSF FWHM” to “line-spread-function FWHM” where the resolution is derived from an edge response.

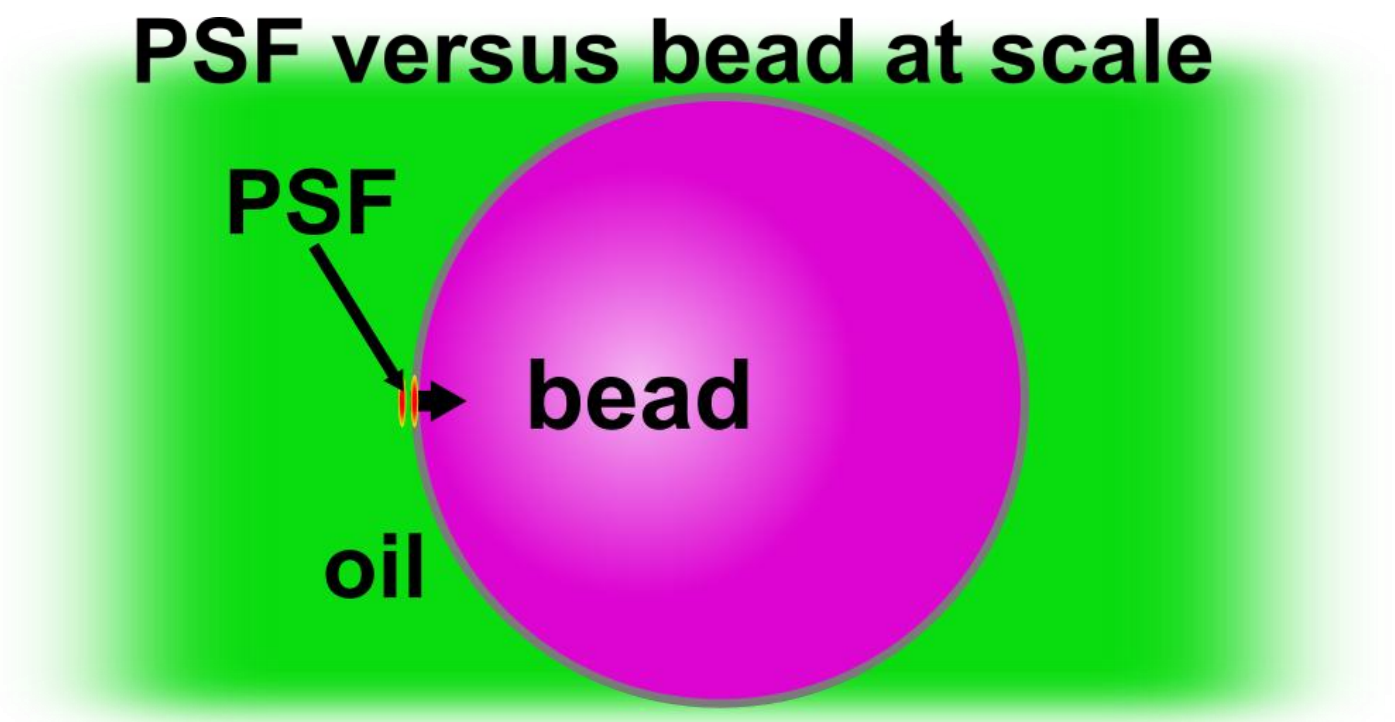

Response Fig. 3: Scale comparison of the effective CSRS source/PSF with a 20 µm PMMA bead in oil. The bead surface is effectively flat over the sub-micrometre lateral extent used to determine the edge response.

C3. The authors claim that phase matching in the backward direction allows CSRS to measure bulk signals without edge preference, unlike classic epi CARS. From Fig. 4a, CSRS appears to improve imaging quality, but this should be quantified. The authors are encouraged to measure sub diffraction limited particles (e.g., ~500 nm PMMA beads) to characterize the Green function. Combined with Comment 1, it is important to evaluate whether the sensitivity is sufficient to image small chemical structures.

We agree that the claim of bulk-sensitive backward CSRS should be supported quantitatively, and we have clarified the corresponding evidence in the revised manuscript.
First, the absence of edge preference is not inferred only from the visual appearance of Fig. 4a. In the bead-in-oil sample, strong CSRS signal is obtained from homogeneous sunflower oil regions well away from bead-oil and oil-substrate interfaces. Moreover, the PMMA beads do not show the shadowing or edge-highlighting effects that are typical for epi-CARS contributions generated by high spatial frequencies at interfaces. In addition, the axial CSRS profile in homogeneous sunflower oil follows the depth dependence of two-photon fluorescence excited at the pump and Stokes wavelengths, supporting that the observed axial signal loss is dominated by the optical performance of the TIRF objective rather than by an interface-specific CSRS contribution.

We have clarified and strengthened this point in the Results section. The revised manuscript now states:

“Notably, strong CSRS signals are obtained from homogeneous regions of sunflower oil, well away from any bead–oil or oil–substrate interfaces. In contrast to epi-CARS, the PMMA beads do not exhibit interface-related shadowing or highlighting effects, consistent with the absence of high-pass filtering in backward CSRS.”

In addition, we added the following clarification when discussing tissue imaging:

“In contrast, backward CSRS is generated directly into the backward hemisphere by nonlinear phase matching and therefore provides bulk chemical contrast without relying on high-pass spatial filtering or scattering-mediated return.”

Second, to address the reviewer’s suggestion as directly as possible, we performed additional small-particle measurements under the same standard acquisition conditions used for the images in the manuscript, namely an effective pixel dwell time of 20 µs. Representative data are shown in Response

Fig. 4. These measurements show that micrometre-sized beads can still be detected by backward CSRS, but they also confirm the practical limitation of the present implementation: sub-micrometre particles do not provide a sufficiently robust, isolated, high-SNR response from which a quantitative experimental Green function could be extracted under standard imaging conditions. We therefore did not use these data as a quantitative PSF or Green-function measurement. Instead, they serve as an experimental control showing that we attempted the reviewer's proposed measurement and that the limitation is set by the present near-infrared detection chain rather than by a lack of backward-CSRS signal.

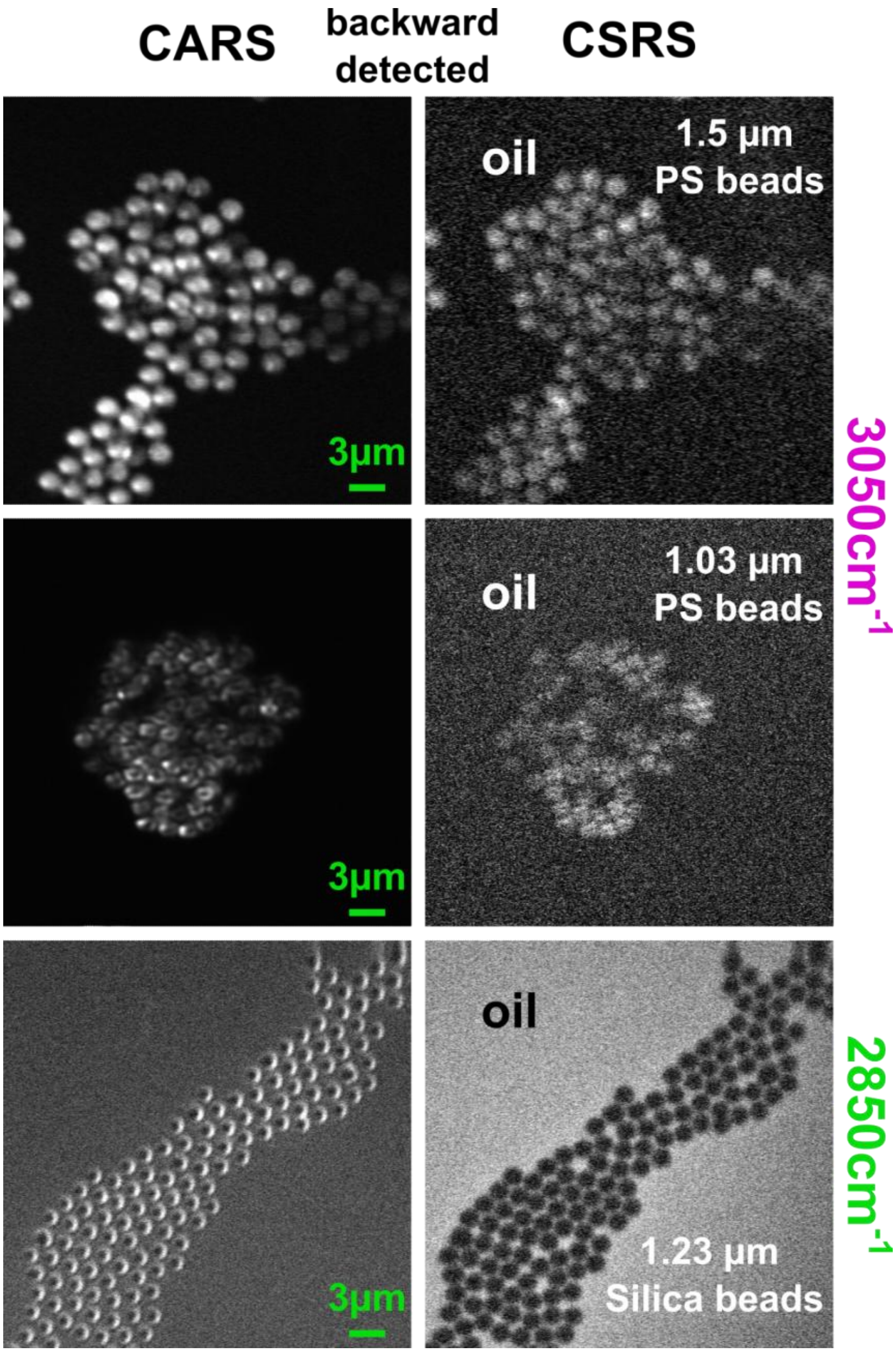

Response Fig. 4. Attempted small-particle coherent Raman measurements under standard imaging conditions (20 µs effective pixel dwell time). The panels show 1.5 µm (Thermofisher, 37275) and 1.03 µm (Agar Scientific, S130-7) PS beads at 3050 cm−1 and 1.23 µm (Spherotech, SIP-10-10) silica beads in oil at 2850 cm−1. Micrometre-sized particles can be detected, but the backward-CSRS channel is too noisy and the objects are not sufficiently point-like to extract a quantitative Green function. This control shows that measurements on 500 nm or approximately 100 nm particles are beyond the practical sensitivity of the present detection chain under standard imaging conditions.

This experimental observation is also consistent with the expected signal scaling. We agree that a sub-diffraction particle would be the most direct way to measure the full experimental Green function. However, such a measurement is considerably more demanding for coherent Raman microscopy than for fluorescence microscopy. The coherent Raman field amplitude scales approximately with the number of coherently driven molecular scatterers, and the detected intensity therefore scales approximately with the square of this number. A 500 nm bead is already comparable to, or larger than, the measured lateral line-spread-function width of approximately 0.33 µm and is therefore not sufficiently point-like for a true Green-function measurement. A more appropriate Green-function measurement would require substantially smaller particles, ideally on the order of 100 nm.
We can estimate the expected signal level from our calculated effective nonlinear source distribution. Using an effective CSRS source volume with approximate FWHM dimensions of 0.4 µm x 0.4 µm laterally and 1.7 µm axially, a 100 nm bead samples only about 3.7×10^{-3} of this volume. Because the coherent Raman intensity scales approximately with the square of the number of scatterers, the expected signal from such a bead would be reduced by roughly $(3.7 \times 10^{-3})^2$, i.e. about 1.4×10^{-5}, compared with the homogeneous bulk signal of similar Raman density. Starting from the experimentally measured backward-CSRS SNR of 49 at an equivalent 1 µs integration time in sunflower oil, this would correspond to an SNR far below unity even at the 20 µs dwell time used for the manuscript images. Reaching an SNR suitable for a particle-derived Green-function measurement would require integration times far beyond the standard imaging conditions, before accounting for additional losses from bead positioning, imperfect overlap, material-dependent Raman efficiency, and detector-noise limitations. For this reason, and consistent with the attempted small-particle measurements shown in Response Fig. 4, we used PMMA-oil edge responses as practical and experimentally robust resolution benchmarks rather than claiming a particle-derived Green-function measurement. As explained in our response to Comment 2, the 20 µm bead radius is much larger than the measured 0.33 µm lateral transition width, so the bead surface is locally flat over the relevant scale and acts as a practical knife-edge. In addition, the newly measured axial edge responses yield an effective axial line-spread-function FWHM of approximately 0.92 µm. We agree that these measurements do not provide a full experimental Green-function measurement. However, they quantitatively support the lateral and axial resolution estimates, while the homogeneous-oil signal, axial depth dependence, and absence of interface shadowing/highlighting support the central claim that backward CSRS can access bulk chemical signal without the edge preference characteristic of epi-CARS.

C4. From Fig. 4a, 4d, and S1a, the CSRS images appear non-uniform across the field of view. The authors should clarify whether this is due to the momentum conservation condition. During scanning, does the optimized excitation geometry remain valid? If not, a correction or calibration strategy may be needed to ensure quantitative imaging across a single frame.

We thank the reviewer for pointing this out. We agree that the field-of-view non-uniformity visible in the raw CSRS images should be separated from the question of whether the optimized backward phase-matching geometry remains valid during scanning.
We therefore estimated how much the full angular aperture of the excitation cone changes over the field of view. The nominal half-angle of the objective is $\Theta_0 = \mathrm{asin}(NA/n) = \mathrm{asin}(1.49/1.515) = 79.58°$, so the full angular aperture in the field center is $\alpha_{center} = 2\Theta_0 = 159.15°$. Using the approximate pupil diameter of the last objective lens, D = 1.5 mm, i.e. R = 0.75 mm, this corresponds to an effective geometrical distance $L = R/\tan(\Theta_0) = 0.138$ mm. For the 72 µm field of view used in the experiments, the half-field displacement is x = 36 µm. At the field edge, the angles to the two opposite pupil edges are $\Theta_1 = \mathrm{atan}((R-x)/L) = 79.06°$ and $\Theta_2 = \mathrm{atan}((R+x)/L) = 80.04°$. The relevant full aperture is therefore $\alpha_{center} = \Theta_1 + \Theta_2 = 159.11°$, i.e. only 0.05° smaller than in the field center. Even at a field corner (diagonal), using $x = \sqrt{2} \times 36$ µm, the reduction remains only about 0.09°.

This angular change is negligible compared with the deliberately used excitation ranges, namely the 60°-80° annular Stokes illumination and the 0°-35° central pump illumination. We therefore do not attribute the observed field-of-view variation to a breakdown of the backward phase-matching condition. To first order, the optimized excitation geometry remains valid during scanning over the imaged field of view.
The more likely origin is conventional field-dependent shading/vignetting in a high-NA, two-color laser-scanning microscope. Several effects contribute to this and the most important ones in the CSRS case are: scan-angle-dependent clipping or transmission in the relay/objective pupil and chromatic field-dependent changes of the pump-Stokes overlap. These effects are not specific to backward CSRS. Uneven illumination and mosaicking artefacts are a well-known issue in nonlinear and multimodal microscopy, and dedicated correction and stitching strategies have been developed for precisely this class of problem (our contributions for example Legesse et al., J. Microsc. 2015, DOI: 10.1111/jmi.12236; Chernavskaia et al., J. Chemometrics 2017, DOI: 10.1002/cem.2901/10.1002/cem.2908).

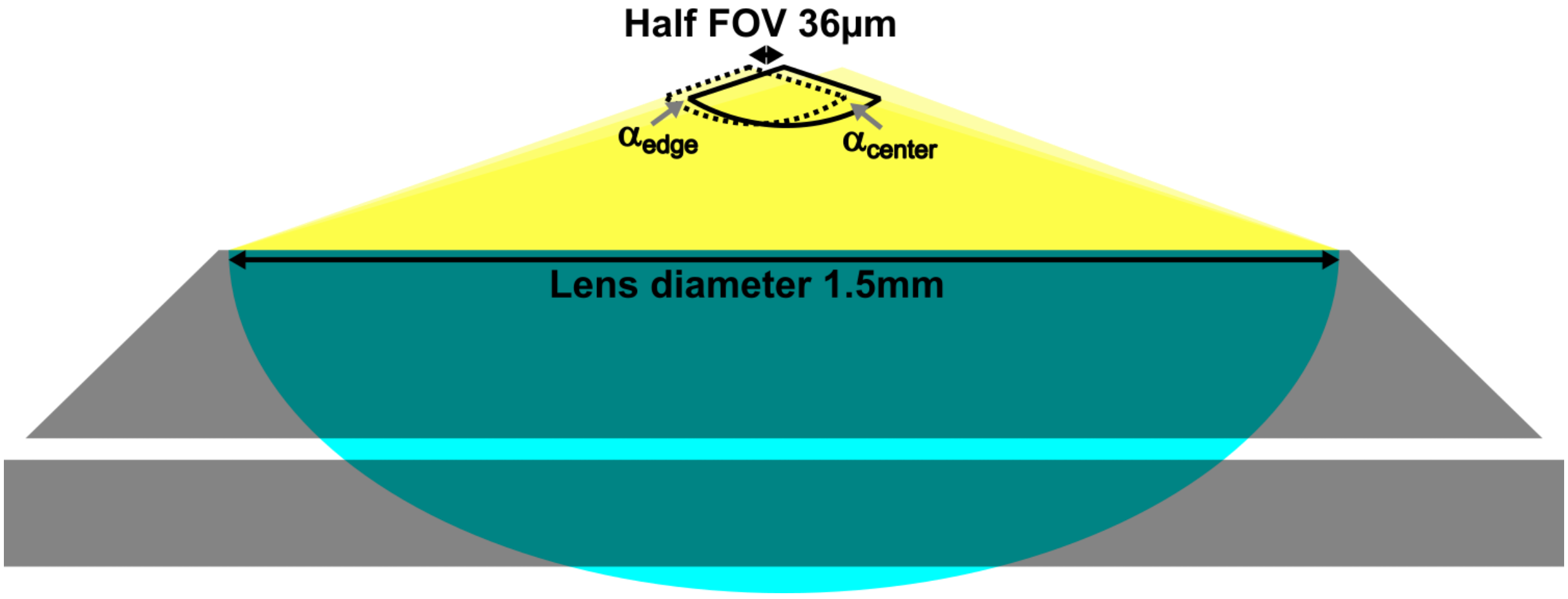

Response Fig. 5. Visualization of the relative sizes of the lens diameter (1.5 mm) and the half size of the FOV (36 µm). Laser scanning over the full FOV does not reduce significantly the angular spread of the incoming illumination.

C5. Fig. 4c and 4d show clear differences between CARS and CSRS in the same field of view. The authors claim that backward CSRS is similar to forward CARS, but this needs stronger experimental support. The authors should provide additional imaging evidence to support this conclusion. It would also be helpful to compare the sensitivity of CSRS and epi CARS in tissue samples.

We thank the reviewer for raising this point. We agree that the comparison between epi-CARS, forward-CARS, and backward CSRS requires clearer experimental support and a more careful wording. We therefore added an additional response figure showing raw, only autoscaled images of the same tissue regions recorded in epi-CARS, forward-CARS, and backward-CSRS detection.

These new data show that all three channels recover the same dominant lipid- and myelin-rich structures. The images are not pixel-identical, but the differences are moderate and do not indicate a fundamentally different chemical contrast mechanism. Rather, they are consistent with the different spatial transfer functions and collection pathways of the three detection geometries. Forward-CARS provides the conventional forward-generated coherent Raman signal. Epi-CARS in tissue contains a mixture of true backward-emitted CARS generated by high-spatial-frequency structures near the focus and originally forward-emitted CARS that is scattered or reflected back toward the objective. Backward CSRS, in contrast, is generated directly into the backward hemisphere by nonlinear phase matching.

Therefore, all three channels probe the same Raman-active susceptibility, but local image contrast can still differ because of interfaces, scattering, sample reflectivity, collection efficiency, and return-path efficiency.

This interpretation is also supported by previous tissue CARS measurements. Wang et al. directly compared forward-detected CARS and epi-detected CARS images of axonal myelin in spinal cord tissue. Their Fig. 2 shows that the two CARS channels provide related but visibly different myelin contrast and line profiles, even though both were recorded from the same vibrational resonance (Biophysical Journal 89, 581–591, 2005; DOI: 10.1529/biophysj.105.061911). We now cite this work in the main text and explicitly state that the differences in Fig. 4c,d are expected from the different spatial transfer functions of epi-CARS and backward CSRS.

Regarding sensitivity, we agree that this should not be inferred from tissue contrast alone, because tissue images mix molecular response with optical transport, scattering, reflectivity, and collection efficiency. We therefore quantify the sensitivity of backward CSRS using homogeneous sunflower oil, where structural scattering and interface effects do not dominate the signal. The Supplementary Information provides a detailed photon-budget and SNR analysis, including emitted photon flux, detected photon flux, detector noise, and experimentally measured SNR. This analysis yields approximately 7×10^9 backward-CSRS photons/s emitted from the focal volume and an experimentally measured SNR of 49 at an equivalent 1 µs integration time. It also shows that the practical sensitivity difference between CARS and backward CSRS in the present implementation is governed not only by photon-flux differences, but also by the higher detector noise of the near-infrared InGaAs APD detection chain compared with visible PMT-based CARS detection.

Thus, the added raw comparison images support that backward CSRS provides coherent Raman contrast from the same tissue structures as CARS, while the remaining contrast differences are expected from the different detection geometries. The sensitivity comparison is addressed separately and quantitatively through the homogeneous-sample photon-budget and SNR analysis.

We added to the manuscript:

"Notably, the brightness difference between lipid-rich regions and surrounding tissue is reduced in epi-CARS compared to backward CSRS. This difference is consistent with the distinct spatial transfer functions of the two epi-detected signals. In thick tissue, epi-CARS generally contains a mixture of true backward-emitted CARS generated by high-spatial-frequency structures near the focus and originally forward-emitted CARS that is subsequently scattered or reflected back toward the objective. Consequently, epi-CARS images can be influenced by interfaces, local scattering, sample reflectivity, and return-path efficiency. This behavior has been observed directly in spinal cord tissue, where forward- and epi-detected CARS provide related but visibly different myelin contrast and line profiles [16]. In contrast, backward CSRS is generated directly into the backward hemisphere by nonlinear phase matching and therefore provides bulk chemical contrast without relying on high-pass spatial filtering or scattering-mediated return."

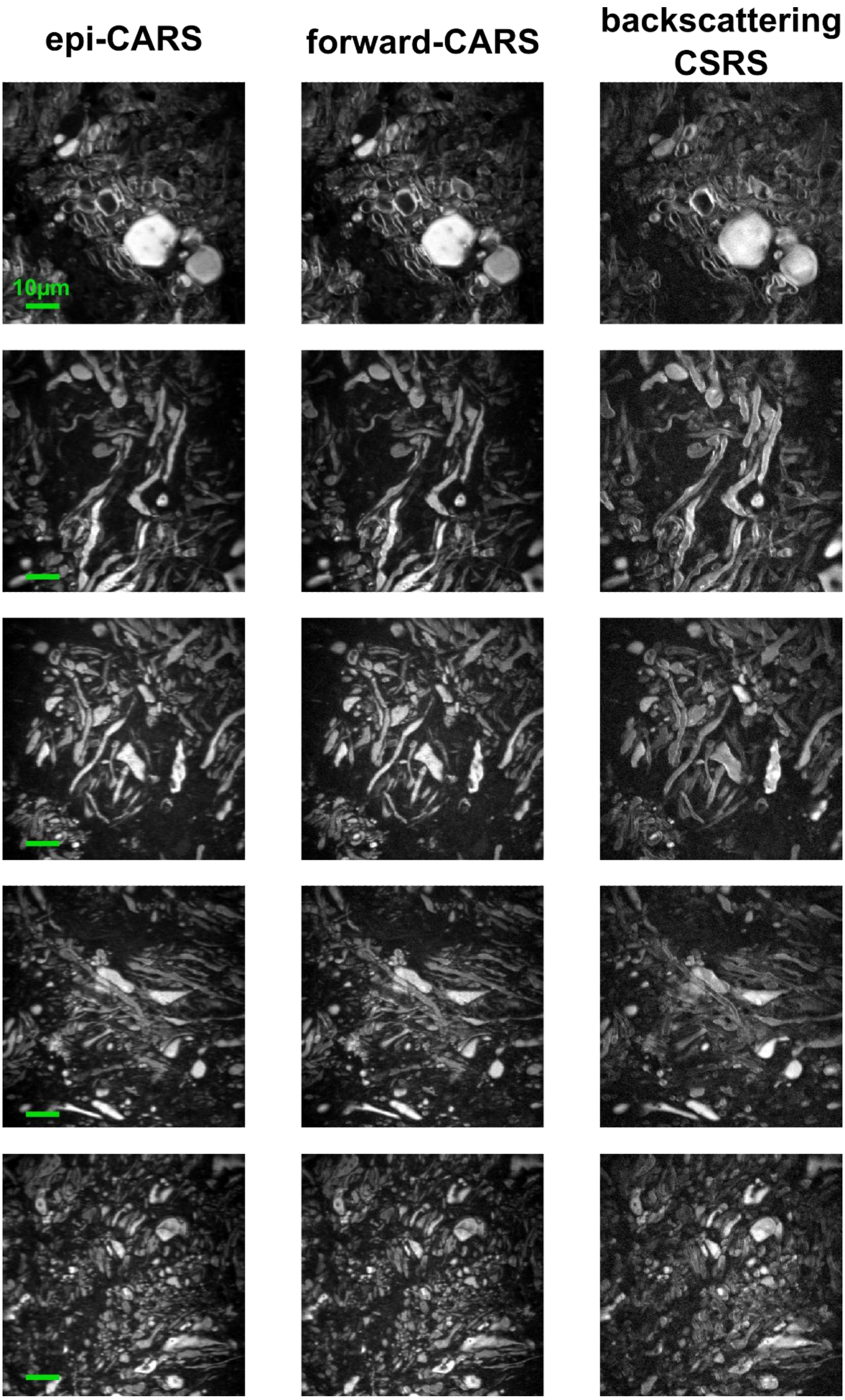

Response Fig. 6. Direct comparison of epi-CARS, forward-CARS, and backward-CSRS tissue images. The panels show raw, only autoscaled images of the same fields of view recorded at the CH-stretch resonance. All three channels recover the same dominant lipid- and myelin-rich structures, while local contrast differences remain visible. These differences are expected because epi-CARS, forward-CARS, and backward CSRS have different spatial transfer functions, collection geometries, and sensitivity to scattering, reflectivity, and return-path efficiency.

C6. The authors should further clarify the practical situations where backward CSRS offers clear advantages over CARS and stimulated Raman scattering, beyond the lack of transmission access. For example, is it more suitable for imaging opaque samples?

We agree that backward CSRS should not be presented as a universal replacement for established CARS or SRS microscopy. If transmission access is available and only single-frequency imaging is required, forward CARS and transmission-detected SRS remain highly efficient and technically mature modalities. The practical advantage of backward CSRS arises in a more specific class of experiments: situations in which only same-side optical access is available, or in which a coherent Raman signal must be refocused into a well-defined detection plane for confocal detection, fiber coupling, spectroscopy, heterodyne detection, or post-collection amplification.

An important example is spectroscopic laser-scanning coherent Raman microscopy. Forward CARS and SRS can of course be implemented spectroscopically, but efficient coupling of a scanned forward signal into a fixed spectrometer or fiber generally requires a separate transmission-side collection and descanning path that reproduces the scan geometry of the excitation path. This additional forward optical train is costly, alignment-sensitive, and difficult to implement in many microscopes or sample geometries. Backward CSRS offers a practical advantage here because the signal is generated directly into the backward hemisphere, collected by the same objective, descanned by the same scanning optics, and can then be refocused on the illumination side into a fiber, spectrometer entrance slit, or interferometric detection path.

This advantage is distinct from simply lacking transmission access. It also concerns the quality and usability of the detected optical field. In extended or scattering samples, epi-CARS generally contains a mixture of true backward-emitted CARS from high-spatial-frequency structures and originally forward-emitted CARS that is scattered or reflected back to the objective. Such a field can be useful for imaging, but it is generally not refocusable into a well-defined spectroscopic or interferometric detection mode. Backward CSRS, in contrast, is generated directly into the backward hemisphere by nonlinear phase matching and remains spatially coherent and refocusable.
Regarding opaque samples, backward CSRS is advantageous when the sample is opaque to transmission-side collection but still allows the pump and Stokes beams to form a focus at the region of interest. It does not remove the general depth limitation imposed by excitation scattering or aberrations in highly opaque media. We have clarified this point in the revised manuscript by emphasizing that the main practical niche of backward CSRS is same-side, refocusable coherent Raman detection, particularly for confocal, fiber-coupled, spectroscopic, heterodyne, or amplified detection schemes.

We added to the manuscript:

"Although backward CSRS emits fewer photons than forward CARS, the combination of favorable detector quantum efficiencies and refocusable backward emission enables efficient confocal detection, fiber coupling, spectroscopic analysis, and point-spread-function characterization. Its practical niche is therefore not to replace forward CARS or transmission-detected SRS when transmission access is available, but to provide a directly generated and refocusable coherent Raman signal for same-side detection. This is particularly relevant for samples on opaque substrates, thick specimens, tissue biopsies, and fiber- or spectrometer-based implementations. In laser-scanning spectroscopy, backward CSRS also avoids the need for a separate transmission-side descanned detection path, which is otherwise required to efficiently couple scanned forward CARS or SRS signals into a fixed fiber or spectrometer entrance. This work establishes backward CSRS as a new contrast mechanism for coherent Raman microscopy and opens a pathway toward robust, epi-detected, and

fiber-compatible Raman imaging platforms for applications where transmission-based detection is impractical or technically cumbersome.”

Other minor comments:
C1. Important information in the Supplementary Information should be clearly cited in the main text to improve readability.

We have revised the main text to make the links to the Supplementary Information more explicit at the points where supplementary material is essential for interpreting the results. In particular, we now explicitly refer to Supplementary Information, Section 2 when introducing the vectorial simulations used for Fig. 2; to Supplementary Fig. S1 and Supplementary Information, Section 1 when discussing the raw images and fluorescence controls; and to Supplementary Information, Section 3 together with Supplementary Fig. S6 when discussing the photon budget, detector noise, and experimental SNR. These additions should make the main text easier to follow without requiring the reader to search through the Supplementary Information.

C2. In Section 2 of the main text, the theory used for simulations should be explicitly linked to Supplementary Information Section 2.

We have revised Section 2 of the main text to explicitly link the vectorial simulations used for the momentum-space analysis to Supplementary Information, Section 2. The corresponding sentence now reads: "To substantiate these trade-offs, we performed full vectorial simulations of the focused pump and Stokes fields for isotropic samples and computed the resulting CSRS emission (Supplementary Information, Section 2)."

C3. A more quantitative comparison of signal-to-noise ratio (SNR) with established techniques (CARS and stimulated Raman scattering microscopy) would strengthen the practical impact of the work.

We thank the reviewer for raising this point. We agree that the practical impact of backward CSRS should be supported by a quantitative SNR comparison. We have therefore clarified in the main text that the Supplementary Information contains a detailed photon-budget and SNR analysis for backward CSRS and forward CARS under matched laser-scanning conditions.
Experimentally, we obtain an SNR of 49 for backward CSRS and an SNR of 171 for forward CARS at an equivalent 1 µs integration time in homogeneous sunflower oil at 2850 cm^{-1}, as shown in Supplementary Fig. S6. In addition, Supplementary Information, Section 3 and Table S1 quantify the emitted photon flux, detected photon flux, detector noise, and noise-equivalent photon flux. This analysis shows that the current practical sensitivity difference between forward CARS and backward CSRS is not only due to the lower backward-CSRS photon flux, but also to the higher detector noise of the near-infrared InGaAs APD detection chain compared with visible PMT-based CARS detection.
A direct experimental SRS benchmark under equivalent conditions is less straightforward in the present setup. SRS is not detected as a newly emitted photon flux, but as a small intensity modulation on a bright transmitted excitation beam. Its sensitivity therefore depends strongly on laser relative-intensity noise, modulation frequency, balanced detection, and the modulation/demodulation scheme. In addition, our present experiment uses AOM-based pulse picking to reduce the effective repetition rate and increase the number of coherent Raman photons per laser pulse. This configuration is well suited for CARS/CSRS voltage-readout measurement, but it is not optimized for low-noise lock-in SRS detection. Performing an equivalent SRS benchmark would therefore require a separately optimized low-RIN modulation (i.e. no AOM modulation on the detected beam) and detection scheme. We nevertheless note that shot-noise-limited SRS microscopy has been shown theoretically to have a sensitivity comparable to CARS microscopy (Ozeki et al., Opt. Express 17, 3651–3658, 2009; DOI: 10.1364/OE.17.003651). Thus, our measured forward-CARS SNR provides a meaningful benchmark for

established forward coherent Raman modalities, while the measured backward-CSRS SNR directly quantifies the sensitivity of the new same-side, refocusable detection geometry.

We added the following sentence to the Discussion section:

"Experimentally, we obtain an SNR of 49 for backward CSRS and 171 for forward CARS at an equivalent 1 µs integration time in homogeneous sunflower oil at 2850 cm^{-1} (Supplementary Information, Section 3 and Supplementary Fig. S6), suggesting that the present backward-CSRS implementation is still detector-noise dominated."
